# Synthesis, Antitumor Activity, and Docking Analysis of New Pyrido[3′,2′:4,5]furo(thieno)[3,2-*d*]pyrimidin-8-amines

**DOI:** 10.3390/molecules24213952

**Published:** 2019-10-31

**Authors:** Samvel N. Sirakanyan, Domenico Spinelli, Athina Geronikaki, Elmira K. Hakobyan, Harutyun Sahakyan, Erik Arabyan, Hovakim Zakaryan, Lusine E. Nersesyan, Anahit S. Aharonyan, Irina S. Danielyan, Rafayel E. Muradyan, Anush A. Hovakimyan

**Affiliations:** 1 Scientific Technological Center of Organic and Pharmaceutical Chemistry of National Academy of Science of Republic of Armenia, Institute of Fine Organic Chemistry of A.L. Mnjoyan, 0014 Yerevan, Armenia; hakobyan.elmira@mail.ru (E.K.H.); lusinenersesyan@gmail.com (L.E.N.); AAaharonyan@gmail.com (A.S.A.); irina_danielyan@mail.ru (I.S.D.); muradyanraf@inbox.ru (R.E.M.); aaa.h.87@mail.ru (A.A.H.); 2 Dipartimento di Chimica G. Ciamician, Alma Mater Studiorum-Università di Bologna, Via F. Selmi 2, 40126 Bologna, Italy; 3 School of Pharmacy, Aristotle University of Thessaloniki, 54124 Thessaloniki, Greece; geronik@pharm.auth.gr; 4 Department of Bioengineering, Bioinformatics and Molecular Biology, Russian-Armenian University, 0051 Yerevan, Armenia; sahakyanhk@gmail.com; 5 Institute of Molecular Biology of NAS, Hasratyan 7, 0014 Yerevan, Armenia; ear20@mail.ru (E.A.); h_zakaryan@mb.sci.am (H.Z.)

**Keywords:** pyrido[3′,2′:4,5]furo(thieno)[3,2-*d*]pyrimidin-8-amines, amination, furo[2,3-*e*]imidazo[1,2-*c*]pyrimidine, furo[2,3-*e*]pyrimido[1,2-*c*]pyrimidine, DNA methylation, sarcoma 180, antitumor activity

## Abstract

Continuing our research in the field of new heterocyclic compounds, herein we report on the synthesis and antitumor activity of new amino derivatives of pyrido[3′,2′:4,5](furo)thieno[3,2-*d*]pyrimidines as well as of two new heterocyclic systems: furo[2–*e*]imidazo[1,2-*c*]pyrimidine and furo[2,3-*e*]pyrimido[1,2-*c*]pyrimidine. Thus, by refluxing the 8-chloro derivatives of pyrido[3′,2′:4,5]thieno(furo)[3,2-*d*]pyrimidines with various amines, the relevant pyrido[3′,2′:4,5]thieno(furo)[3,2-*d*]pyrimidin-8-amines were obtained. Further, the cyclization of some amines under the action of phosphorus oxychloride led to the formation of new heterorings: imidazo[1,2-*c*]pyrimidine and pyrimido[1,2-*c*]pyrimidine. The possible antitumor activity of the newly synthesized compounds was evaluated in vitro. The biological tests evidenced that some of them showed pronounced antitumor activity. A study of the structure–activity relationships revealed that the compound activity depended mostly on the nature of the amine fragments. A docking analysis was also performed for the most active compounds.

## 1. Introduction

Enzymatic DNA methylation by DNA methyltransferases is an important constituent of the cell epigenetic regulatory system that modulates gene expression without altering the DNA base sequence. It is tissue- (cell) and age-specific and is involved in the regulation of all genetic functions, including transcription, DNA replication and repair, gene transposition, and cell and sex differentiation. The methylation pattern of DNA is inherited, and significant distortions result in defects in growth and development. There is no doubt that some changes in DNA methylation induce cancer, premature aging, apoptosis, and death [1]. Malignant cells have a different DNA methylation pattern and a different set of expressed DNA methyltransferase activities compared to normal cells [2]. De novo methylation of CpG islands in the promoter regions of tumor-suppressor genes may lead to transcriptional silencing through a complex process involving histone deacetylation and chromatin condensation, and thus it represents a tumorigenic event that is functionally equivalent to genetic changes such as mutation and deletion [3]. The aberrant methylation of promoters has already been described in hundreds of genes associated with tumorigenesis and could serve as an important biomarker if new methods applicable to clinical practice are sufficiently advanced [4]. The DNA methylation profile of genomes or separate genes already serves as a true marker of different cancer varieties even at the early stages of carcinogenesis [1]. DNA methylation biomarkers with diagnostic, prognostic, and predictive power are already in clinical trials [5,6]. DNA methylation is interesting from a diagnostic viewpoint because it may be easily detected in DNA released from neoplastic and preneoplastic lesions into sera, urine, or sputum; and from a therapeutic viewpoint because epigenetically silenced genes may be reactivated by inhibitors of DNA methylation and/or histone deacetylase. A better understanding of epigenetic mechanisms leading to tumor formation and chemoresistance may eventually improve current cancer treatment regimens and be instructive for a more rational use of anticancer agents [3,7].

Data in the literature have indicated that amino derivatives of fused furo(thieno)[3,2-*d*]pyrimidines can show a broad spectrum of biological activity. In particular, these compounds are PI3 kinase p110a [8] and phosphodiesterase type 4 [9] inhibitors, and they possess antimicrobial [10] and antifungal [11] activities.

We have previously reported on the synthesis and biological activity of some amino derivatives of condensed furo- and thieno [3,2-*d*]pyrimidines [12,13,14,15,16]. Particularly, the studies showed that these compounds are distinguished by high anticonvulsant [12,13,14] and antibacterial activity [15,16]. Continuing our studies in the field of searching for new heterocyclic compounds of biological interest, herein we report on the synthesis and antitumor activity of new amino derivatives of pyrido[3′,2′:4,5](furo)thieno[3,2-*d*]pyrimidines as well as new heterocyclic systems: furo[2,3-*e*]imidazo[1,2-*c*]pyrimidine and furo[2,3-*e*]pyrimido[1,2-*c*]pyrimidine.

It should be mentioned that our first investigations into the amino derivatives of condensed pyrido[3′,2′:4,5]furo(thieno)[3,2-*d*]pyrimidines were reported some years ago during an international conference [17,18].

## 2. Results and Discussion

### 2.1. Chemistry

**Scheme 1:**
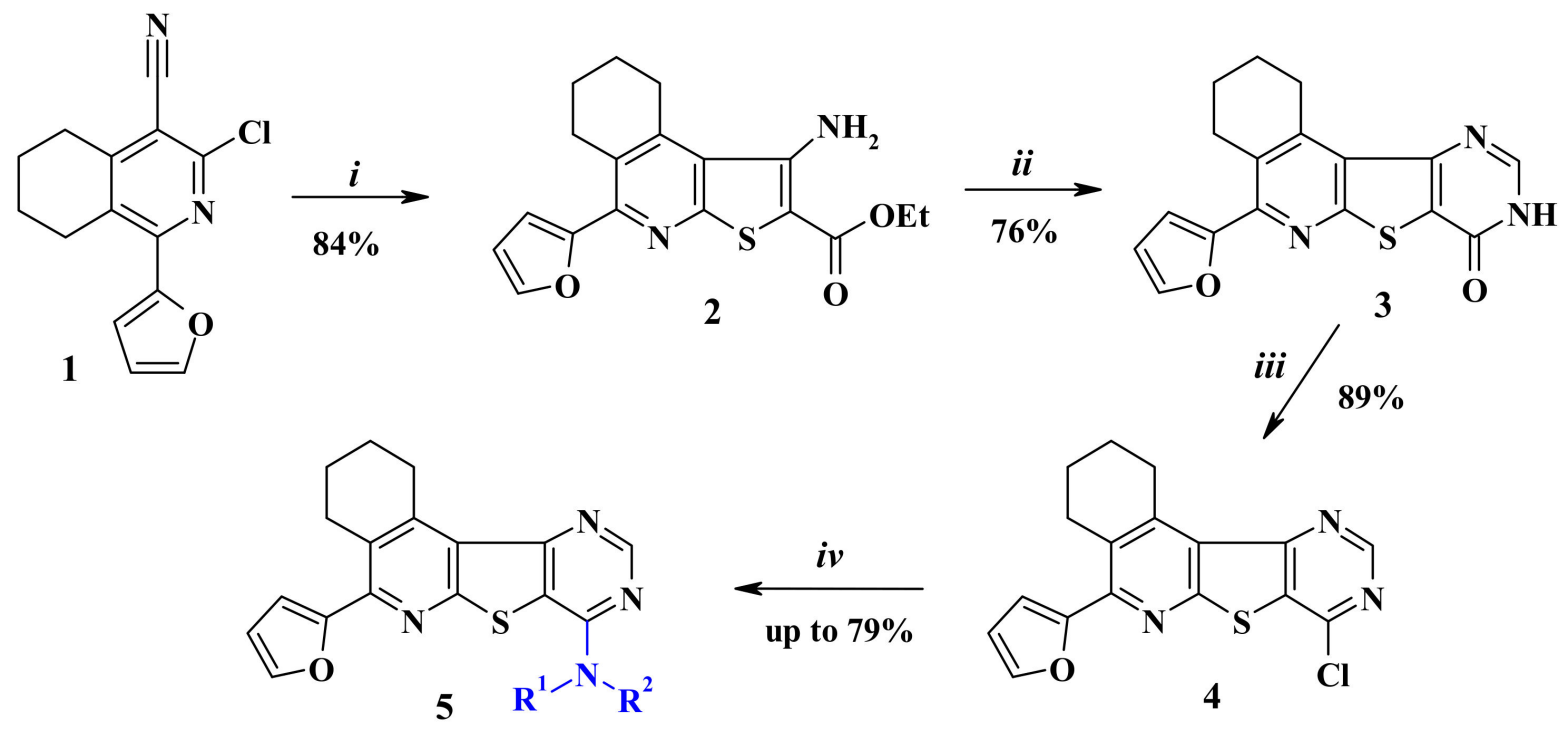
Reagents and conditions: (i) HSCH_2_COOEt, NaOEt/EtOH, reflux 5 h; (ii) HCONH_2_, reflux 4 h; (iii) POCl_3_, reflux 5 h; (iv) NHR^1^R^2^, EtOH, reflux 8 h.

Here, 3-chloro-1-(2-furyl)-5,6,7,8-tetrahydroisoquinoline-4-carbonitrile (**1**) [19] was used as a starting compound: it was treated with ethyl mercaptoacetate in the presence of sodium ethoxide, thus obtaining ethyl 1-amino-5-(2-furyl)-6,7,8,9-tetrahydrothieno[2,3-*c*]isoquinoline-2-carboxylate (**2**). The presence of two functional groups in the thiophene ring of compound **2** allowed for the cyclocondensation of the latter through the action of formamide. As a result, the corresponding 5-(2-furyl)-1,2,3,4-tetrahydropyrimido[4′,5′:4,5]thieno[2,3-*c*]isoquinolin-8(9*H*)-one (**3**) was obtained. In turn, compound **3**, through a reaction with phosphorus oxychloride, led to 8-chloro-5-(2-furyl)-1,2,3,4tetrahydropyrimido[4′,5′:4,5]thieno[2,3-*c*]isoquinoline (**4**), which contained an “activated” chlorine atom that could be easily displaced by nucleophiles. Thus, further reaction of 8-chlorothieno[3,2-*d*]pyrimidine (**4**) with various amines in ethanol led to the formation of a series of 5-(2-furyl)-1,2,3,4-tetrahydropyrimido[4′,5′:4,5]thieno[2,3-*c*]isoquinolin-8-amines (**5a–f**) in very high yields (Scheme 1, from 79% to 88%).

**Scheme 2:**
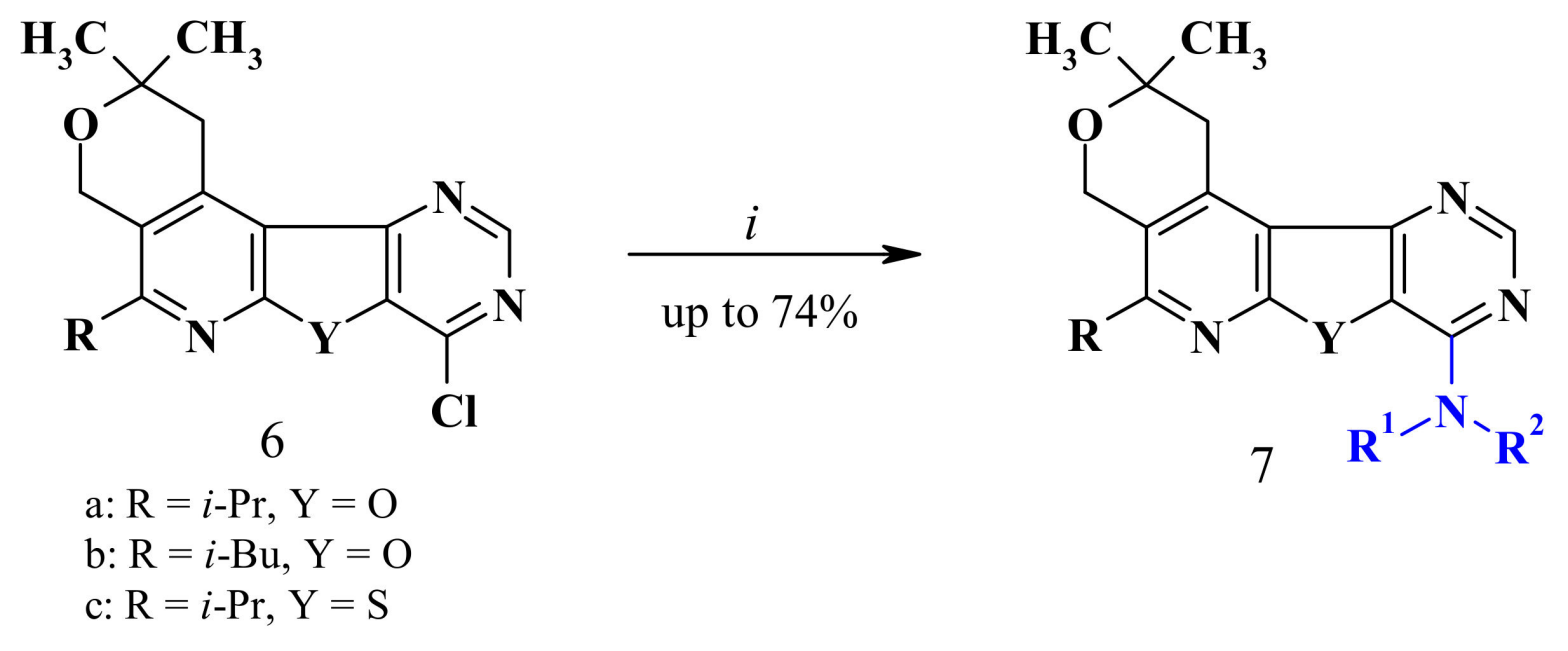
Reagents and conditions: (i) NHR^1^R^2^, EtOH, reflux 8 h.

In turn, the nucleophilic substitution of 8-chloro-2,2-dimethyl-1,4-dihydro-2*H*-pyrano[4″,3″:4′,5′]pyrido[3′,2′:4,5]furo(thieno)[3,2-*d*]pyrimidines (**6a**–**c**), the second starting compounds previously synthesized by us [13], with a series of amines gave (in very high yields, from 74% to 89%) the aimed-for pyrano[4″,3″:4′,5′]pyrido[3′,2′:4,5]furo(thieno)[3,2-*d*]pyrimidin-8-amines (**7a**–**m**) [13,20] (Scheme 2).

**Scheme 3:**
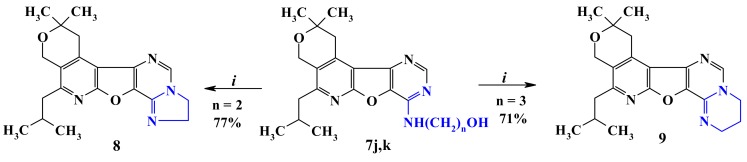
Reagents and conditions: (i) POCl_3_, reflux 10 h, then KOH.

In the following step, the new heterocyclic systems, i.e., 11-isobutyl-8,8-dimethyl2,3,7,10-tetrahydro-8H-imidazo[1,2-c]pyrano[4″,3″:4′,5′]pyrido[3′,2′:4,5]furo[2,3-e]pyrimidine (**8**) and 12-isobutyl-9,9-dimethyl3,4,8,11-tetrahydro-2H,9H-pyrano[4″,3″:4′,5′]pyrido[3′,2′:4,5]furo[2,3-e]pyrimido[1,2-c]pyrimidine (**9**), were synthesized from compounds **7j**,**k** by simple refluxing with phosphorus oxychloride followed by treatment with a base (Scheme 3). The disappearance of NH and OH groups in the ^1^H-NMR spectrum of compounds **8** and **9** supported the cyclized structures.

The copies of ^1^H-NMR and ^13^C-NMR spectra for all new synthesized compounds were submitted along with the manuscript (Supplementary Materials).

### 2.2. Biology

It is known that DNA methylation in cancer plays a variety of roles, helping to change the healthy regulation of gene expression to a disease pattern. DNA hypermethylation in neoplasia is a specific epigenetic sign that can guide the diagnosis and treatment of this disease. We studied the effect of synthesized substances on tumor DNA in vitro. After the incubation of tumor cells with the studied compounds, the isolated DNA was hydrolyzed to nitrogen bases. In the studied DNA, the nucleotide composition (guanine (G), cytosine (C), 5-methylcitosine (5-MC), adenine (A), thymine (T)) complied with the rules of Chargaff. Isolated DNA belonged to the AT type, and the amount of basic bases (G + C + 5-MC) within them was 42.24–44.64 mol %, which is typical for eukaryotic DNA. Almost the same content of basic bases (G, C, A, and T) was observed, while a clear difference between DNA samples was found only in relation to the content of 5-MC, which indicated the demethylating ability of the compounds.

A distinct difference between tumor DNA samples after treatment with compounds **5** and **7** was observed only for the 5-MC content, and the data are given in Table 1. Most of the compounds inhibited the methylation level of tumor DNA. The highest activity was found for three compounds (**5f**, **7b**, and **5b**), which reduced the 5-MC content of tumor DNA by 89.1%, 62.5%, and 60.9%, respectively. Under identical experimental conditions, doxorubicin suppressed the level of methylation of tumor DNA by 67.2%. Meanwhile, two compounds (**7i**,**j**) did not affect DNA methylation. One compound (**7a**) showed the opposite effect (the degree of methylation of tumor DNA increased). The obtained results are presented in Table 1.

DNA demethylation in the tumor tissue under the influence of compounds **5a–f** and **7b–h,k–m** can be explained by the enzymatic demethylation of 5-MC. It is known that demethylating agents may reactivate tumor suppressor genes aberrantly methylated in tumor cells, since DNA methylation is reversible, leading to the inhibition of tumor growth. For example, decitabine and 2′-deoxy-5-azacytidine can inhibit DNA methyltransferases and reverse the epigenetic silencing of aberrantly methylated genes [21,22]. 

The increase in methylation level under the influence of compound **7a** could be explained by its damaging effect on tumor DNA. It is assumed that single-stranded DNA formed during replication or repair might serve as a nucleation site for de novo methylation by DNA methyltransferase, which often occurs in tumors [23].

A study of the structure–activity relationships revealed that the activity of the tested compounds depended mostly on the nature of the amine fragment. Thus, the presence of the 2-tetrahydrofurylmethyl (**5f** and **7f**,**m**) group seemed to have a beneficial effect on antitumor activity. In the series of 5-(2-furyl)-1,2,3,4-tetrahydropyrimido[4′,5′:4,5]thieno[2,3-*c*]isoquinolin-8-amines (**5**), the replacement of the piperidine group (**5a**) with 4-methylpiperidine (**5b**) led to a very active compound. Among the pyrano[4″,3″:4′,5′]pyrido[3′,2′:4,5]furo[3,2-*d*]pyrimidin-8-amines (**7a**–**k**), the highest activity was displayed by compound **7b**, which had a CH_2_CH_2_N(Me)_2_ group. The lengthening of the alkyl chain with one more CH_2_ group (**7i**) led to a loss of activity. Compounds containing a CH_2_CH_2_OH group (**7a,j**) had no activity. Replacing the furan ring (**7f**) with a thiophene one (**7m**) also increased antitumor activity. The introduction of 2-morpholinoethyl (**7d**) as well as CH_2_CH_2_C_6_H_3_*-m*,*p*-OMe (**7e**) seemed to play a positive role in activity.

**Figure 1:**
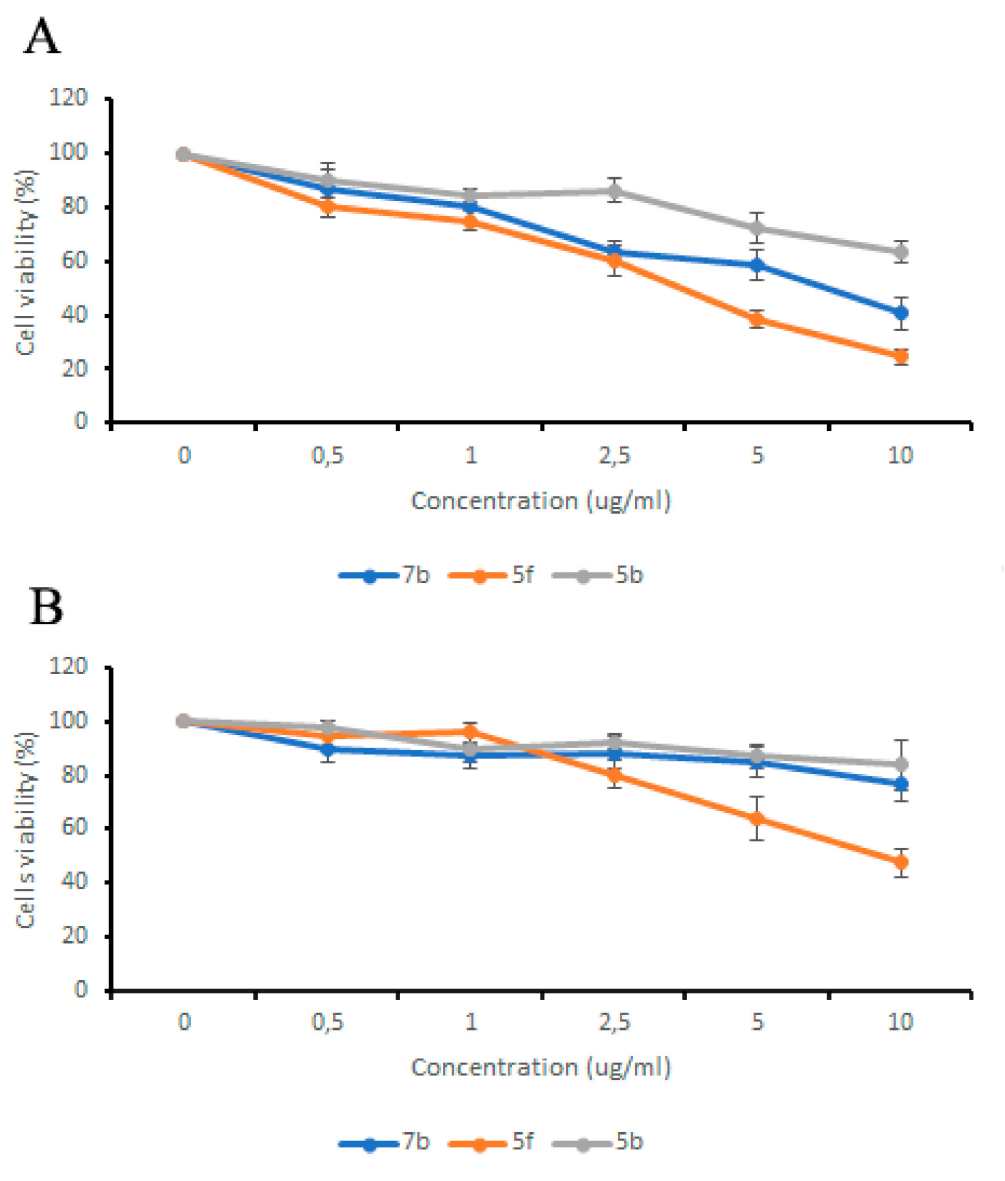
Effects of compounds **5b**, **5f**, and **7b** on the proliferation of HeLa (**A**) and Vero (**B**) cells. Cell viability was calculated through a comparison to the control, which contained <1% of DMSO (dimethyl sulfoxide). Representative data with means ± SD from three independent experiments.

To test if compounds **5b**, **5f**, and **7b** had an inhibitory effect on cell proliferation, the antiproliferative activity was assessed in both a cancer cell line (HeLa) and in normal cells (Vero). As shown in Figure 1A,B, all three compounds were active against cancer and normal cells. Compound **5f** was more potent against HeLa (IC_50_ = 4.4 ± 0.3 μg/mL) and Vero (IC_50_ = 6.4 ± 0.4 μg/mL) cells than compounds **7b** (HeLa: IC_50_ = 5.4 ± 0.2 μg/mL; Vero: 13.7 ± 0.4 μg/mL) and **5b** (HeLa: 8.4 ± 0.2 ug/mL; Vero: 16.8 ± 0.6 μg/mL) were. In general, all tested compounds were less cytotoxic against Vero, indicating low cytotoxicity toward normal cells.

### 2.3. Docking Analysis

In order to identify possible binding sites and the mechanisms of action of the active compounds, molecular docking was performed. DNA (cytosine-5)-methyltransferase 1 (DNMT1) plays a crucial role in DNA methylation [24]. With this in mind, we predicted its binding pockets using DNMT1 structures and performed a docking analysis on each of those pockets, including the sinefungin binding site, which is a well-known DNMT1 inhibitor [25]. According to our results, compounds **5b**,**f** and **7b** had the highest affinity to BAH2 (bromo-adjacent homology domain 2) of DNMT1. BAH2 consists of about 110 amino acids and folds with a tilted β-barrel, forming a cavity [26]. In DNMT1, this domain contributes to DNA binding and interacts with the main catalytic MTase domain. Moreover, BAH2 is present in a number of proteins involved in the DNA methylation process [27]. Thus, the docking showed that compounds **5b**,**f** and **7b** could bind in the cavity of the BAH2 domain with −14.73, −15.59, and −10.64 kcal/mol, respectively (Table 2). Hydrophobic interactions also contributed to the complex stabilization of binding energy. 

**Figure 2:**
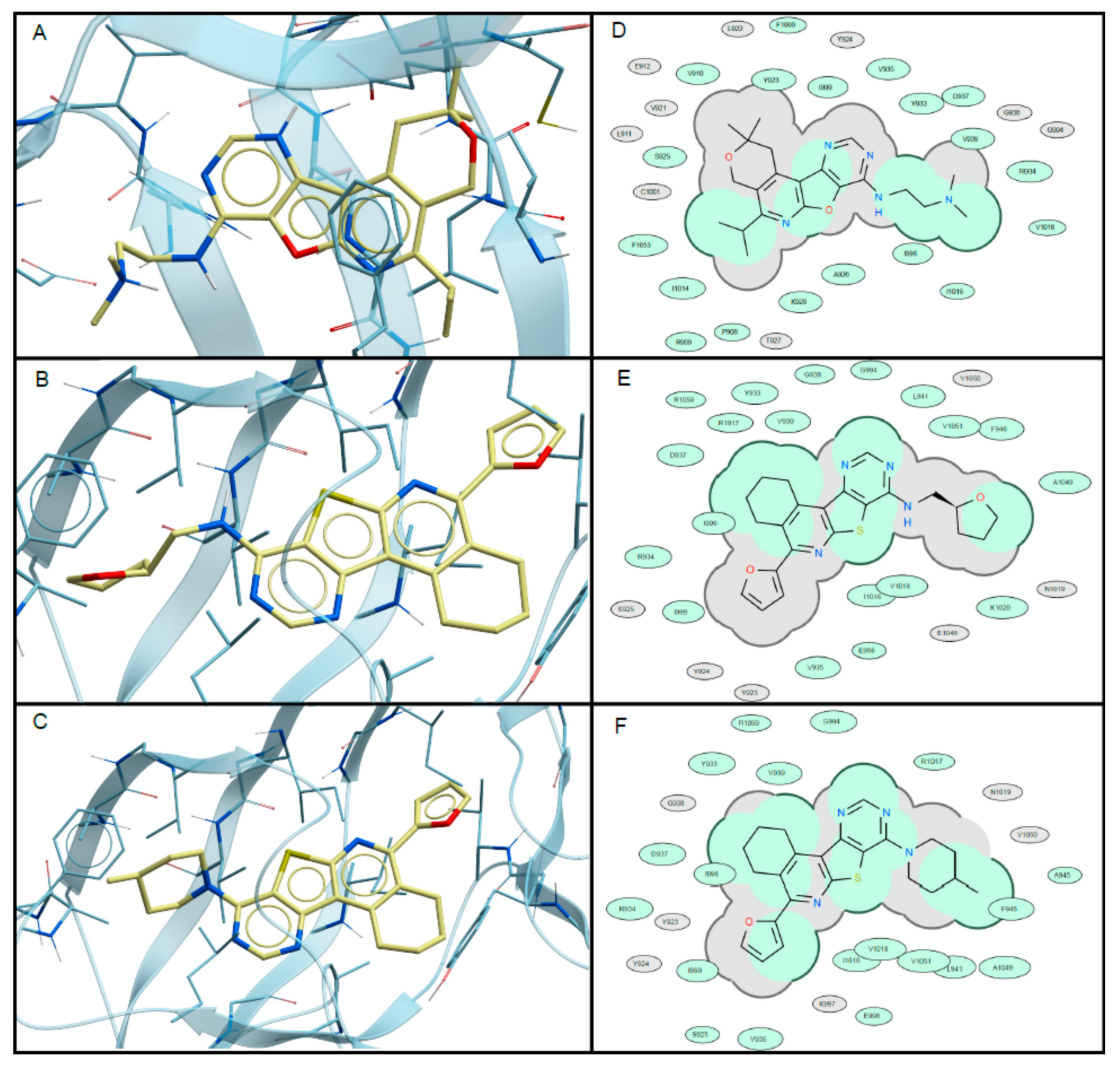
Docking positions of **7b** (**A**), **5f** (**B**), and **5b** (**C**) in the cavity of the bromo-adjacent homology domain 2 (BAH2) domain of DNA (cytosine-5)-methyltransferase 1 (DNMT1) and their 2D interaction diagrams (**D**–**F**). In the 2D diagrams, the broken thick lines around the ligand shape indicate an accessible surface, and gray and green colors represent hydrophobic and hydrophilic areas, respectively.

Compounds **5b** and **5f** had very similar binding positions (Figure 2A,D and Figure 2B,E), and they both could bind with amino acids Y933, V935, V939, L941, F946, I996, I999, V1018, A1049, and V1051 via hydrophobic interactions. However, the binding mode of **7b** was slightly different, as it was located in the side of the pocket (Figure 2C,F) and interacted mainly with amino acids I999, Y923, I996, I1014, F1053, V910, A926, V939, Y933, and K928. The estimated binding energies of the investigated compounds showed approximately the same results as the DNA methylation tests. Compound **5f** had the highest binding energy, although the other two compounds did not fit well in the in vitro experiments.

Docking with the sinefungin binding site did not show a high binding energy or Internal Coordinate Mechanics (ICM) score: this allows us to suggest that these compounds do not interact with this site and have another mechanism of action that is different from sinefungin [25]. It is known that DNMT1 can adopt several conformational states under different DNA binding conditions [26]. Probably, the interaction of the active compounds with the BAH2 domain changes its flexibility and affects its function.

#### Methods

A docking analysis was performed using ICM-Pro 3.8–7 [28]. To imitate protein flexibility, we used a 4D docking method with several crystallographic structures of DNMT1 (PDB: 3SWR, 4WXX, 4YOC, 3AV6, 3PT6, 4DA4, and 5GUV) [29]. Protein structures were minimized, and hydrogens were added: 3D structures for **5b**,**f** and **7b** were built, adding hydrogens, assigned charges, and relaxed geometry. The docking was performed by simulating ligand flexibility and with docking effort 10. Binding pockets were identified using icmPocketFinder [30]. We performed the docking analysis 10 times for each identified binding site and evaluated the results using a standard ICM score and the estimated binding energy [31]. 

## 3. Materials and Methods 

### 3.1. General Information

^1^H- and ^13^C-NMR spectra were recorded on a Varian Mercury 300VX spectrometer in DMSO (dimethyl sulfoxide)/CCl_4_ (1/3) solution (300 MHz for ^1^H and 75 MHz for ^13^C, respectively). Chemical shifts were reported as δ (parts per million) relative to TMS (tetramethylsilane) as an internal standard. IR spectra were recorded on a Nicolet Avatar 330-FT-IR spectrophotometer, and the reported wave numbers are given in cm^−1^. Elemental analyses were performed on an Elemental Analyzer Euro EA 3000. Melting points were determined on a Boetius microheating stage. Compounds **1 [19]**, **6 [13]**, and **7a**,**b**,**d**,**h**,**i**,**k** [13,20] have already been described.

Ethyl 1-amino-5-(2-furyl)-6,7,8,9-tetrahydrothieno[2,3-c]isoquinoline-2-carboxylate (**2**): To a solution of sodium ethoxide (0.28 g (12 mmol) of sodium in absolute ethanol (30 mL)), ethyl 2-mercaptoacetate (1.44 g, 12 mmol) and compound **1** (2.59 g, 10 mmol) were added. The mixture was refluxed for 5 h, cooled, and poured onto ice. The formed crystals were filtered off, washed with water, dried, and recrystallized from ethanol. Light-yellow solid, yield 84%, m.p. 192–194 °C; IR *ν*/cm^−1^: 3485, 3346 (NH_2_), 1664 (C=O). ^1^H-NMR (300 MHz, DMSO/CCl_4_, 1/3) δ 7.64 (dd, *J* = 1.8, 0.8 Hz, 1H, 5-CH_furyl_), 7.03 (dd, *J* = 3.5, 0.8 Hz, 1H, 3-CH_furyl_), 6.63 (br, 2H, NH_2_), 6.55 (dd, *J* =3.5, 1.8 Hz, 1H, 4-CH_furyl_), 4.30 (q, *J* = 7.1 Hz, 2H, CH_2_CH_3_), 3.38–3.32 (m, 2H, 9-CH_2_), 3.07–3.01 (m, 2H, 6-CH_2_), 1.94–1.78 (m, 4H, 7,8-CH_2_), 1.39 (t, *J* = 7.1 Hz, 3H, CH_2_CH_3_). ^13^C NMR (75 MHz, DMSO/CCl_4_, 1/3) δ 164.5, 157.5, 153.3, 149.7, 147.9, 144.5, 142.8, 124.7, 122.4, 112.2, 110.9, 59.2, 26.7, 26.6, 21.6, 21.1, 14.1. Anal. calcd (Analytically calculated) for C_18_H_18_N_2_O_3_S: C 63.14; H 5.30; N 8.18%. Found: C 63.47; H 5.49; N 8.42%.

5-(2-Furyl)-1,2,3,4-tetrahydropyrimido[4′,5′:4,5]thieno[2,3-c]isoquinolin-8(9H)-one (**3**): A mixture of compound **2** (3.42 g, 10 mmol) and formamide (30 mL) was refluxed for 5 h. After cooling, the separated crystals were filtered off, washed with water, dried, and recrystallized from dimethylformamide. Gray solid, yield 76%, m.p. > 360 °C; IR *ν*/cm^−1^: 3108 (NH), 1642 (C=O). ^1^H-NMR (300 MHz, DMSO/CCl_4_, 1/3) δ 12.88 (br s, 1H, NH), 8.33 (s, 1H, 10-CH), 7.94 (dd, *J* = 1.8, 0.8 Hz, 1H, 5-CH_furyl_), 7.17 (dd, *J* = 3.5, 0.8 Hz, 1H, 3-CH_furyl_), 6.72 (dd, *J* = 3.5, 1.8 Hz, 1H, 4-CH_furyl_), 3.59–3.53 (m, 2H, 1-CH_2_), 3.06–2.99 (m, 2H, 4-CH_2_), 1.88–1.80 (m, 4H, 2,3-CH_2_). Anal. calcd for C_17_H_13_N_3_O_2_S: C 63.14; H 4.05; N 12.99%. Found: C 63.52; H 4.28; N 13.25%.

8-Chloro-5-(2-furyl)-1,2,3,4-tetrahydropyrimido[4′,5′:4,5]thieno[2,3-c]isoquinoline (**4**). A mixture of compound **3** (3.23 g, 10 mmol) and phosphorus oxychloride (30 mL) was refluxed for 4 h. The excess phosphorus oxychloride was distilled off to give a dry residue. Ice water and then ammonia solution were added. The separated crystals were filtered off, washed with water, dried, and recrystallized from a mixture of ethanol–chloroform (1:2). Light-yellow solid, yield 89%, m.p. 206–208 °C. ^1^H-NMR (300 MHz, DMSO/CCl_4_, 1/3) δ 8.98 (s, 1H, 10-CH), 7.73 (dd, *J* = 1.8, 0.8 Hz, 1H, 5-CH_furyl_), 7.20 (dd, *J* = 3.5, 0.8 Hz, 1H, 3-CH_furyl_), 6.62 (dd, *J* = 3.5, 1.8 Hz, 1H, 4-CH_furyl_), 3.64–3.59 (m, 2H, 1-CH_2_), 3.17–3.11 (m, 2H, 4-CH_2_), 2.00–1.86 (m, 4H, 2,3-CH_2_). ^13^C NMR (75 MHz, DMSO/CCl_4_, 1/3) δ 158.9, 157.5, 153.3, 153.2, 152.8, 149.4, 147.9, 143.9, 129.6, 126.7, 122.3, 113.9, 111.4, 27.5, 26.6, 21.8, 20.7. Anal. calcd for C_17_H_12_ClN_3_OS: C 59.73; H 3.54; N 12.29%. Found: C 60.09; H 3.76; N 12.54%.

### 3.2. General Method for the Preparation of Compounds 5a–f and 7c,e–g,j,l,m

A mixture of compound **4** or **6** (5 mmol) and the corresponding amine (11 mmol) in absolute ethanol (30 mL) was refluxed for 8 h. The ethanol was distilled off to dryness, and water (50 mL) was added to the residue. The separated crystals were filtered off, washed with water, dried, and recrystallized from ethanol.

*5-(2-Furyl)-8-piperidin-1-yl-1,2,3,4-tetrahydropyrimido[4′,5′:4,5]thieno[2,3-c]isoquinoline* (**5a**): Cream solid, yield 84%, m.p. 167‒169 °C. ^1^H-NMR (300 MHz, DMSO/CCl_4_, 1/3) δ 8.52 (s, 1H, 10-CH), 7.68 (dd, *J* = 1.7, 0.7 Hz, 1H, 5-CH_furyl_), 7.07 (dd, *J* = 3.5, 0.7 Hz, 1H, 3-CH_furyl_), 6.58 (dd, *J* = 3.5, 1.7 Hz, 1H, 4-CH_furyl_), 3.95–3.89 (m, 4H, N(CH_2_)_2_), 3.71–3.65 (m, 2H, 1-CH_2_), 3.16–3.10 (m, 2H, 4-CH_2_), 1.97–1.84 (m, 4H, 2,3-CH_2_), 1.82–1.69 (m, 6H, 3CH_2_, C_5_H_10_N). ^13^C NMR (75 MHz, DMSO/CCl_4_, 1/3) δ 157.4, 157.1, 156.5, 153.4, 153.0, 148.2, 146.6, 143.0, 125.9, 122.9, 112.8, 112.4, 111.1, 46.8, 27.4, 26.7, 25.5, 24.2, 21.9, 21.0. Anal. calcd for C_22_H_22_N_4_OS: C 67.67; H 5.68; N 14.35%. Found: C 68.01; H 5.88; N 14.58%.

*5-(2-Furyl)-8-(4-methylpiperidin-1-yl)-1,2,3,4-tetrahydropyrimido[4′,5′:4,5]thieno[2,3-c]isoquinoline* (**5b**): Light-yellow solid, yield 79%, m.p. 110–112 °C. ^1^H-NMR (300 MHz, DMSO/CCl_4_, 1/3) δ 8.51 (s, 1H, 10-CH), 7.68 (dd, *J* = 1.8, 0.7 Hz, 1H, 5-CH_furyl_), 7.07 (dd, *J* = 3.4, 0.7 Hz, 1H, 3-CH_furyl_), 6.59 (dd, *J* = 3.4, 1.8 Hz, 1H, 4-CH_furyl_), 4.77–4.68 (m, 2H, NCH_2_), 3.71–3.64 (m, 2H, 1-CH_2_), 3.16–3.05 (m, 4H, NCH_2_, 4-CH_2_), 1.99–1.69 and 1.38–1.22 (both m, 7H and 2H, C_5_H_9_N, 2,3-CH_2_), 1.01 (d, *J* = 6.2 Hz, 3H, CH_3_). ^13^C NMR (75 MHz, DMSO/CCl_4_, 1/3) δ 157.4, 157.1, 156.5, 153.4, 153.1, 148.2, 146.6, 143.0, 125.9, 122.9, 112.8, 112.4, 111.1, 46.0, 33.7, 30.7, 27.5, 26.7, 21.9, 21.4, 21.0. Anal. calcd for C_23_H_24_N_4_OS: C 68.29; H 5.98; N 13.85%. Found: C 68.61; H 6.15; N 14.07%.

*5-(2-Furyl)-8-morpholin-4-yl-1,2,3,4-tetrahydropyrimido[4′,5′:4,5]thieno[2,3-c]isoquinoline* (**5c**): Cream solid, yield 88%, m.p. 222‒224 °C. ^1^H-NMR (300 MHz, DMSO/CCl_4_, 1/3) δ 8.58 (s, 1H, 10-CH), 7.70 (dd, *J* = 1.7, 0.8 Hz, 1H, 5-CH_furyl_), 7.10 (dd, *J* = 3.4, 0.8 Hz, 1H, 3-CH_furyl_), 6.59 (dd, *J* = 3.4, 1.7 Hz, 1H, 4-CH_furyl_), 3.96–3.91 (m, 4H, O(CH_2_)_2_), 3.83–3.78 (m, 4H, N(CH_2_)_2_), 3.73–3.66 (m, 2H, 1-CH_2_), 3.18–3.11 (m, 2H, 4-CH_2_), 1.99–1.86 (m, 4H, 2,3-CH_2_). ^13^C NMR (75 MHz, DMSO/CCl_4_, 1/3) δ 157.6, 157.5, 156.8, 153.3, 152.9, 148.4, 146.8, 143.2, 126.2, 122.7, 113.2, 112.6, 111.1, 65.8, 46.0, 27.5, 26.7, 21.9, 20.9. Anal. calcd for C_21_H_20_N_4_O_2_S: C 64.27; H 5.14; N 14.28%. Found: C 64.64; H 5.37; N 14.54%.

*5-(2-Furyl)-N-isobutyl-1,2,3,4-tetrahydropyrimido[4′,5′:4,5]thieno[2,3-c]isoquinolin-8-amine* (**5d**): Colorless solid, yield 83%, m.p. 96–98 °C; IR *ν*/cm^−1^: 3400, 3241 (NH). ^1^H-NMR (300 MHz, DMSO/CCl_4_, 1/3) δ 8.45 (s, 1H, 10-CH), 7.68 (dd, *J* = 1.7, 0.7 Hz, 1H, 5-CH_furyl_), 7.41 (t, *J* = 5.7 Hz, 1H, NH), 7.06 (dd, *J* = 3.4, 0.7 Hz, 1H, 3-CH_furyl_), 6.58 (dd, *J* = 3.4, 1.7 Hz, 1H, 4-CH_furyl_), 3.72–3.66 (m, 2H, 1-CH_2_), 3.39–3.33 (m, 2H, NHCH_2_), 3.14–3.08 (m, 2H, 4-CH_2_), 2.06 (sp, *J* = 6.7 Hz, 1H, CH(CH_3_)_2_), 1.98–1.85 (m, 4H, 2,3-CH_2_), 0.98 (sp, *J* = 6.7 Hz, 6H, CH(CH_3_)_2_). ^13^C NMR (75 MHz, DMSO/CCl_4_, 1/3) δ 157.9, 156.7, 154.1, 153.8, 153.2, 147.7, 146.3, 142.8, 125.8, 123.9, 113.5, 112.2, 111.0, 47.7, 27.4, 27.2, 26.7, 22.1, 21.0, 20.0, 19.9. Anal. calcd for C_21_H_22_N_4_OS: C 66.64; H 5.86; N 14.80%. Found: C 66.99; H 6.07; N 15.04%.

*N,N-Diethyl-N′-[5-(2-furyl)-1,2,3,4-tetrahydropyrimido[4′,5′:4,5]thieno[2,3-c]isoquinolin-8-yl]ethane-1,2-diamine* (**5e**): Colorless solid, yield 81%, m.p. 137‒139 °C; IR *ν*/cm^−1^: 3365 (NH). ^1^H-NMR (300 MHz, DMSO/CCl_4_, 1/3) δ 8.49 (s, 1H, 10-CH), 7.68 (dd, *J* = 1.8, 0.8 Hz, 1H, 5-CH_furyl_), 7.09 (br, 1H, NH), 7.07 (dd, *J* = 3.4, 0.8 Hz, 1H, 3-CH_furyl_), 6.59 (dd, *J* = 3.4, 1.8 Hz, 1H, 4-CH_furyl_), 3.73–3.67 (m, 2H, 1-CH_2_), 3.65–3.56 (m, 2H, NHCH_2_), 3.16–3.10 (m, 2H, 4-CH_2_), 2.70 (br t, *J* = 7.2 Hz, 2H, NHCH_2_CH_2_), 2.59 (q, *J* = 7.1 Hz, 4H, N(CH_2_CH_3_)_2_), 1.99–1.86 (m, 4H, 2,3-CH_2_), 1.06 (t, *J* = 7.1 Hz, 6H, N(CH_2_CH_3_)_2_). ^13^C NMR (75 MHz, DMSO/CCl_4_, 1/3) δ 157.8, 156.4, 154.1, 153.9, 153.2, 147.7, 146.3, 142.9, 125.8, 123.9, 113.5, 112.2, 111.0, 51.1, 46.7, 38.2, 27.2, 26.7, 22.0, 21.0, 11.8. Anal. calcd for C_23_H_27_N_5_OS: C 65.53; H 6.46; N 16.61%. Found: C 65.91; H 6.66; N 16.88%.

*5-(2-Furyl)-N-(tetrahydrofuran-2-ylmethyl)-1,2,3,4-tetrahydropyrimido[4′,5′:4,5]thieno[2,3-c]isoquinolin-8-amine* (**5f**): Colorless solid, yield 87%, m.p. 146–148 °C; IR *ν*/cm^−1^: 3318 (NH). ^1^H-NMR (300 MHz, DMSO/CCl_4_, 1/3) δ 8.48 (s, 1H, 10-CH), 7.69 (dd, *J* = 1.7, 0.7 Hz, 1H, 5-CH_furyl_), 7.43 (t, *J* = 5.6 Hz, 1H, NH), 7.08 (dd, *J* = 3.4, 0.7 Hz, 1H, 3-CH_furyl_), 6.59 (dd, *J* = 3.4, 1.7 Hz, 1H, 4-CH_furyl_), 4.20–4.10 (m, 1H, OCH_furyl_), 3.91–3.83 (m, 1H, OCH_2_), 3.74–3.52 (m, 5H, NHCH_2_, OCH_2_, 1-CH_2_), 3.16–3.09 (m, 2H, 4-CH_2_), 2.06–1.62 (m, 8H, 2,3-CH_2_, 3,4-CH_2_-furyl). ^13^C NMR (75 MHz, DMSO/CCl_4_, 1/3) δ 157.9, 156.6, 154.2, 153.7, 153.2, 147.8, 146.3, 142.9, 125.8, 123.8, 113.6, 112.3, 111.1, 76.7, 66.8, 44.1, 28.5, 27.2, 26.7, 25.0, 22.1, 21.0. Anal. calcd for C_22_H_22_N_4_O_2_S: C 65.00; H 5.46; N 13.78%. Found: C 65.34; H 5.64; N 14.01%.

*N,N-Diethyl-N′-(5-isopropyl-2,2-dimethyl-1,4-dihydro-2H-pyrano[4″,3″:4′,5′]pyrido[3′,2′:4,5]furo[3,2-d]pyrimidin-8-yl)ethane-1,2-diamine* (**7c**): Colorless solid, yield 85%, m.p. 146‒148 °C; IR ν/cm^−1^: 3230 (NH). ^1^H-NMR (300 MHz, DMSO/CCl_4_, 1/3) δ 8.34 (s, 1H, 10-CH), 7.41 (br, 1H, NH), 4.86 (s, 2H, OCH_2_), 3.66–3.57 (m, 2H, NHCH_2_), 3.32 (s, 2H, 1-CH_2_), 3.05 (sp, *J* = 6.6 Hz, 1H, CH(CH_3_)_2_), 2.75–2.65 (m, 2H, CH_2_N(CH_2_)_2_), 2.64–2.54 (m, 4H, N(CH_2_)_2_), 1.35 (s, 6H, C(CH_3_)_2_), 1.29 (d, *J* = 6.6 Hz, 6H, CH(CH_3_)_2_), 1.05 (d, *J* = 7.1 Hz, 6H, N(CH_2_CH_3_)_2_). ^13^C NMR (75 MHz, DMSO/CCl_4_, 1/3) δ 161.4, 160.6, 152.9, 148.5, 140.5, 132.9, 121.8, 110.5, 68.6, 59.6, 51.2, 46.6, 37.8, 36.8, 30.3, 25.9, 21.3, 11.7. Anal. calcd for C_23_H_33_N_5_O_2_: C 67.12; H 8.08; N 17.02%. Found: C 67.48; H 8.29; N 17.27%.

*N-[2-(3,4-Dimethoxyphenyl)ethyl]-5-isopropyl-2,2-dimethyl-1,4-dihydro-2H-pyrano[4″,3″:4′,5′]pyrido[3′,2′:4,5]furo[3,2-d]pyrimidin-8-amine* (**7e**): Colorless solid, yield 82%, m.p. 207‒209 °C; IR ν/cm^−1^: 3227 (NH). ^1^H-NMR (300 MHz, DMSO/CCl_4_, 1/3) δ 8.36 (s, 1H, 10-CH), 7.85 (br, 1H, NH), 6.82–6.75 (m, 3H, C_6_H_3_), 4.87 (s, 2H, OCH_2_), 3.83–3.72 (m, 2H, NHCH_2_), 3.79 (s, 3H, OCH_3_), 3.75 (s, 3H, OCH_3_), 3.33 (s, 2H, 1-CH_2_), 3.05 (sp, *J* = 6.6 Hz, 1H, CH(CH_3_)_2_), 2.93–2.87 (m, 2H, CH_2_C_6_H_3_), 1.35 (s, 6H, C(CH_3_)_2_), 1.29 (d, *J* = 6.6 Hz, 6H, CH(CH_3_)_2_). ^13^C NMR (75 MHz, DMSO/CCl_4_, 1/3) δ 161.4, 160.6, 152.9, 148.7, 148.5, 147.2, 140.5, 132.9, 131.7, 121.8, 120.3, 112.6, 111.8, 110.5, 68.6, 59.6, 55.2, 55.1, 41.4, 36.8, 34.6, 30.3, 25.9, 21.3. Anal. calcd for C_27_H_32_N_4_O_4_: C 68.05; H 6.77; N 11.76%. Found: C 68.38; H 6.96; N 11.98%.

*5-Isopropyl-2,2-dimethyl-N-(tetrahydrofuran-2-ylmethyl)-1,4-dihydro-2H-pyrano[4″,3″:4′,5′]pyrido[3′,2′:4,5]furo[3,2-d]pyrimidin-8-amine* (**7f**): Colorless solid, yield 76%, m.p. 164‒166 °C; IR ν/cm^−1^: 3334, 3277 (NH). ^1^H-NMR (300 MHz, DMSO/CCl_4_, 1/3) δ 8.33 (s, 1H, 10-CH), 7.62 (br t, *J* = 5.8 Hz, 1H, NH), 4.86 (s, 2H, OCH_2_), 4.16–4.07 (m, 1H, OCH_furyl_), 3.90–3.82 (m, 1H, OCH_2furyl_), 3.72–3.59 (m, 3H, NHCH_2_, OCH_2furyl_), 3.32 (s, 2H, 1-CH_2_), 3.05 (sp, *J* = 6.6 Hz, 1H, CH(CH_3_)_2_), 2.04–1.64 (m, 4H, 3,4-CH_2furyl_), 1.34 (s, 6H, C(CH_3_)_2_), 1.29 (d, *J* = 6.6 Hz, 6H, CH(CH_3_)_2_). ^13^C NMR (75 MHz, DMSO/CCl_4_, 1/3) δ 161.5, 160.6, 152.8, 148.6, 140.5, 132.8, 121.8, 110.5, 76.6, 68.6, 66.9, 59.6, 43.6, 36.8, 30.3, 28.4, 25.9, 25.0, 21.3. Anal. calcd for C_22_H_28_N_4_O_3_: C 66.64; H 7.12; N 14.13%. Found: C 67.01; H 7.34; N 14.39%.

*N-(2-Furylmethyl)-5-isopropyl-2,2-dimethyl-1,4-dihydro-2H-pyrano[4″,3″:4′,5′]pyrido[3′,2′:4,5]furo[3,2-d]pyrimidin-8-amine* (**7g**): Colorless solid, yield 88%, m.p. 180‒182 °C; IR *ν*/cm^−1^: 3286, 3217 (NH). ^1^H-NMR (300 MHz, DMSO/CCl_4_, 1/3) δ 8.38 (s, 1H, 10-CH), 8.26 (t, *J* = 6.0 Hz, 1H, NH), 7.37 (dd, *J* = 1.9, 1.0 Hz, 1H, 5-CH_furyl_), 6.29 (dd, *J* = 3.3, 1.9 Hz, 1H, 3-CH_furyl_), 6.25 (dd, *J* = 3.3, 1.0 Hz, 1H, 4-CH_furyl_), 4.88 (s, 2H, OCH_2_), 4.76 (d, *J* = 6.0, 2H, NHCH_2_), 3.32 (s, 2H, 1-CH_2_), 3.05 (sp, *J* = 6.6 Hz, 1H, CH(CH_3_)_2_), 1.35 (s, 6H, C(CH_3_)_2_), 1.29 (d, *J* = 6.6 Hz, 6H, CH(CH_3_)_2_). ^13^C NMR (75 MHz, DMSO/CCl_4_, 1/3) δ 161.6, 160.7, 152.8, 152.2, 148.1, 144.2, 140.8, 140.6, 132.9, 121.8, 110.4, 109.8, 106.4, 68.6, 59.6, 36.8, 36.4, 30.3, 25.8, 21.3. Anal. calcd for C_22_H_24_N_4_O_3_: C 67.33; H 6.16; N 14.28%. Found: C 67.64; H 6.36; N 14.52%.

*2-[(5-Isobutyl-2,2-dimethyl-1,4-dihydro-2H-pyrano[4″,3″:4′,5′]pyrido[3′,2′:4,5]furo[3,2-d]pyrimidin-8-yl)amino]ethanol* (**7j**): Colorless solid, yield 74%, m.p. 219‒220 °C; IR *ν*/cm^−1^: 3226 (NH). ^1^H-NMR (300 MHz, DMSO/CCl_4_, 1/3) δ 8.33 (s, 1H, 10-CH), 7.54 (br, 1H, NH), 4.79 (s, 2H, OCH_2_), 4.45 (br t, *J* = 5.4 Hz, 1H, OH), 3.67–3.62 (m, 4H, NHCH_2_CH_2_), 3.31 (s, 2H, 1-CH_2_), 2.56 (d, *J* = 7.1 Hz, 2H, CHCH_2_), 2.33–2.19 (m, 1H, CH(CH_3_)_2_), 1.33 (s, 6H, C(CH_3_)_2_), 0.98 (d, *J* = 6.8 Hz, 6H, CH(CH_3_)_2_). ^13^C NMR (75 MHz, DMSO/CCl_4_, 1/3) δ 160.3, 156.1, 152.9, 148.7, 143.8, 140.3, 132.9, 123.4, 110.4, 68.7, 60.0, 59.8, 42.8, 42.0, 36.7, 27.3, 25.9, 22.2. Anal. calcd for C_20_H_26_N_4_O_3_: C 64.84; H 7.07; N 15.12%. Found: C 65.19; H 7.25; N 15.35%.

*Ethyl 4-(5-isopropyl-2,2-dimethyl-1,4-dihydro-2H-pyrano[4″,3″:4′,5′]pyrido[3′,2′:4,5]thieno[3,2-d]pyrimidin-8-yl)piperazine-1-carboxylate* (**7l**): Colorless solid, yield 84%, m.p. 217‒219 °C; IR ν/cm^−1^: 1711 (C=O). ^1^H-NMR (300 MHz, DMSO/CCl_4_, 1/3) δ 8.60 (s, 1H, 10-CH), 4.88 (s, 2H, OCH_2_), 4.12 (q, *J* = 7.1 Hz, 2H, CH_2_CH_3_), 4.00–3.94 (m, 4H, N(CH_2_)_2_), 3.64–3.59 (m, 4H, N(CH_2_)_2_), 3.56 (s, 2H, 1-CH_2_), 3.08 (sp, *J* = 6.6 Hz, 1H, CH(CH_3_)_2_), 1.35 (s, 6H, C(CH_3_)_2_), 1.31 (d, *J* = 6.6 Hz, 6H, CH(CH_3_)_2_), 1.29 (t, *J* = 7.1 Hz, 3H, CH_2_CH_3_). ^13^C NMR (75 MHz, DMSO/CCl_4_, 1/3) δ 163.6, 158.7, 157.4, 156.7, 154.0, 153.1, 141.6, 122.9, 122.1, 112.7, 68.6, 60.5, 59.6, 45.3, 43.0, 42.9, 36.9, 30.4, 26.0, 21.2, 14.2. Anal. calcd for C_24_H_31_N_5_O_3_S: C 61.38; H 6.65; N 14.91%. Found: C 61.71; H 6.82; N 15.16%.

*5-Isopropyl-2,2-dimethyl-N-(tetrahydrofuran-2-ylmethyl)-1,4-dihydro-2H-pyrano[4″,3″:4′,5′]pyrido[3′,2′:4,5]thieno[3,2-d]pyrimidin-8-amine* (**7m**): Colorless solid, yield 89%, m.p. 179‒181 °C; IR ν/cm^−1^: 3299 (NH). ^1^H-NMR (300 MHz, DMSO/CCl_4_, 1/3) δ ^1^H NMR (300 MHz, DMSO/CCl_4_, 1/3) δ 8.83 (s, 1H, 10-CH), 7.80 (t, *J* = 5.6 Hz, 1H, NH), 5.23 (s, 2H, OCH_2_), 4.56–4.47 (m, 1H, OCH_furyl_), 4.27–4.19 (m, 1H, OCH_2_), 4.10–3.92 (m, 3H, NHCH_2_, OCH_2_), 3.91 (s, 2H, 1-CH_2_), 3.43 (sp, *J* = 6.6 Hz, 1H, CH(CH_3_)_2_), 2.42–1.98 (m, 4H, 3,4-CH_2_-furyl), 1.70 (s, 6H, C(CH_3_)_2_), 1.69 (d, *J* = 6.6 Hz, 6H, CH(CH_3_)_2_). ^13^C NMR (75 MHz, DMSO/CCl_4_, 1/3) δ 162.5, 159.1, 156.6, 154.1, 153.7, 141.1, 122.9, 122.3, 112.9, 76.7, 68.6, 66.8, 59.6, 44.1, 36.7, 30.3, 28.5, 26.1, 25.0, 21.3. Anal. calcd for C_22_H_28_N_4_O_2_S: C 64.05; H 6.84; N 13.58%. Found: C 64.41; H 7.04; N 13.81%.

### 3.3. General Method for the Preparation of Compounds **8** and **9**

A mixture of compound **7j** or **7k** (5 mmol) and phosphorus oxychloride (30 mL) was refluxed for 4 h. The excess of phosphorus oxychloride was distilled off to give a dry residue. Ice water was added, and then the mixture was treated with potassium hydroxide solution (pH = 8–9). The separated crystals were filtered off, washed with water, dried, and recrystallized from ethanol.

*11-Isobutyl-8,8-dimethyl-2,3,7,10-tetrahydro-8H-imidazo[1,2-c]pyrano[4″,3″:4′,5′]pyrido[3′,2′:4,5]furo[2,3-e]pyrimidine* (**8**): Light-yellow solid, yield 77%, m.p. 270‒272 °C. ^1^H-NMR (300 MHz, DMSO/CCl_4_, 1/3) δ 8.00 (s, 1H, 5-CH), 4.77 (s, 2H, OCH_2_), 4.27–4.18 (m, 2H, 2-CH_2_), 4.09–4.00 (m, 2H, 3-CH_2_), 3.20 (s, 2H, CH_2_), 2.54 (d, *J =* 7.1 Hz, 2H, CHCH_2_), 2.34–2.19 (m, 1H, CH(CH_3_)_2_), 1.31 (s, 6H, C(CH_3_)_2_), 0.98 (d, 6H, *J =* 6.6 Hz, CH(CH_3_)_2_). ^13^C NMR (75 MHz, DMSO/CCl_4_, 1/3) δ 160.1, 155.1, 145.5, 144.7, 139.1, 137.9, 134.0, 123.6, 110.5, 68.6, 60.0, 53.2, 46.3, 41.9, 36.5, 27.4, 25.8, 22.2. Anal. calcd for C_20_H_24_N_4_O_2_: C 68.16; H 6.86; N 15.90%. Found: C 68.53; H 7.05; N 16.14%.

*12-Isobutyl-9,9-dimethyl-3,4,8,11-tetrahydro-2H,9H-pyrano[4″,3″:4′,5′]pyrido[3′,2′:4,5]furo[2,3-e]pyrimido[1,2-c]pyrimidine* (**9**): Colorless solid, yield 71%, m.p. 282–284 °C. ^1^H-NMR (300 MHz, DMSO/CCl_4_, 1/3) δ 7.60 (s, 1H, 6-CH), 4.76 (s, 2H, OCH_2_), 4.07–4.01 (m, 2H, 2-CH_2_), 3.56 (t, *J =* 5.6 Hz, 2H, 4-CH_2_), 3.18 (s, 2H, CH_2_), 2.53 (d, *J =* 7.1 Hz, 2H, CHCH_2_), 2.34–2.19 (m, 1H, CH(CH_3_)_2_), 2.06–1.96 (m, 2H, 3-CH_2_), 1.31 (s, 6H, C(CH_3_)_2_), 0.98 (d, 6H, *J =* 6.6 Hz, CH(CH_3_)_2_). ^13^C NMR (75 MHz, DMSO/CCl_4_, 1/3) δ 149.6, 144.1, 137.1, 128.4, 128.1, 127.8, 123.9, 113.2, 100.9, 58.6, 50.0, 35.9, 33.2, 31.9, 26.4, 17.4, 15.8, 12.1, 10.0. Anal. calcd for C_21_H_26_N_4_O_2_: C 68.83; H 7.15; N 15.29%. Found: C 69.18; H 7.36; N 15.56%.

### 3.4. Cell Viability Assay

Vero and HeLa cells were seeded in a 96-well culture plate at a density of 20,000 cells/well. The plates were preincubated in a 5% CO_2_/95% air-humidified atmosphere at 37 °C for 24 h to allow for adaptation of the cells prior to the addition of the test compounds. All compounds were dissolved in dimethyl sulfoxide (DMSO) prior to dilution (1 mg/mL stock solution). The 50% inhibitory concentration (IC_50_) was determined over a range of concentrations (0.5 μg/mL to 10 μg/mL). All cells were incubated for 72 h, and then cell viability was estimated using an MTT (3-(4,5-dimethylthiazol-2-yl)-2,5-diphenyltetrazolium bromide) assay, as previously described [32]. Colorimetric measurements were performed on a microplate reader at 570 nm (Tecan Spectra II, Switzerland). The percentage of viable cells was calculated for each concentration as ((*OD_T_/OD_C_*) × 100), where *OD_T_* and *OD_C_* corresponded to the absorbance of the treated and control cells, respectively. The IC_50_ was calculated by using linear regression analysis.

### 3.5. Antitumor Assay

The influence of synthesized compounds on the methylation of DNA of tumor sarcoma 180 (S-180) was studied in vitro. Transplanted S-180 was isolated from animals after slaughter by decapitation under ether narcosis. A 3 × 10^−6^ M solution of compounds (solvent: carboxymethylcellulose) was added to the tumor homogenate (12.5 mL of a solution per 10 g of tumor), and the mixture was incubated for 24 h at 37 °C. After the incubation, tumor DNA was extracted using the phenol–chloroform method [33]. Hydrolysis to the nitrogen bases was carried out in sealed glass ampoules in 85% formic acid at 176 °C for 1 h (0.1 mL of acid per 1 mg of DNA). The separation of nitrogen bases (guanine (G), cytosine (C), 5-methylcitosine (5-MC), adenine (A), and thymine (T)) was produced by thin-layer chromatography in the solvent *n*-butanol/water/ammonia. Spectrophotometry of the eluates of all bases was made against eluates from the respective control areas of the chromatograms. In our studies, the well-known antitumor drug doxorubicin (manufactured by EBEWE Pharma Ges.m.b.H. Nfg. KG, 4866 Unterach, Austria) was used as a standard preparation. The data were statistically processed using Student’s and Fischer’s tests (*p* < 0.05).

## 4. Conclusions

Through a multistage organic synthesis, new tetraheterocyclic compounds were obtained, and their antitumor activity was investigated. The results of the biological investigations revealed that all new synthesized amino derivatives of the pyrido[3′,2′:4,5]furo(thieno)[3,2-*d*]pyrimidines (with the exception of compounds **7a**,**i**,**j**) showed DNA demethylation activity, and three of them (**5b**,**f** and **7b**) showed pronounced antitumor activity. Moreover, several amino derivatives of two new five-membered heterocyclic systems, imidazo[1,2-*c*]pyrimidine and pyrimido[1,2-*c*]pyrimidine, were synthesized.

## Figures and Tables

**Table 1:**
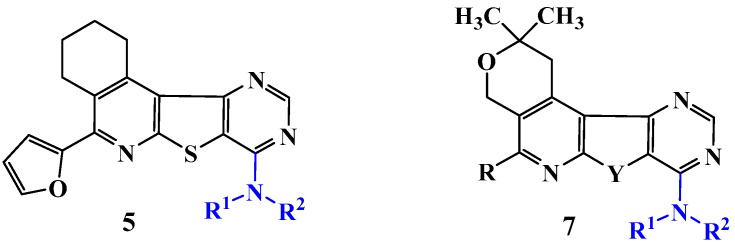
Content and inhibition of 5-MC of DNA samples of tumors under the influence of the studied compounds **5** and **7** in vitro.

DNA, Sarcoma-180, and Compound	N (R^1^R^2^)	R	Y	Content of Bases in DNA, mol %		Inhibition of the Level of DNA Methylation, %
Source of DNA	−	−	−	5-MC ± ζ	G + C + 5-MC	
S-180	−	−	−	0.64 ± 0.02	42.66	
**5a**	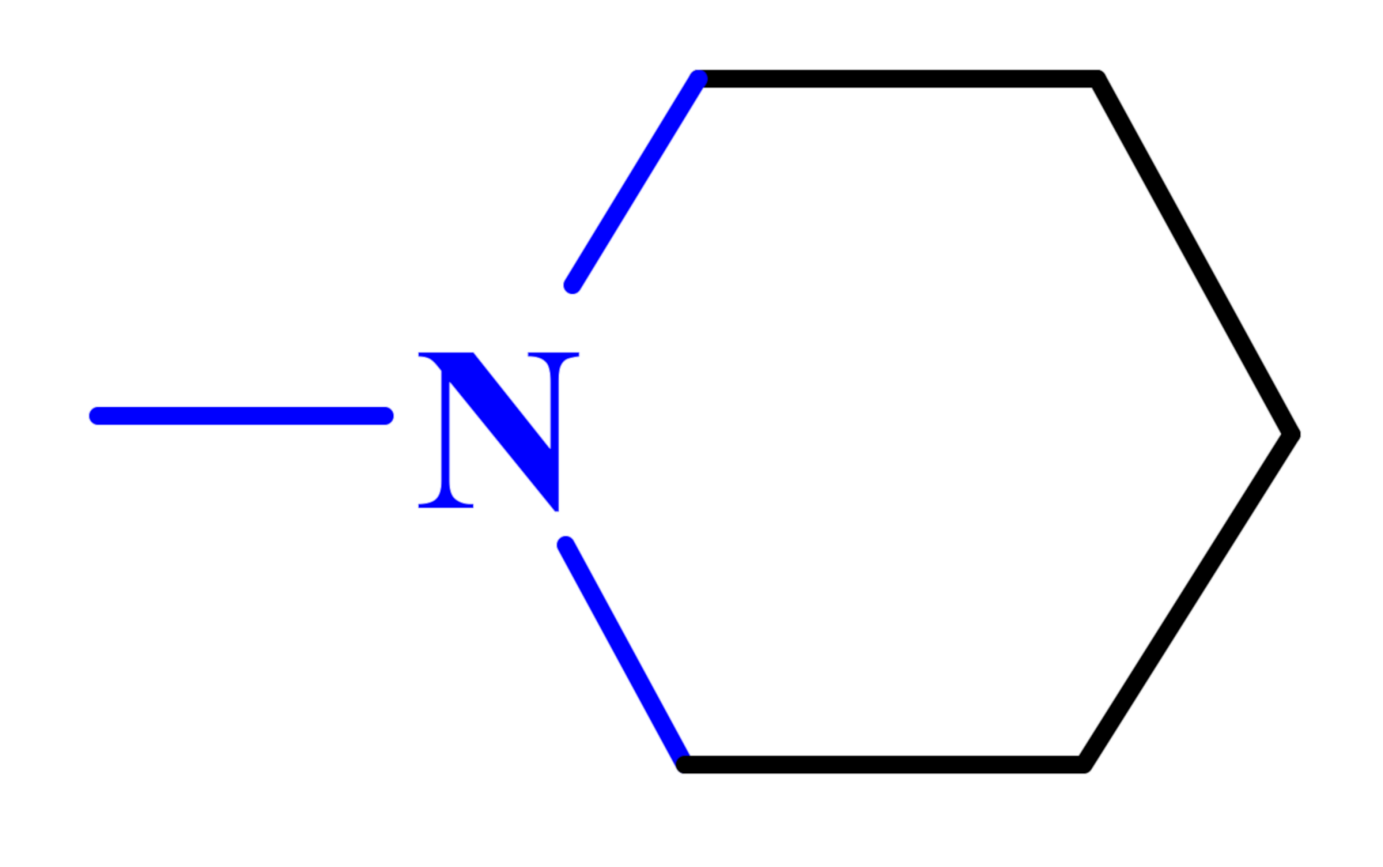	−	−	0.38 ± 0.02	42.50	40.6
**5b**	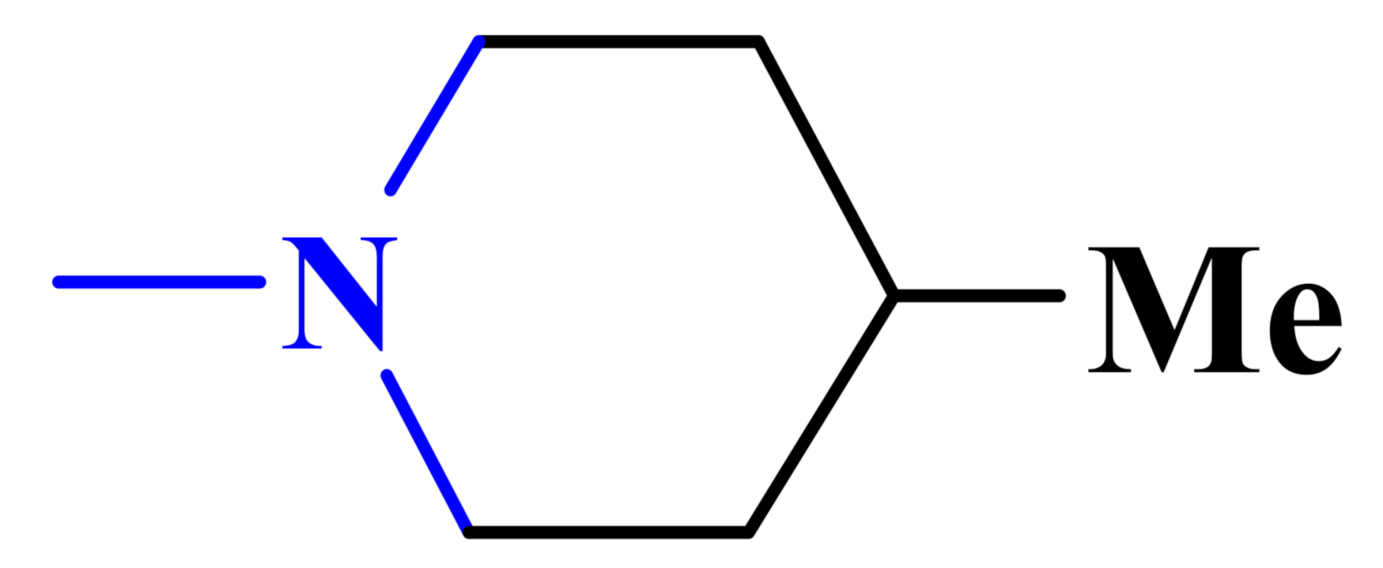	−	−	0.25 ± 0.02	43.74	60.9
**5c**	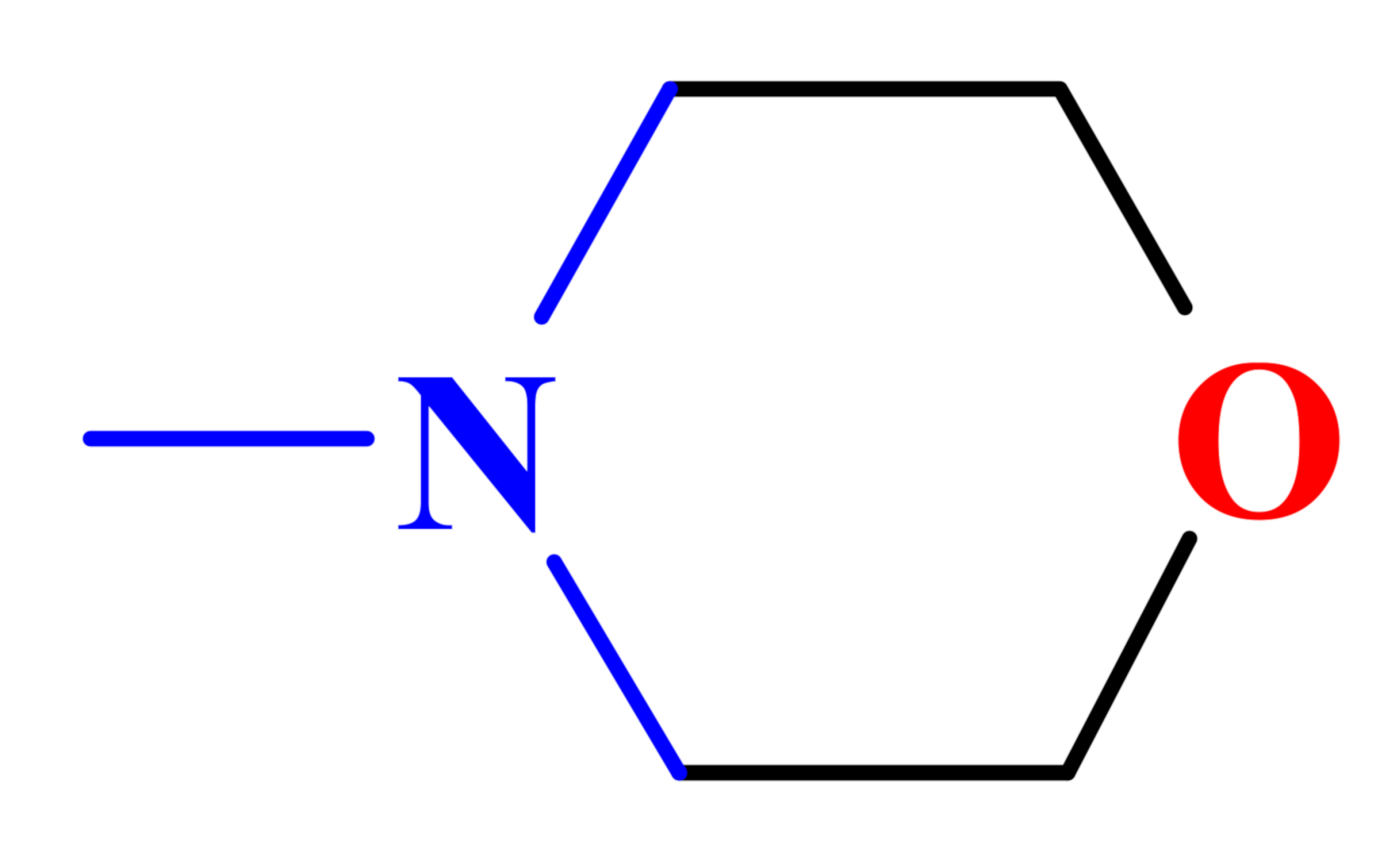	−	−	0.49 ± 0.02	42.44	23.0
**5d**	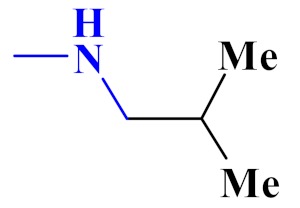	−	−	0.42 ± 0.03	42.58	34.4
**5e**	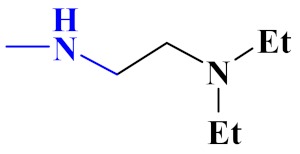	−	−	0.46 ± 0.02	42.44	28.1
**5f**	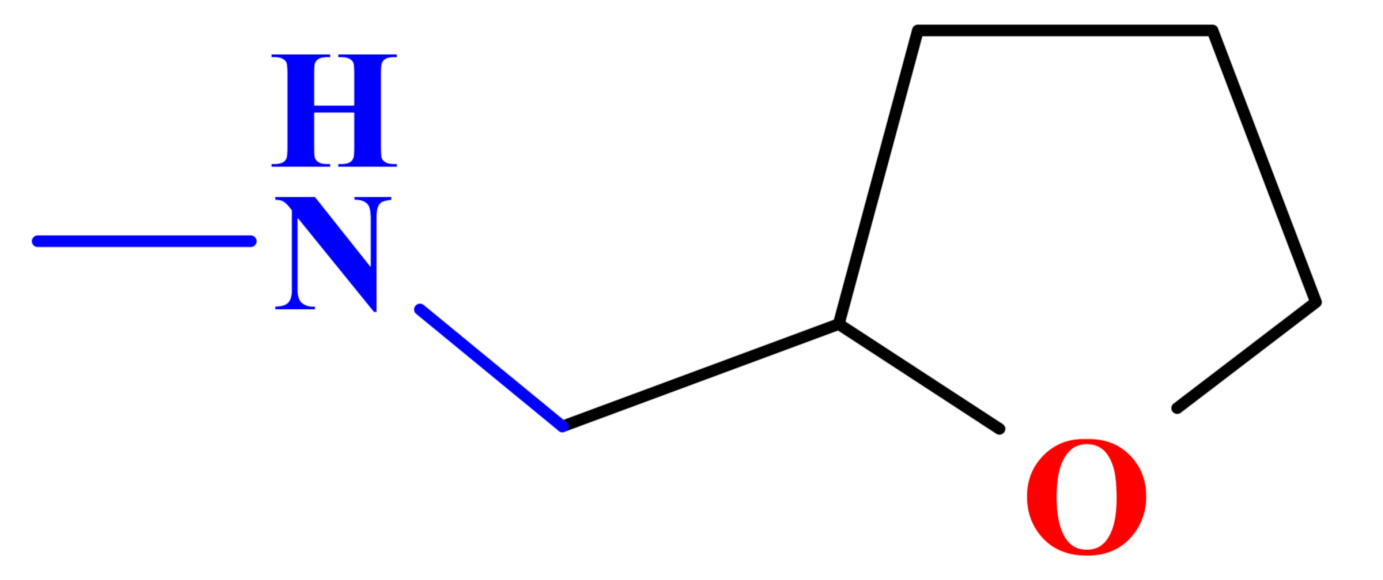	−	−	0.07 ± 0.02	44.02	89.1
**7a**	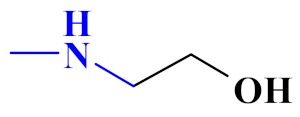	*i*-Pr	O	0.85 ± 0.03	43.50	−
**7b**	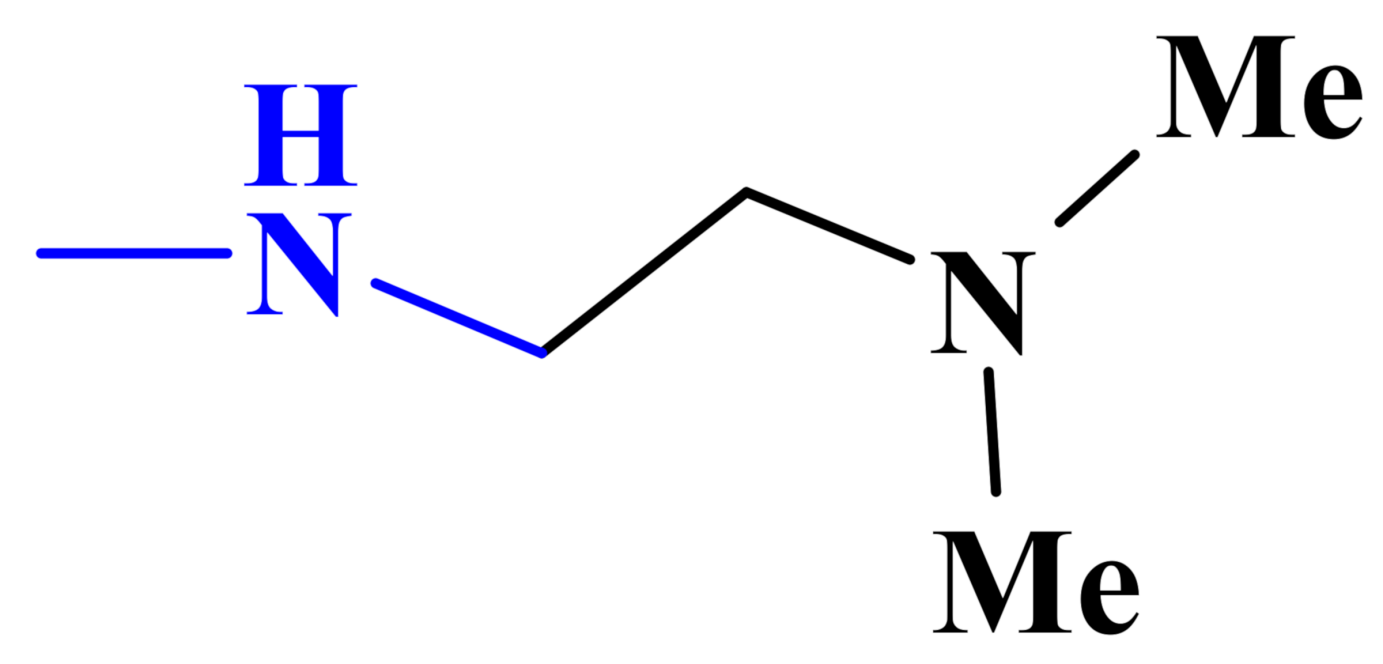	*i*-Pr	O	0.24 ± 0.01	44.16	62.5
**7c**	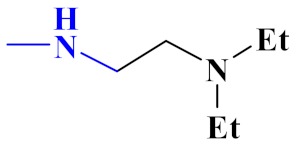	*i*-Pr	O	0.37 ± 0.01	42.72	42.2
**7d**	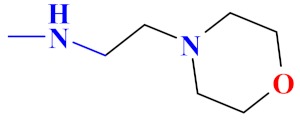	*i*-Pr	O	0.33 ± 0.01	43.44	48.4
**7e**	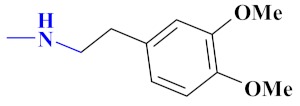	*i*-Pr	O	0.28 ± 0.01	44.00	56.2
**7f**	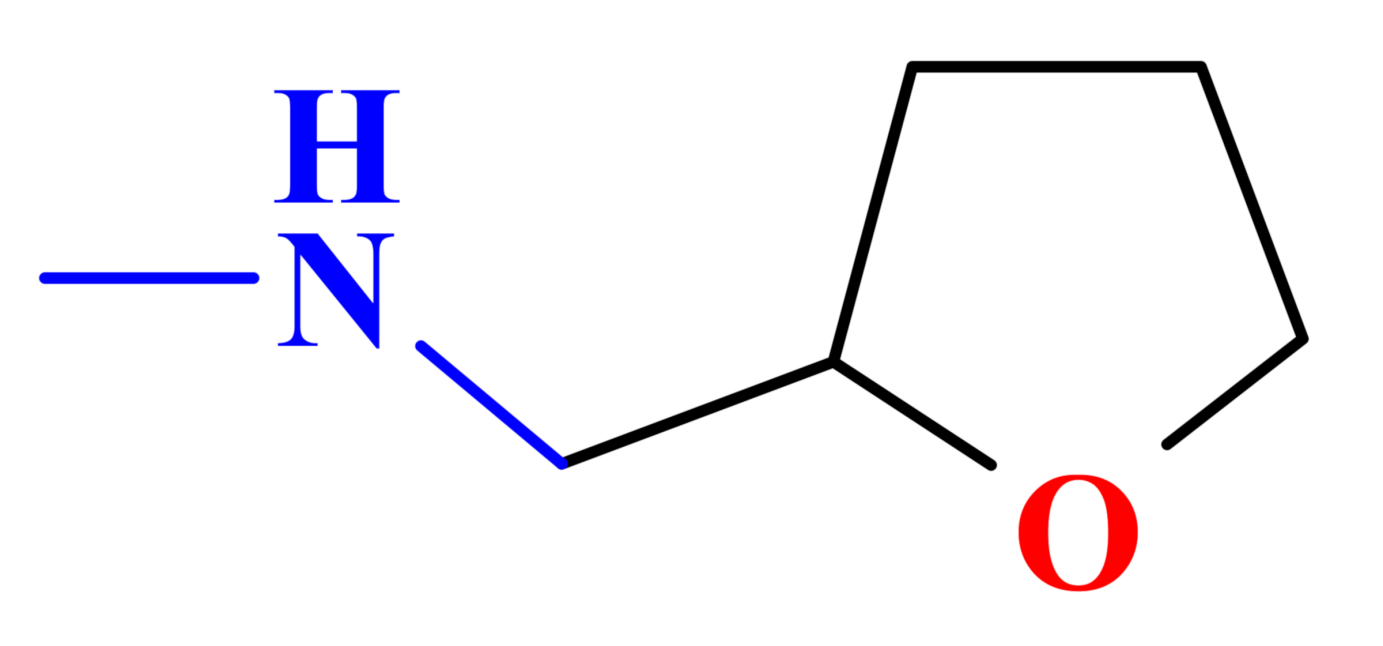	*i*-Pr	O	0.50 ± 0.02	42.82	21.9
**7g**	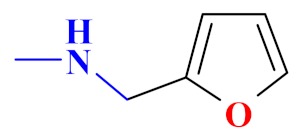	*i*-Pr	O	0.38 ± 0.02	43.32	40.6
**7h**	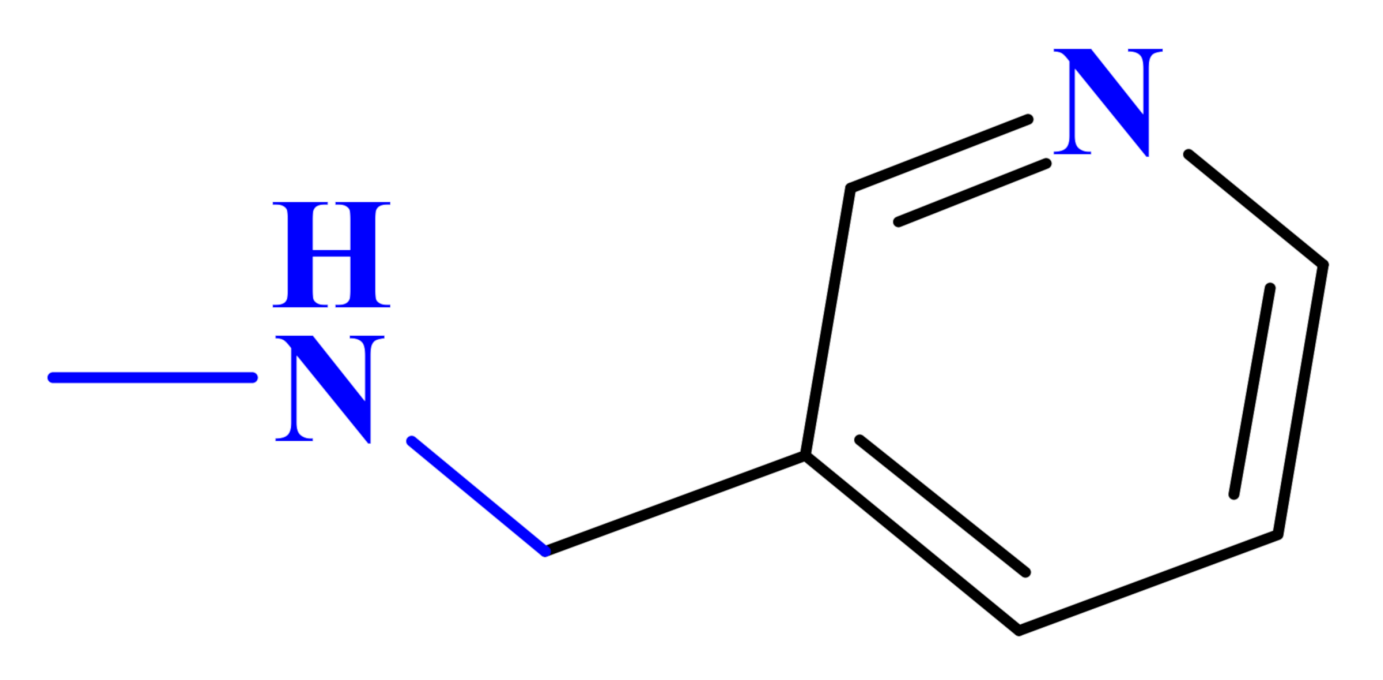	*i*-Pr	O	0.42 ± 0.02	43.20	34.4
**7i**	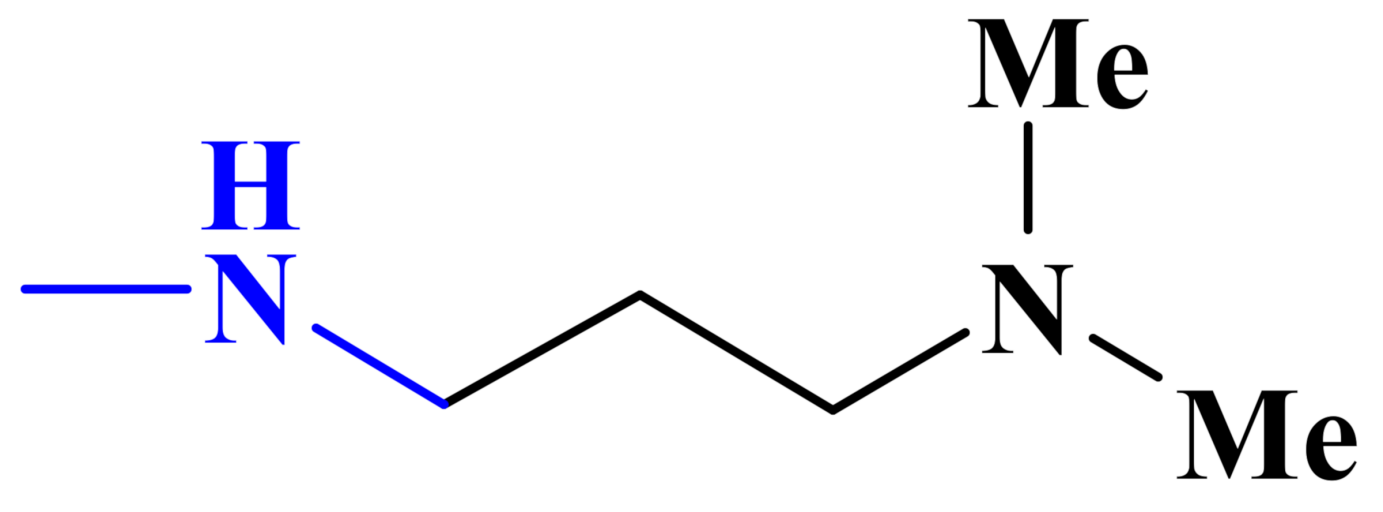	*i*-Pr	O	0.52 ± 0.02	44.64	−
**7j**	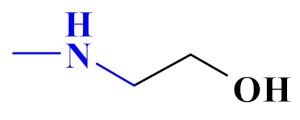	*i*-Bu	O	0.54 ± 0.01	43.20	−
**7k**	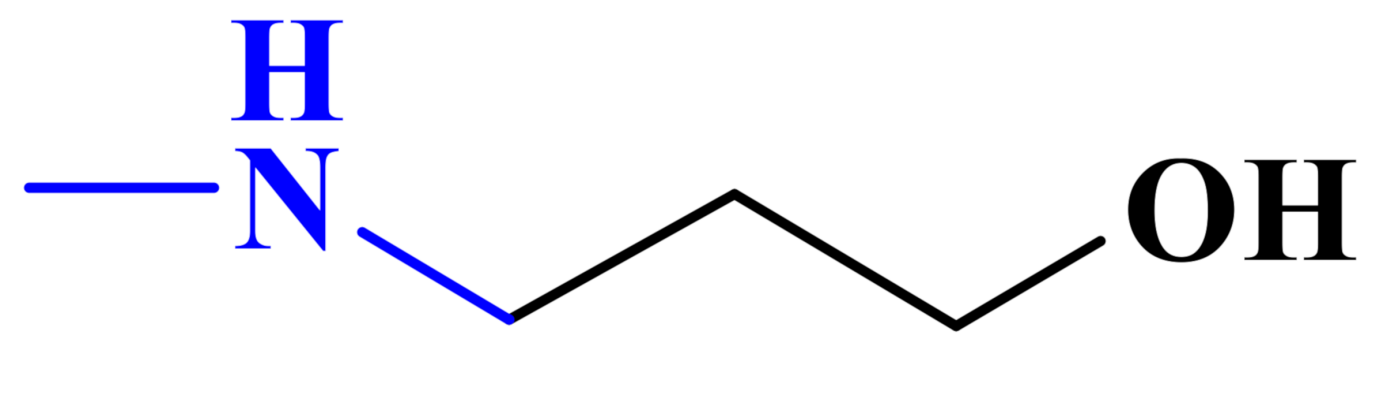	*i*-Bu	O	0.52 ± 0.01	42.70	18.8
**7l**	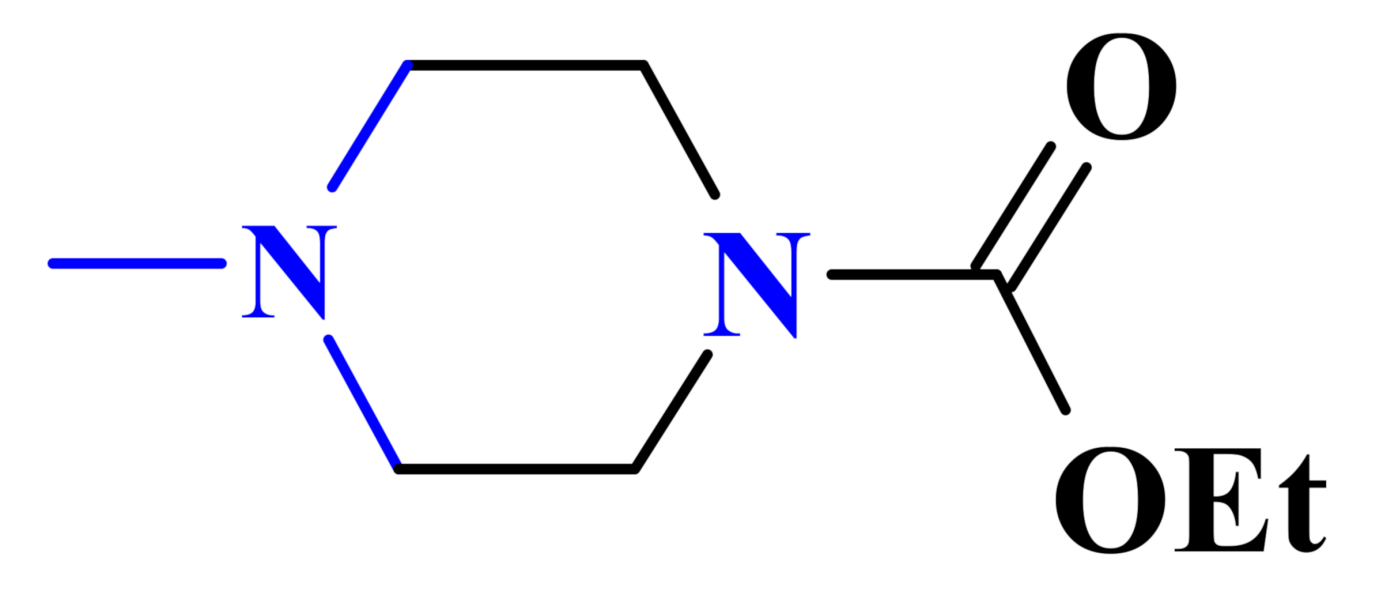	*i*-Pr	S	0.45 ± 0.03	43.76	29.7
**7m**	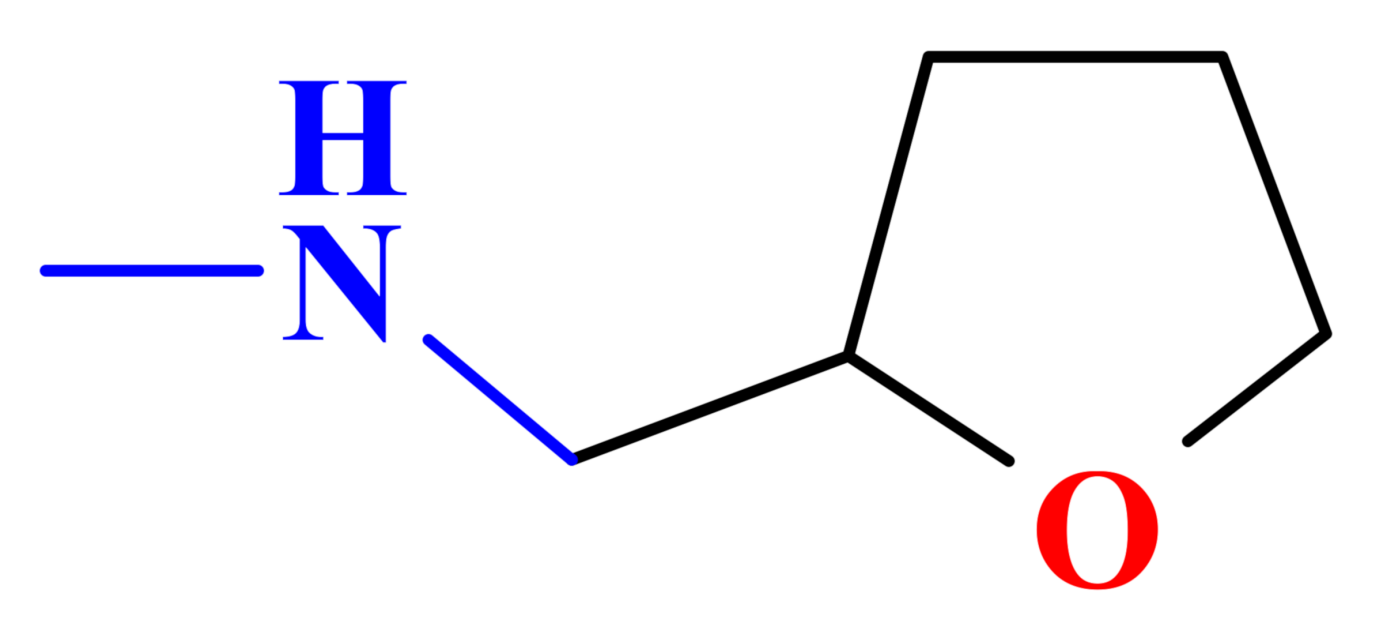	*i*-Pr	S	0.40 ± 0.02	43.06	37.5
Doxorubicin				0.21 ± 0.01	43.41	67.2

Number of definitions: seven. These changes were reliable (*p* < 0.05) compared to the control.

**Table 2:** Binding energies, Internal Coordinate Mechanics (ICM) binding scores, and Protein Data Bank (PDB) structure IDs for which docking showed the highest results.

Compound	Binding Energy (kcal/mol)	ICM Score	PDB ID
**5b**	−14.73	−34.61	4WXX
**5f**	−15.59	−36.41	4DA4
**7b**	−10.64	−28.01	4YOC

## Supplementary Materials

The copies of ^1^H-NMR and ^13^C-NMR spectra for all new synthesized compounds were submitted along with the manuscript.

## References

[1] Vanyushin B.F. (2016). Enzymatic DNA methylation: What it is needed for in the cell. JSM Genet. Genom..

[2] Vanyushin B.F. (2005). Enzymatic DNA methylation is an epigenetic control for genetic functions of the cell. Biochemistry.

[3] Worm J., Guldberg P. (2002). DNA methylation: An epigenetic pathway to cancer and a promising target for anticancer therapy. J. Oral Pathol. Med..

[4] Bartosik M., Ondrouskova E. (2016). Novel approaches in DNA methylation studies–MS-HRM analysis and electrochemistry. Klin. Onkol..

[5] Mikeska T., Craig J.M. (2014). DNA methylation biomarkers: Cancer and beyond. Genes.

[6] Beltran-Garcia J., Osca-Verdegal R., Mena-Molla S., Garcia-Gimenez J.L. (2019). Epigenetic IVD tests for personalized precision medicine in cancer. Front. Genet..

[7] Howell P.M., Liu Z., Khong H.T. (2010). Demethylating agents in the treatment of cancer. Pharmaceuticals.

[8] Hayakawa M., Kaizawa H., Moritomo H., Koizumi T., Ohishi T., Yamano M., Okada M., Ohta M., Tsukamoto S., Raynaud F.I. (2007). Synthesis and biological evaluation of pyrido[3′,2′:4,5]furo[3,2-*d*]pyrimidine derivatives as novel PI3 kinase p110a inhibitors. Bioorganic Med. Chem. Lett..

[9] Taltavull J., Serrat J., Gracia J., Gavalda A., Andres M., Cordoba M., Miralpeix M., Vilella D., Beleta J., Ryder H. (2010). Synthesis and biological activity of pyrido[3′,2′:4,5]thieno[3,2-*d*]pyrimidines as phosphodiesterase type 4 Inhibitors. J. Med. Chem..

[10] Agarwal A., Louise-May S., Thanassi J.A., Podos S.D., Cheng J., Thoma C., Liu C., Wiles J.A., Nelson D.M., Phadke A.S. (2007). Small molecule inhibitors of *E. coli* primase, a novel bacterial target. Bioorg. Med. Chem. Lett..

[11] Kjellerup L., Gordon S., Cohrt K.O., Brown W.D., Fuglsang A.T., Winther A.-M.L. (2017). Identification of antifungal H^+^-ATPase inhibitors with effect on the plasma membrane potential. Antimicrob. Agents Chemother..

[12] Sirakanyan S.N., Ovakimyan A.A., Noravyan A.S., Dzhagatspanyan I.A., Shakhatuni A.A., Nazaryan I.M., Hakopyan A.G. (2013). Synthesis and antyconvulsive activity of 8-amino substitudet cyclopenta[4′,5′]pyrido[3′,2′:4,5]thieno[3,2-*d*]pyrimidines. Pharm. Chem. J..

[13] Sirakanyan S.N., Ovakimyan A.A., Noravyan A.S., Minasyan N.S., Dzhagatspanyan I.A., Nazaryan I.M., Hakopyan A.G. (2014). Synthesis and neurotropic activity of 8-amino derivatives of condensed thieno[3,2-*d*]- and furo[3,2-*d*]pyrimidines. Pharm. Chem. J..

[14] Sirakanyan S.N., Akopyan E.K., Paronikyan R.G., Akopyan A.G., Ovakimyan A.A. (2016). Synthesis and anticonvulsant activity of 7(8)-amino derivatives of condensed thieno[3,2-*d*]pyrimidines. Pharm. Chem. J..

[15] Sirakanyan S.N., Geronikaki A., Spinelli D., Hakobyan E.K., Kartsev V.G., Petrou A., Hovakimyan A.A. (2018). Synthesis and antimicrobial activity of new amino derivatives of pyrano[4″,3″:4′,5′]pyrido[3′,2′:4,5]thieno[3,2-d]pyrimidine. An. Acad. Bras. Cienc..

[16] Sirakanyan S.N., Spinelli D., Geronikaki A., Kartsev V.G., Hakobyan E.K., Hovakimyan A.A. (2019). Synthesis and antimicrobial activity of new derivatives of pyrano[4″,3″:4′,5′]pyrido[3′,2′:4,5]thieno[3,2-*d*]pyrimidine and new heterocyclic systems. Synth. Commun..

[17] Sirakanyan S.N., Paronikyan E.G., Noravyan A.S. (2001). The Chemistry and Biological Activity of Nitrogen-containing Heterocycles and Alkaloids.

[18] Sirakanyan S.N., Paronikyan E.G., Noravyan A.S. (2003). The Chemistry and Biological Activity of Synthetic and Natural Compounds.

[19] Paronikyan E.G., Sirakanyan S.N., Noravyan A.S., Paronikyan R.G., Dzhagatspanyan I.A. (2001). Synthesis and anticonvulsant activity of pyrazolo[3,4-*b*]pyrano(thiopyrano)[4,3-*d*]pyridine and pyrazolo[3,4-*c*]isoquinoline derivatives. Pharm. Chem. J..

[20] Sirakanyan S.N., Paronikyan E.G., Ghukasyan M.S., Noravyan A.S. (2010). Synthesis of 8-amino derivatives of condensed furo[3,2-d]pyrimidines. Chem. Heterocycl. Compd..

[21] Brown R., Plumb J.A. (2004). Demethylation of DNA by decitabine in cancer chemotherapy. Expert Rev. Anticancer Ther..

[22] Fahy J., Jeltsch A., Arimondo P.B. (2012). DNA methyltransferase inhibitors in cancer: A chemical therapeutic patent overview and selected clinical studies. Expert Opin. Ther. Pat..

[23] Christman J.K., Sheikhnejad G., Marasco C.J., Sufrin J.R. (1995). 5-Methyl-2′-deoxycytidine in single-stranded DNA can act in cis to signal de novo DNA methylation. Proc. Natl. Acad. Sci. USA.

[24] Gowher H., Jeltsch A. (2018). Mammalian DNA methyltransferases: New discoveries and open questions. Biochem. Soc. Trans..

[25] Xie T., Yu J., Fu W., Wang Z., Xu L., Chang S., Wang E., Zhu F., Zeng S., Kang Y. (2019). Insight into the selective binding mechanism of DNMT1 and DNMT3A inhibitors: A molecular simulation study. Phys. Chem. Chem. Phys..

[26] Ren W., Gao L., Song J. (2018). Structural basis of DNMT1 and DNMT3A-mediated DNA methylation. Genes.

[27] Callebaut I., Courvalin J.C., Mornon J.P. (1999). The BAH (bromo-adjacent homology) domain: A link between DNA methylation, replication and transcriptional regulation. FEBS Lett..

[28] Abagyan R., Totrov M., Kuznetsov D. (1994). ICM–A new method for protein modeling and design: Applications to docking and structure prediction from the distorted native conformation. J. Comput. Chem..

[29] Bottegoni G., Kufareva I., Totrov M., Abagyan R. (2009). Four-dimensional docking: A fast and accurate account of discrete receptor flexibility in ligand docking. J. Med. Chem..

[30] Nicola G., Smith C.A., Abagyan R. (2008). New method for the assessment of all drug-like pockets across a structural genome. J. Comput. Biol..

[31] Schapira M., Totrov M., Abagyan R. (1999). Prediction of the binding energy for small molecules, peptides and proteins. J. Mol. Recognit..

[32] Arabyan E., Hakobyan A., Kotsinyan A., Karalyan Z., Arakelov V., Arakelov G., Nazaryan K., Simonyan A., Aroutiounian R., Ferreira F. (2018). Genistein inhibits African swine fever virus replication in vitro by disrupting viral DNA synthesis. Antivir. Res..

[33] Vanyushin B.F., Masin F.N., Vasiliev V.R., Belozersky A.N. (1973). The content of 5-methylcytosine in animal DNA: The species and tissue specifity. Biochim. Biophys. Acta.

