# Supplementary material for: Synthesis, Antitumor Activity, and Docking Analysis of New Pyrido[3′,2′:4,5]furo(thieno)[3,2-d]pyrimidin-8-amines
Source: Molecules. 2019 Oct 31;24(21):3952. doi: 10.3390/molecules24213952 (PMC6864781; doi:10.3390/molecules24213952)

Supporting Information File

for

Synthesis, Antitumor Activity and Docking Analysis of New Pyrido[3',2':4,5]furo(thieno)[3,2-*d*]pyrimidin-8-amines

Samvel N. Sirakanyan[1],* Domenico Spinelli[2],* Athina Geronikaki[3], Elmira K. Hakobyan[1], Harutyun Sahakyan[4], Erik Arabyan[5], Hovakim Zakaryan[5], Lusine E. Nersesyan[1], Anahit S. Aharonyan[1], Irina S. Danielyan[1], Rafayel E. Muradyan[1], and Anush A. Hovakimyan[1]

[1] *Scientific Technological Center of Organic and Pharmaceutical Chemistry of National Academy of Science of Republic of Armenia, Institute of Fine Organic Chemistry of A.L. Mnjoyan, Armenia 0014, Yerevan, Ave. Azatutyan 26; e-mail: shnnr@mail.ru*

[2] *Dipartimento di Chimica G. Ciamician, Alma Mater Studiorum-Università di Bologna, Via F. Selmi 2, Bologna 40126, Italy; e-mail: domenico.spinelli@unibo.it*

[3] *Aristotle University of Thessaloniki, School of Pharmacy, Thessaloniki 54124, Greece; e-mail: geronik@pharm.auth.gr*

[4] *Department of Bioengineering, Bioinformatics and Molecular Biology, Russian-Armenian University, Armenia 0051, Yerevan; 123 Hovsep Emin str.; e-mail: sahakyanhk@gmail.com*

[5] *Institute of Molecular Biology of NAS, Hasratyan 7, 0014, Yerevan, Armenia; e-mail: h_zakaryan@mb.sci.am*

*Correspondence: domenico.spinelli@unibo.it (D.S.); shnnr@mail.ru (S.N.S); Tel.: +39-051-209-9478 (D.S.); +374-91-32-15-99 (S.N.S.)

Copies of ^{1}H and ^{13}C NMR spectra for all new synthesized compounds

Molecular Structure Research Centre, Yerevan, Armenia, Varian Mercury-300VX   H1 300.088 MHz, nt=16, np=32000, temp=30.0 C, lb=-0.2, solvent=DMSO/CCl4 1/3   Aug 2 2019

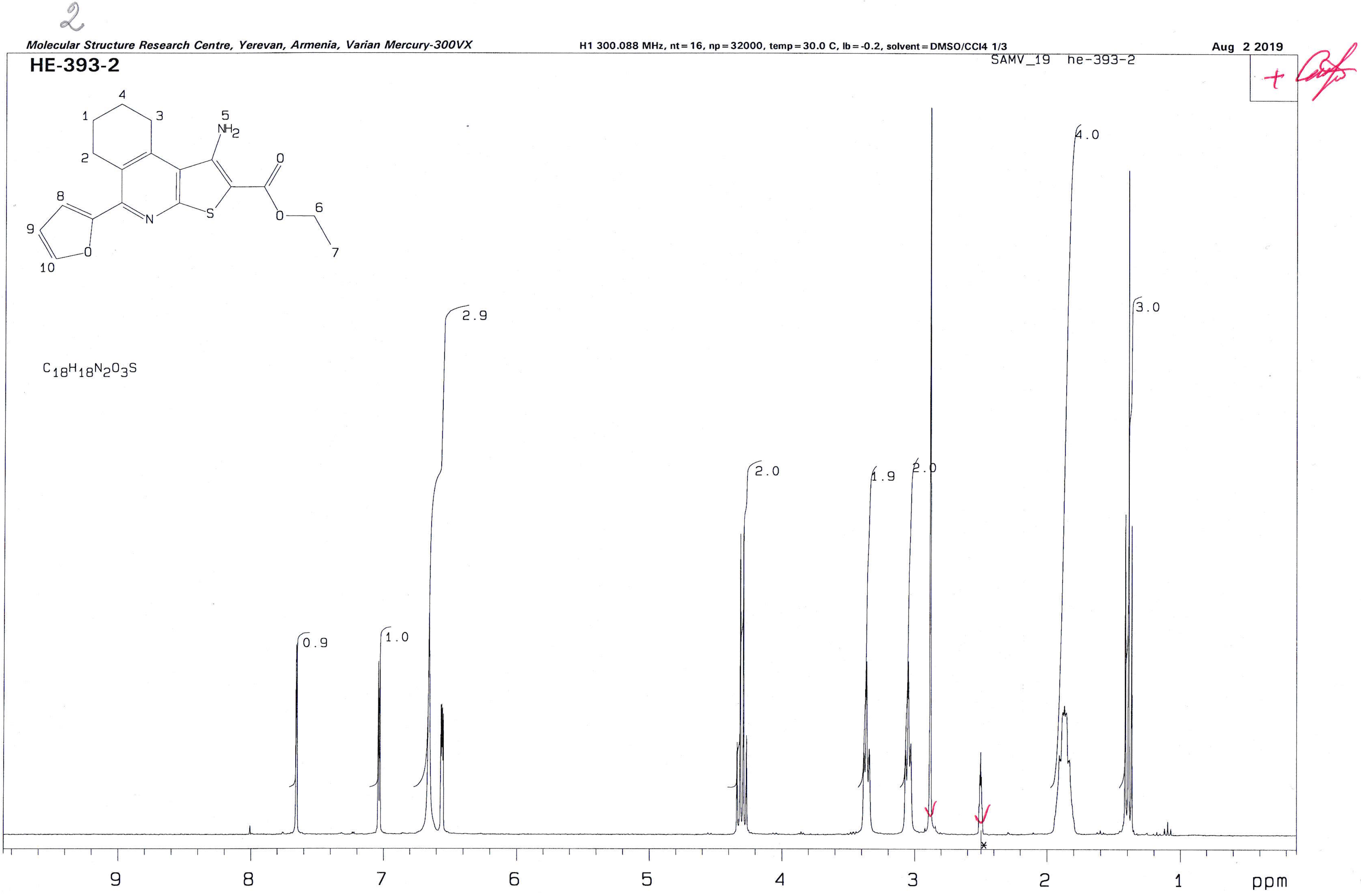

Molecular Structure Research Centre, Yerevan, Armenia, Varian Mercury-300VX

C13 75.465 MHz, nt=512, np=19998, temp=30.0 C, lb=1.0, solvent=DMSO/CCl4 1/3

Aug 2 2019

SAMV_19 he-393-2

HE-393-2

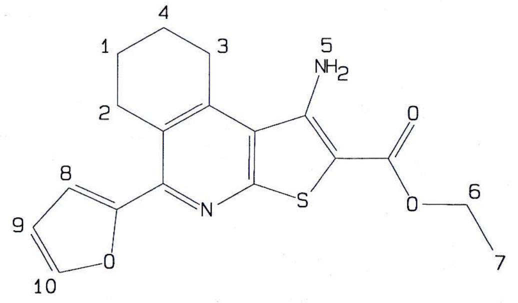

$C_{18}H_{18}N_2O_3S$

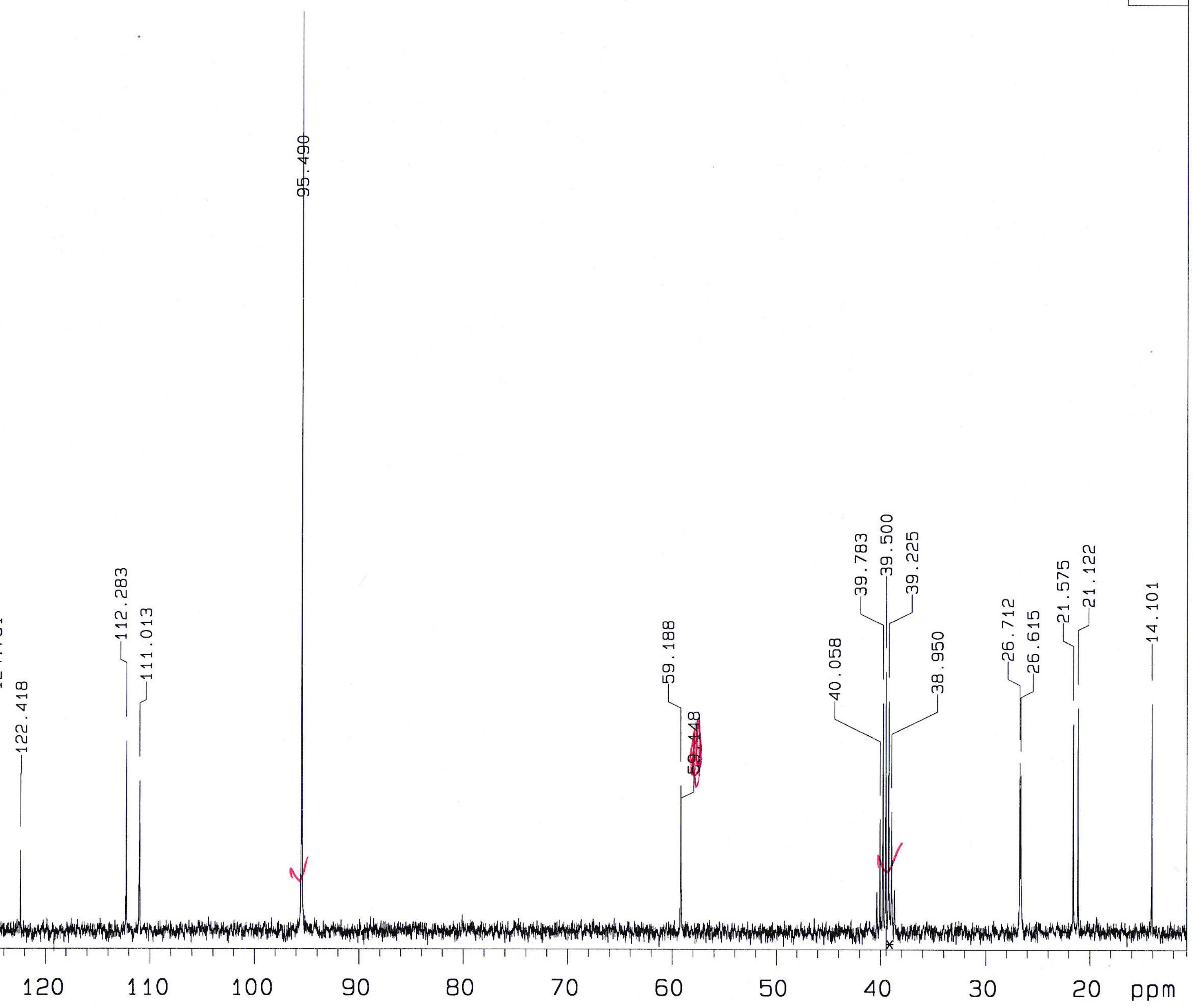

3

Molecular Structure Research Centre, Yerevan, Armenia, Varian Mercury-300VX **H1 300.077 MHz, nt = 16, np = 16000, temp = 30.0 C, lb = -0.2, solvent = DMSO/CCl4 1/3** **Oct 30 2006**

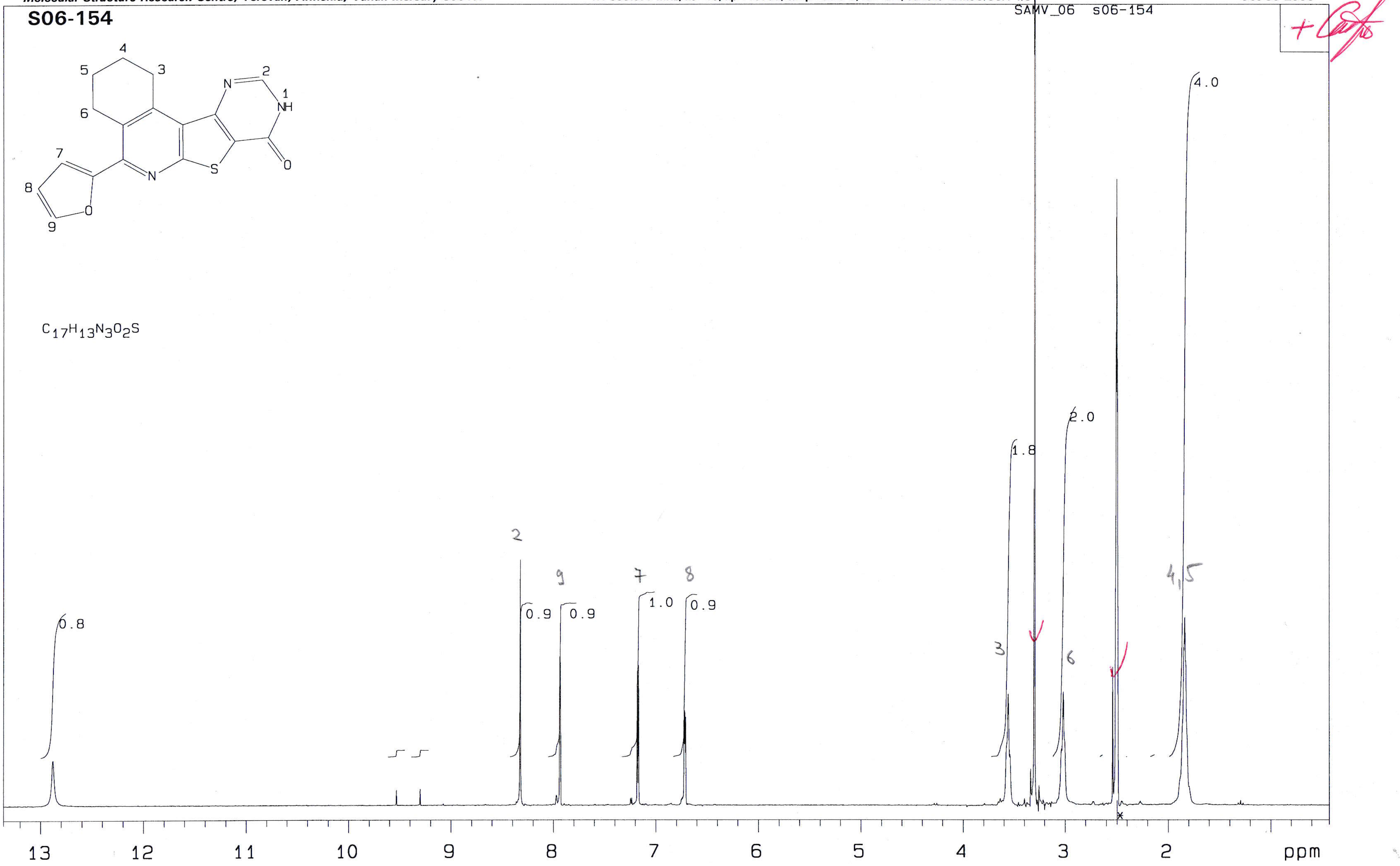

4

Molecular Structure Research Centre, Yerevan, Armenia, Varian Mercury-300VX

H1 300.088 MHz, nt = 16, np = 16000, temp = 30.0 C, lb = -0.2, solvent = DMSO/CCl4 1/3

Jul 17 2019

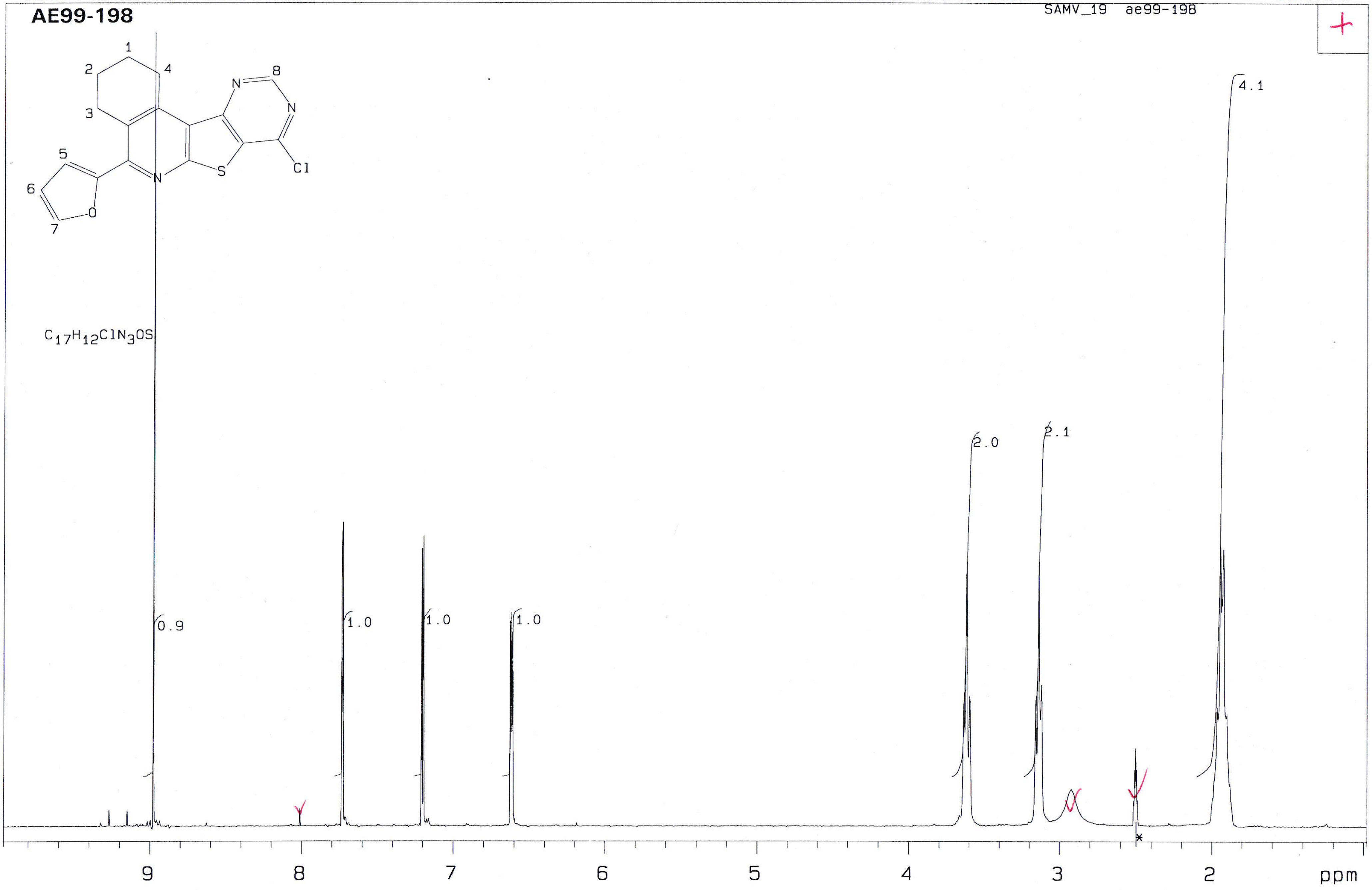

4

Molecular Structure Research Centre, Yerevan, Armenia, Varian Mercury-300VX **C13 75.465 MHz, nt=256, np=19998, temp=30.0 C, lb=1.0, solvent=DMSO/CCl4 1/3** **Jul 17 2019**

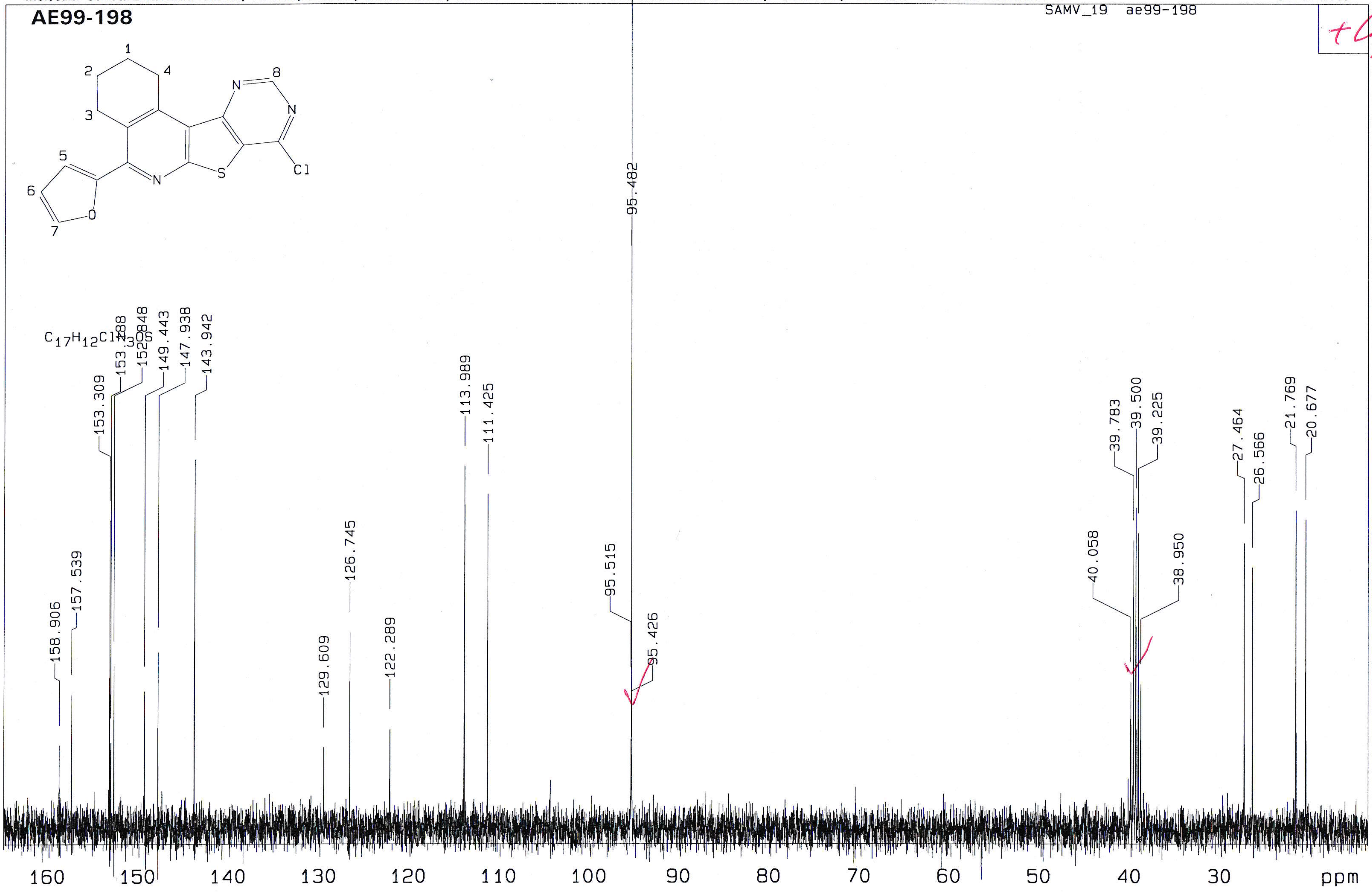

5a

Molecular Structure Research Centre, Yerevan, Armenia, Varian Mercury-300VX **H1 300.088 MHz, nt = 16, np = 16000, temp = 30.0 C, lb = -0.2, solvent = DMSO/CCl4 1/3** **Jul 18 2019**

AE99-200

SAMV_19 ae99-200

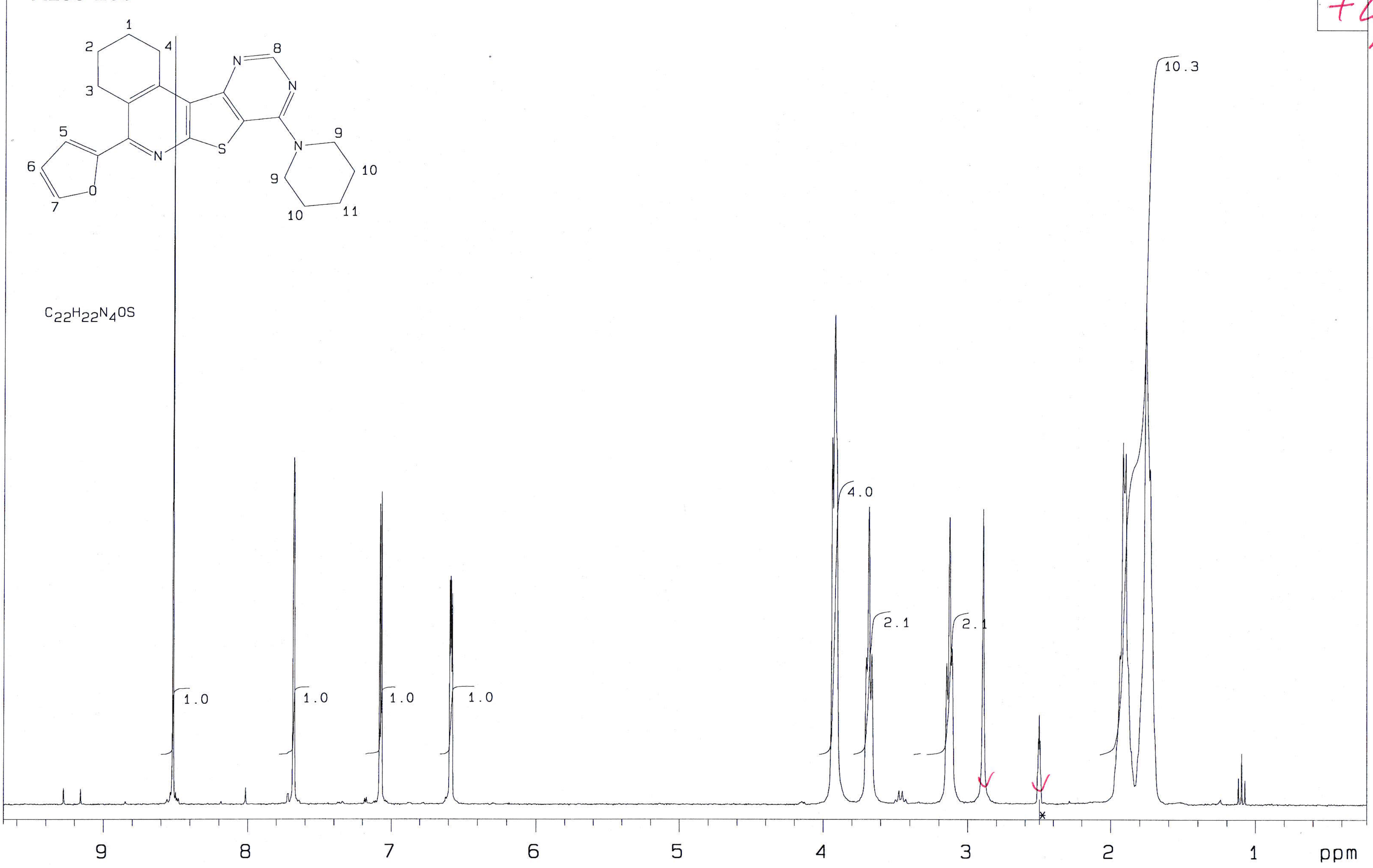

5a

Molecular Structure Research Centre, Yerevan, Armenia, Varian Mercury-300VX C13 75.465 MHz, nt=496, np=19998, temp=30.0 C, lb=1.0, solvent=DMSO/CCl4 1/3 **Jul 18 2019**

SAMV_19 ae99-200

AE99-200

$C_{22}H_{22}N_4OS$

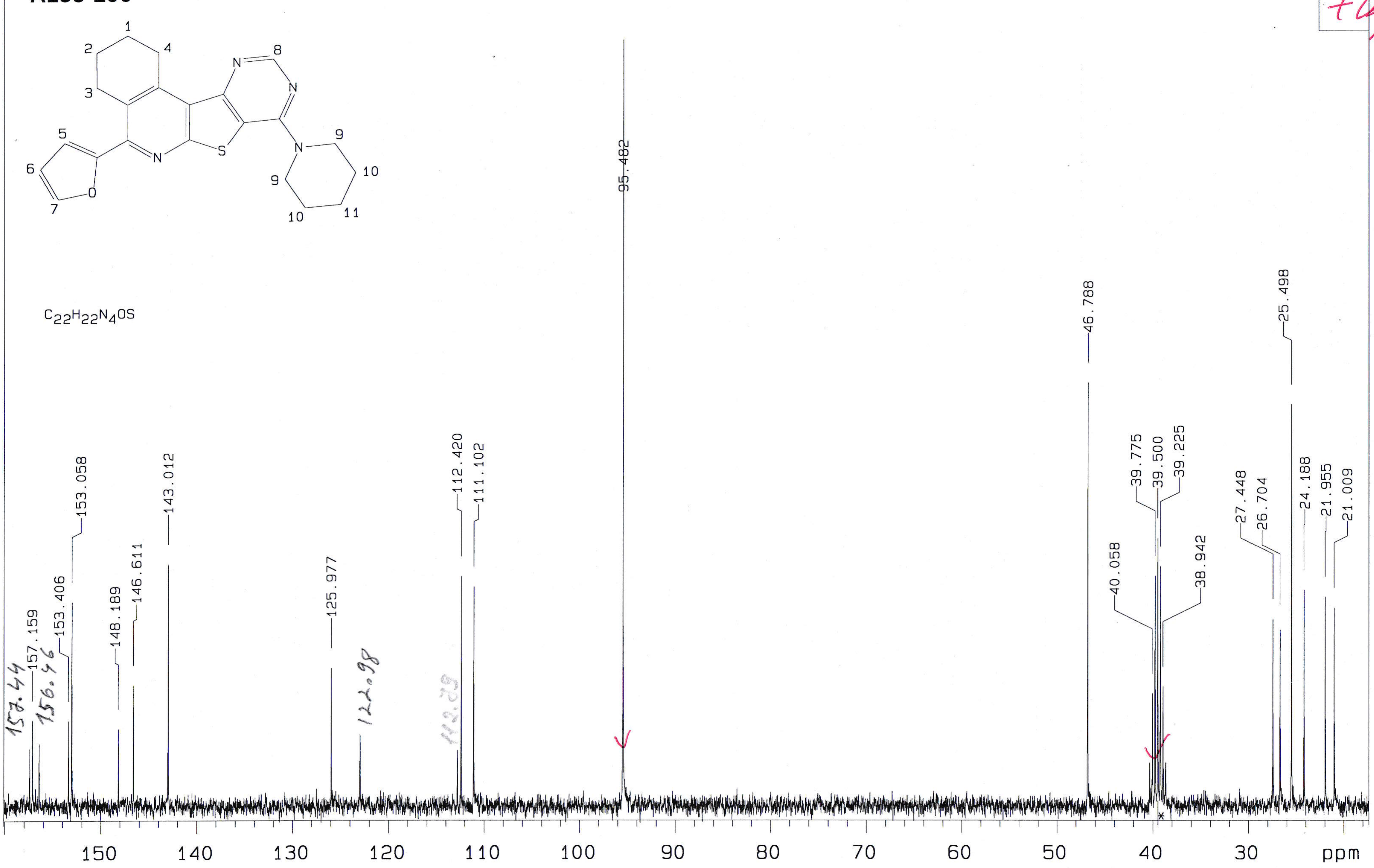

5b

Molecular Structure Research Centre, Yerevan, Armenia, Varian Mercury-300VX

H1 300.088 MHz, nt = 16, np = 32000, temp = 30.0 C, lb = -0.2, solvent = DMSO/CCl4 1/3

Jul 4 2019

NOCI_19 ae99-215

AE99-215

$C_{23}H_{24}N_4OS$

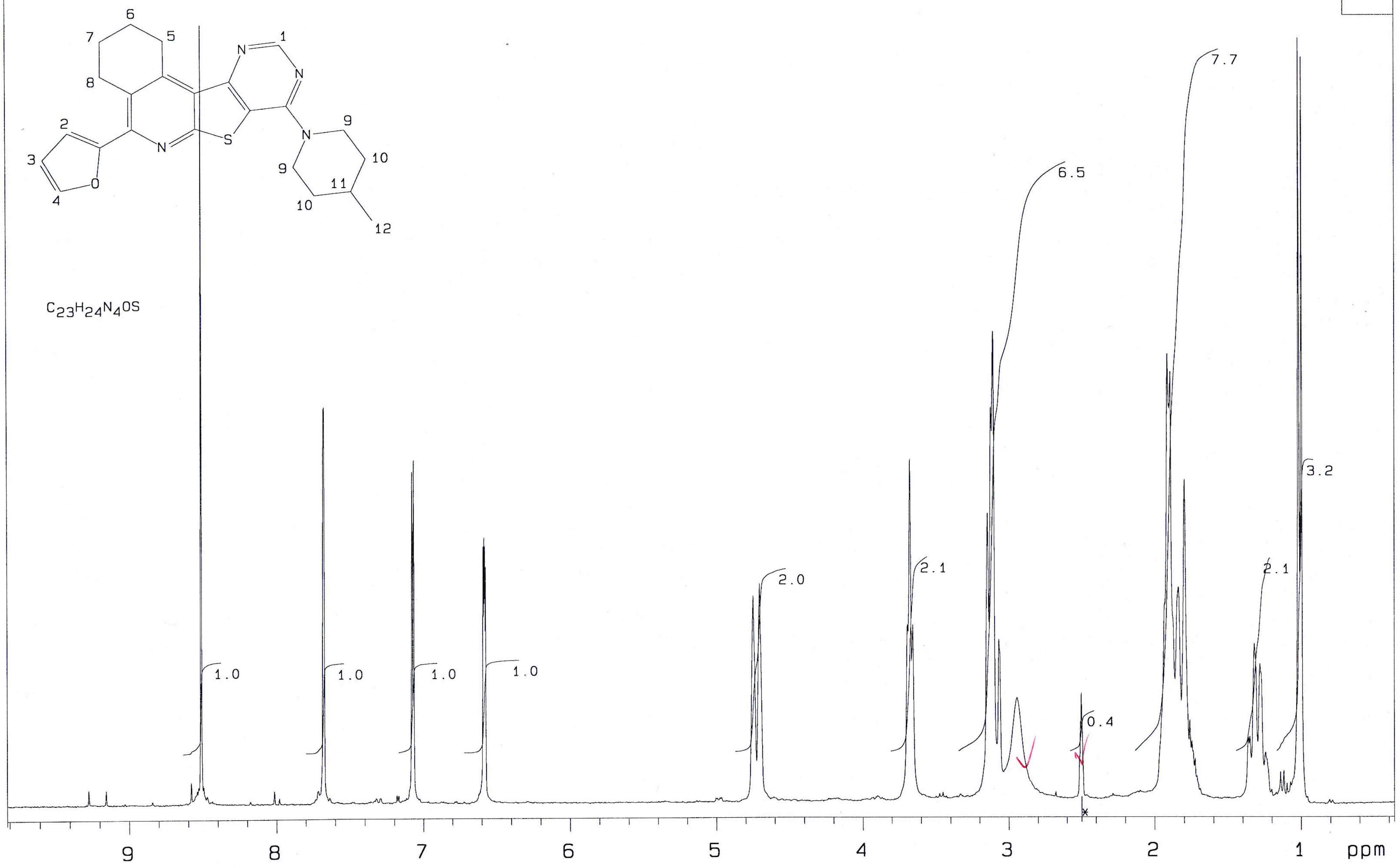

5b

Molecular Structure Research Centre, Yerevan, Armenia, Varian Mercury-300VX

C13 75.465 MHz, nt=720, np=19998, temp=30.0 C, lb=1.0, solvent=DMSO/CCl4 1/3

Jul 4 2019

NOCI_19 ae99-215

AE99-215

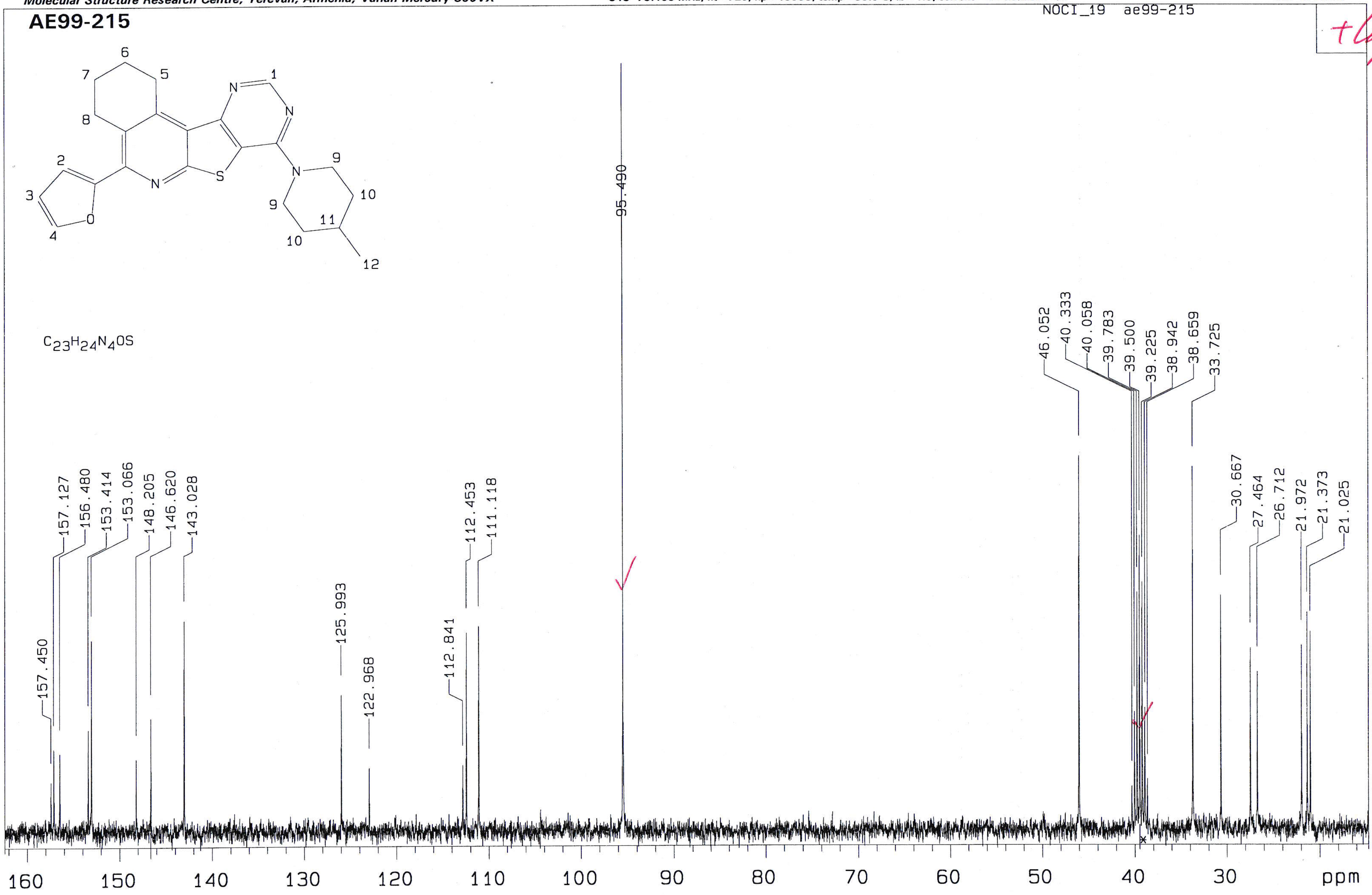

5c

Molecular Structure Research Centre, Yerevan, Armenia, Varian Mercury-300VX | H1 300.088 MHz, nt = 16, np = 16000, temp = 30.0 C, lb = -0.2, solvent = DMSO/CCl4 1/3 | Jul 18 2019

SAMV_19 ae99-199

AE99-199

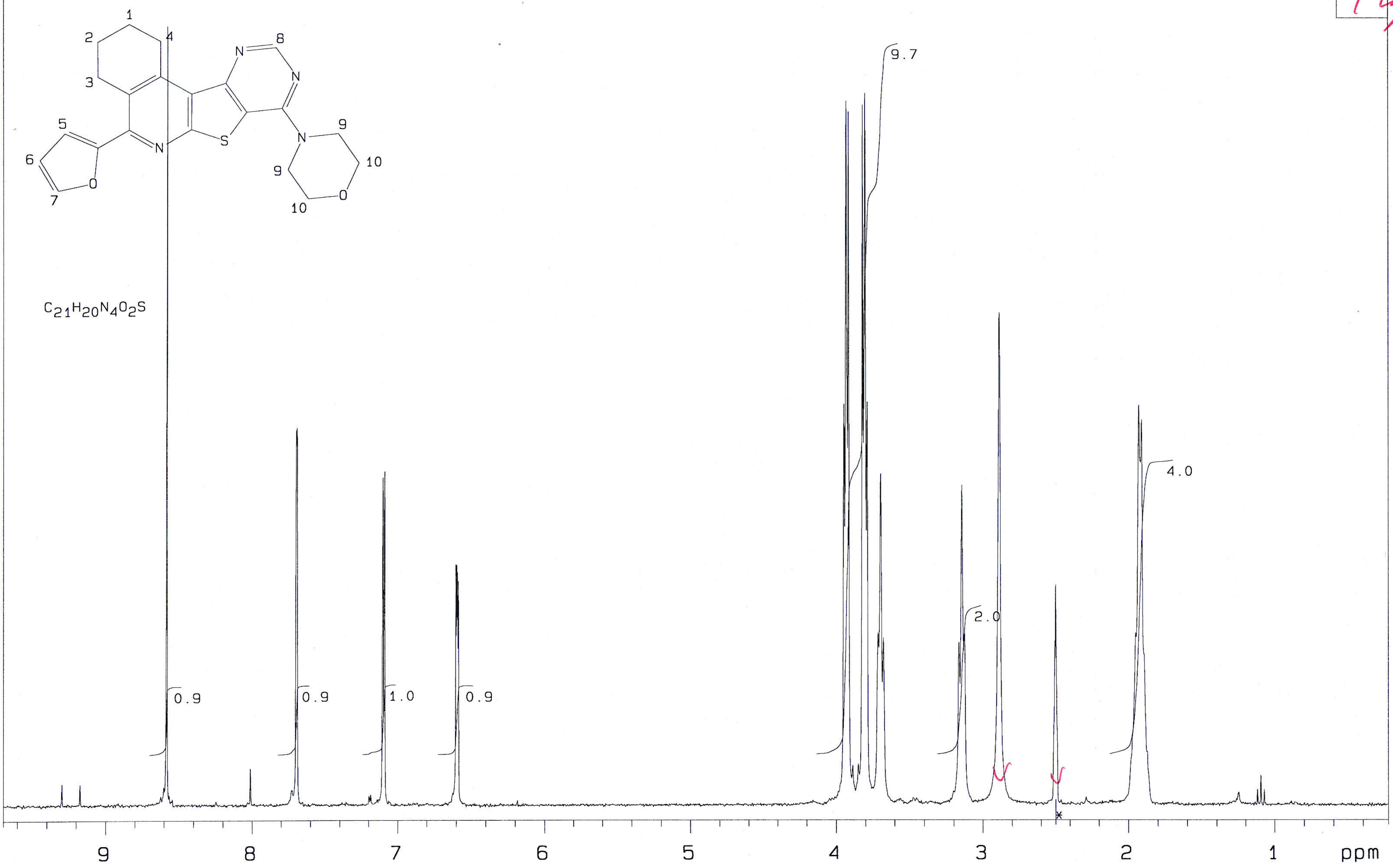

5c

Molecular Structure Research Centre, Yerevan, Armenia, Varian Mercury-300VX

C13 75.465 MHz, nt=832, np=19998, temp=30.0 C, lb=1.0, solvent=DMSO/CCl4 1/3

Jul 18 2019

AE99-199

SAMV_19 ae99-199

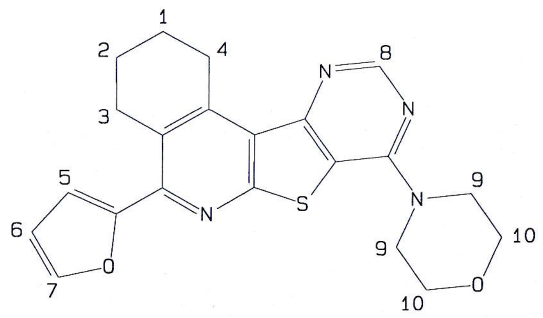

$C_{21}H_{20}N_4O_2S$

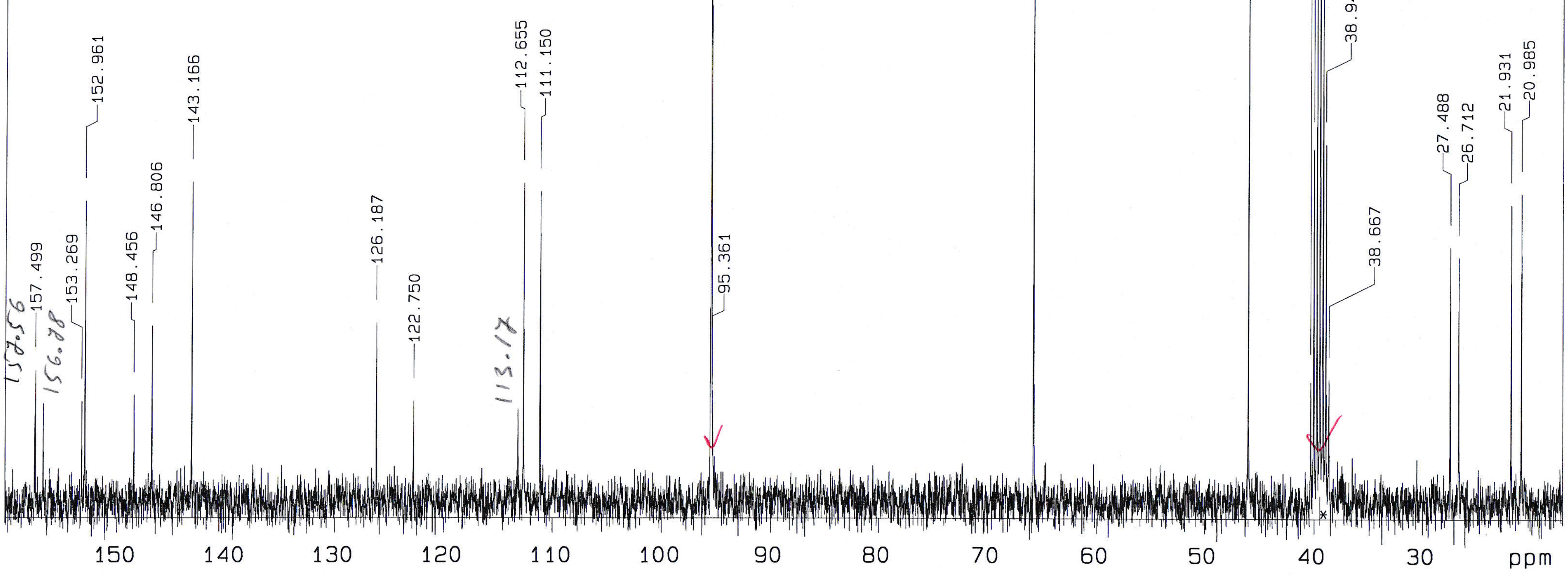

5d

Molecular Structure Research Centre, Yerevan, Armenia, Varian Mercury-300VX H1 300.088 MHz, nt = 16, np = 32000, temp = 30.0 C, lb = -0.2, solvent = DMSO/CCl4 1/3 **Jul 16 2019**

SAMV_19 ae99-205

AE99-205

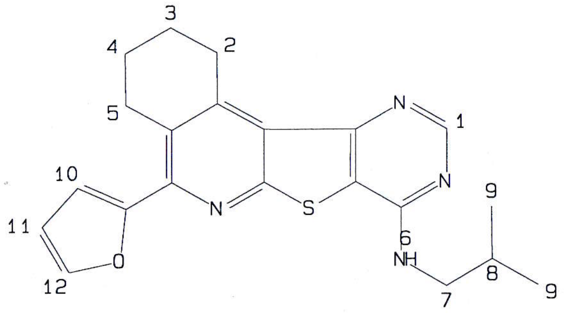

$C_{21}H_{22}N_4OS$

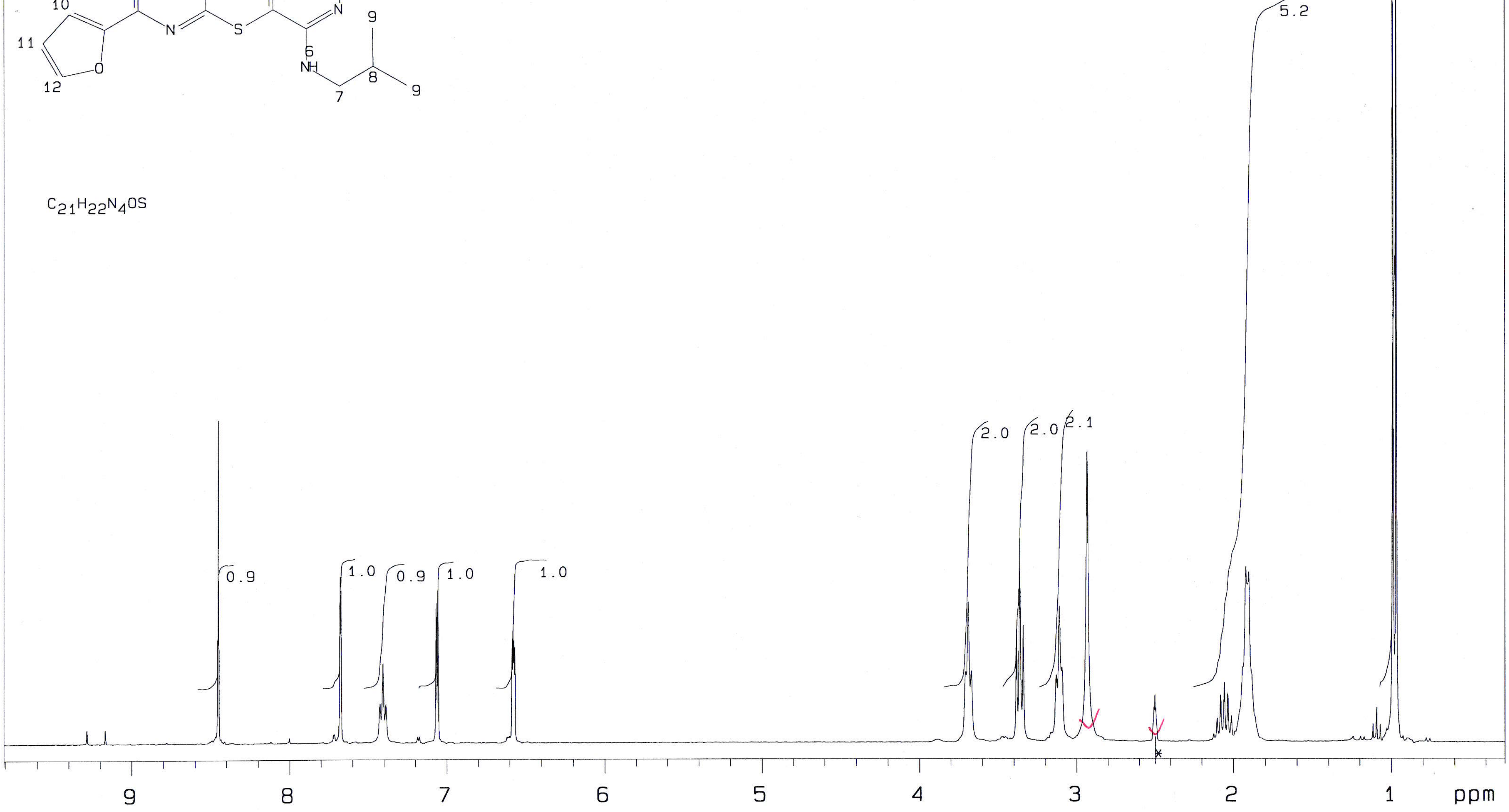

5d

Molecular Structure Research Centre, Yerevan, Armenia, Varian Mercury-300VX

C13 75.465 MHz, nt=608, np=19998, temp=30.0 C, lb=1.0, solvent=DMSO/CCl4 1/3

Jul 16 2019

SAMV_19 ae99-205

AE99-205

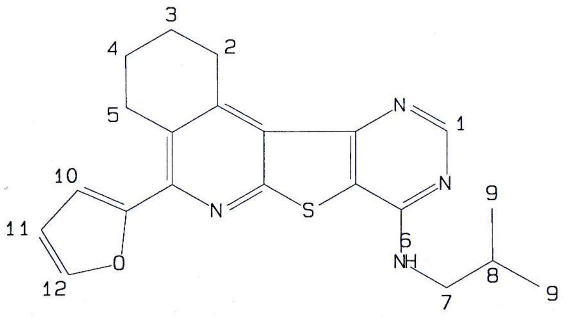

$C_{21}H_{22}N_4OS$

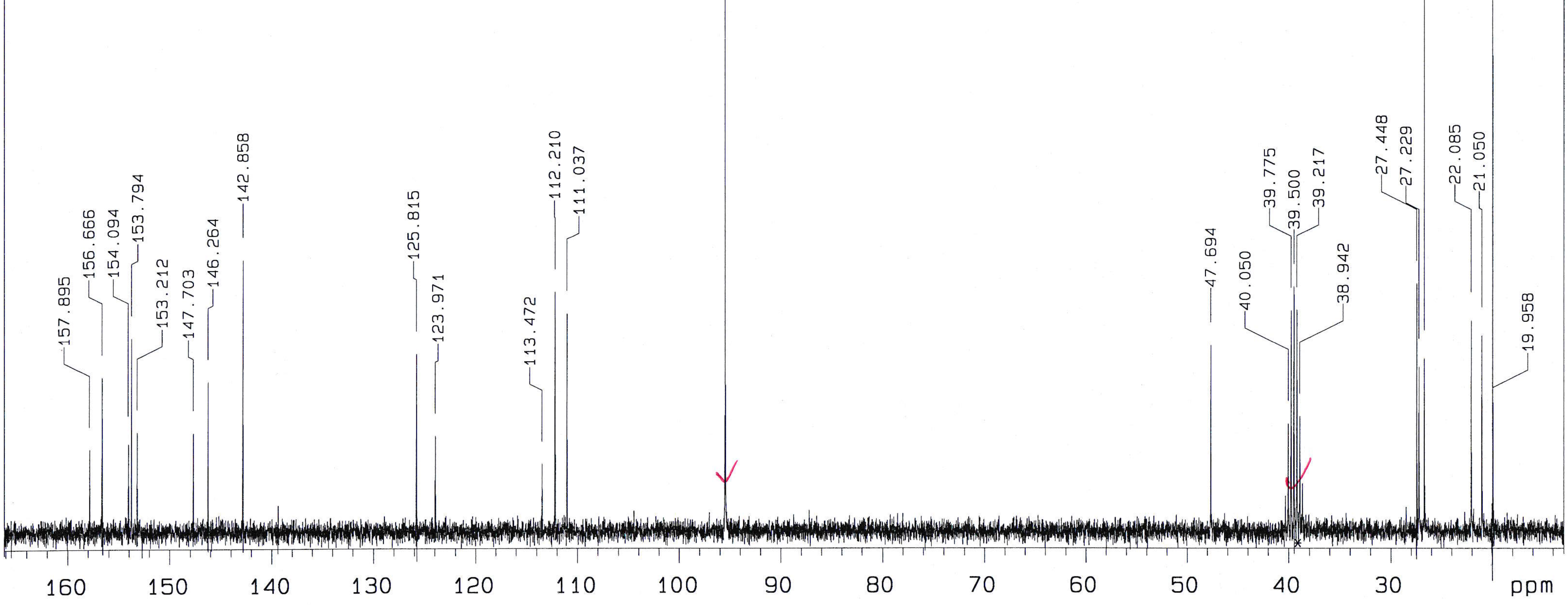

5e

Molecular Structure Research Centre, Yerevan, Armenia, Varian Mercury-300VX

H1 300.088 MHz, nt = 16, np = 32000, temp = 30.0 C, lb = -0.2, solvent = DMSO/CCl4 1/3

Jul 9 2019

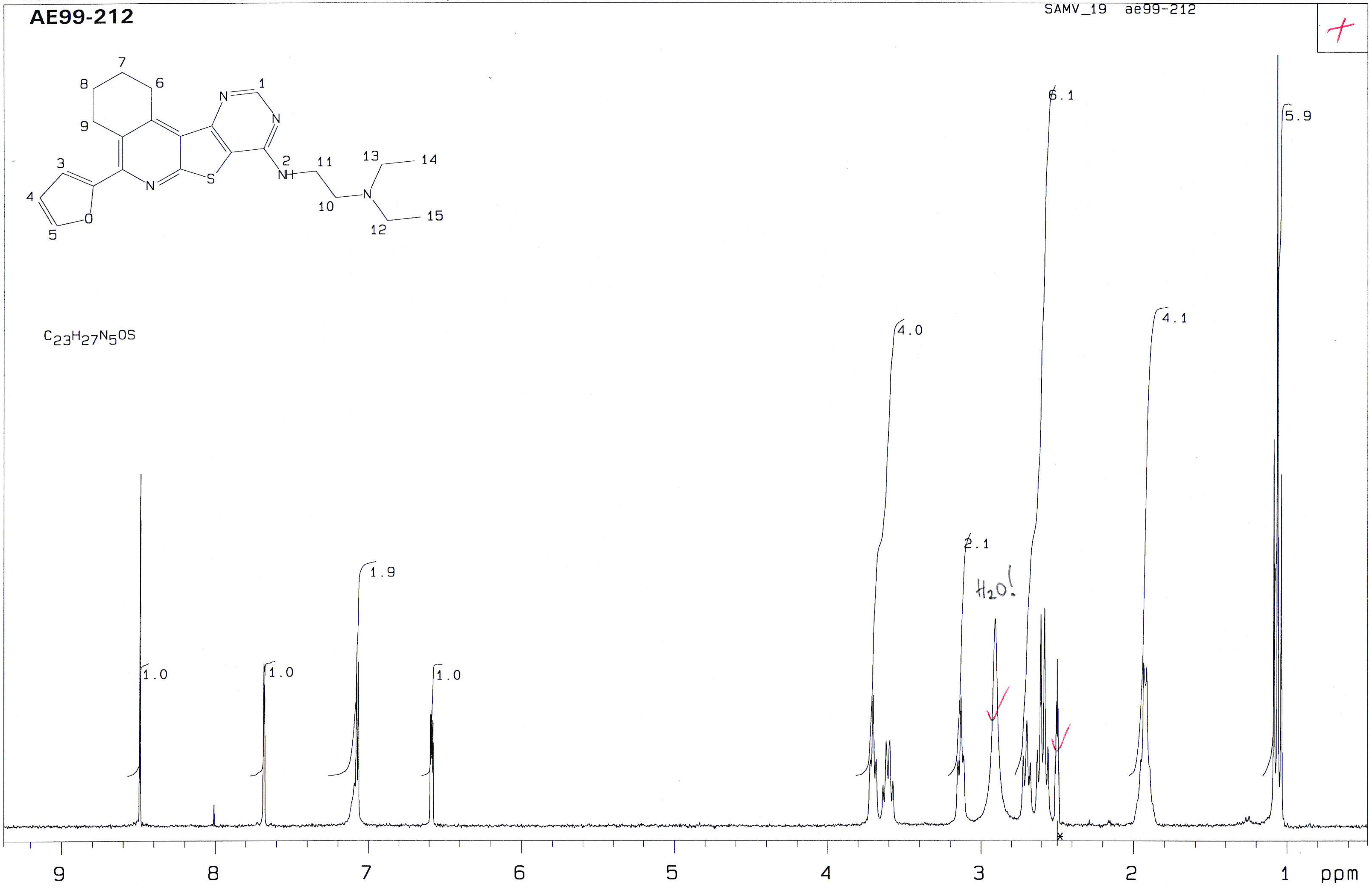

5e

Molecular Structure Research Centre, Yerevan, Armenia, Varian Mercury-300VX

C13 75.465 MHz, nt = 192, np = 19998, temp = 30.0 C, lb = 1.0, solvent = DMSO/CCl4 1/3

Jul 9 2019

SAMV_19 ae99-212

AE99-212

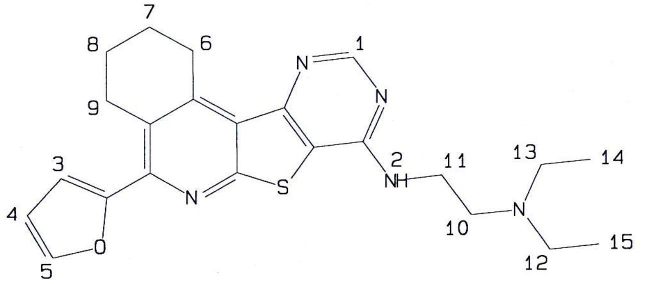

$C_{23}H_{27}N_5OS$

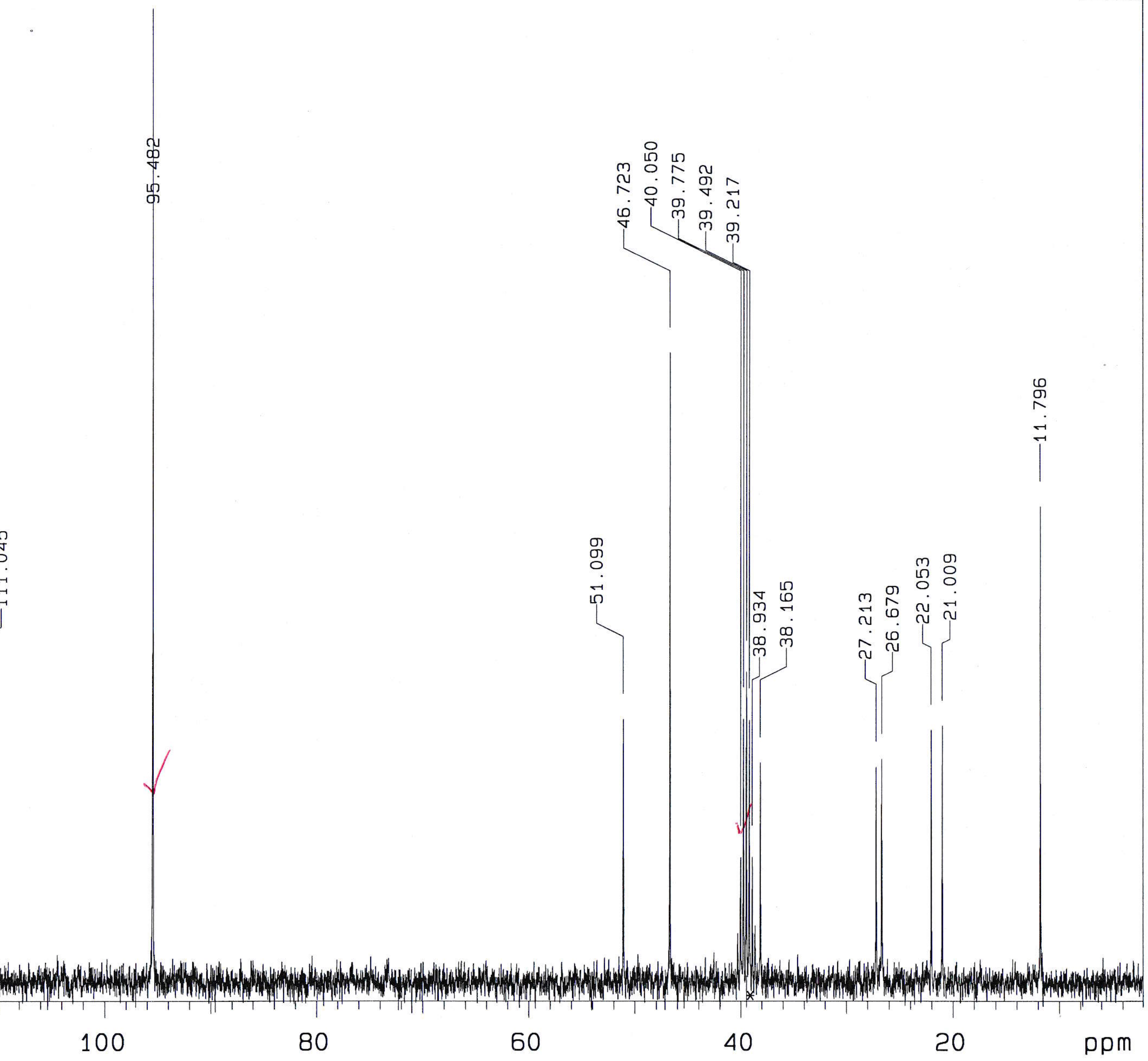

5f

Molecular Structure Research Centre, Yerevan, Armenia, Varian Mercury-300VX

H1 300.088 MHz, nt = 16, np = 32000, temp = 30.0 C, lb = -0.2, solvent = DMSO/CCl4 1/3

Jul 9 2019

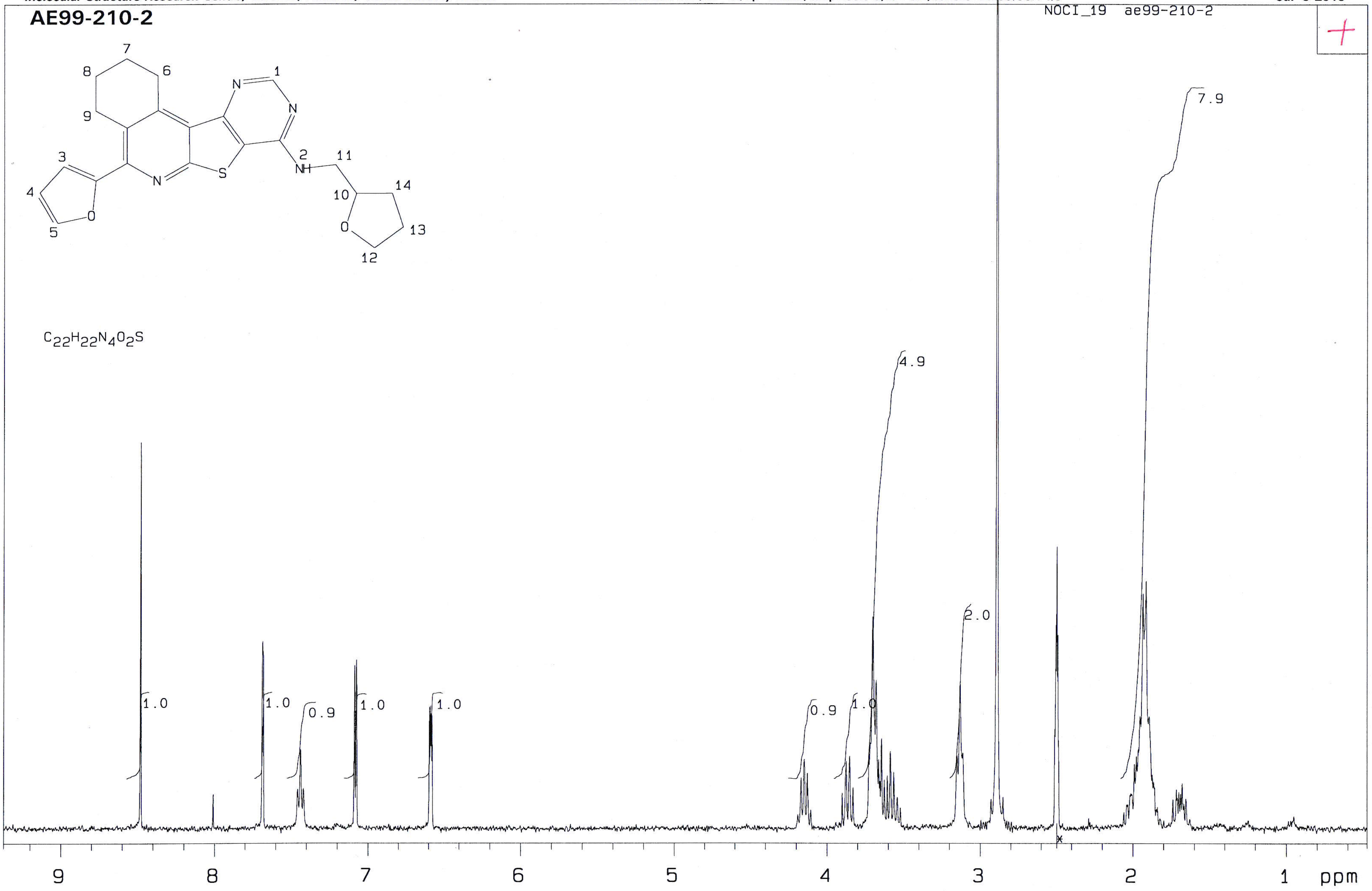

5f

Molecular Structure Research Centre, Yerevan, Armenia, Varian Mercury-300VX **C13 75.465 MHz, nt=672, np=19998, temp=30.0 C, lb=1.0, solvent=DMSO/CCl4 1/3** **Jul 9 2019**

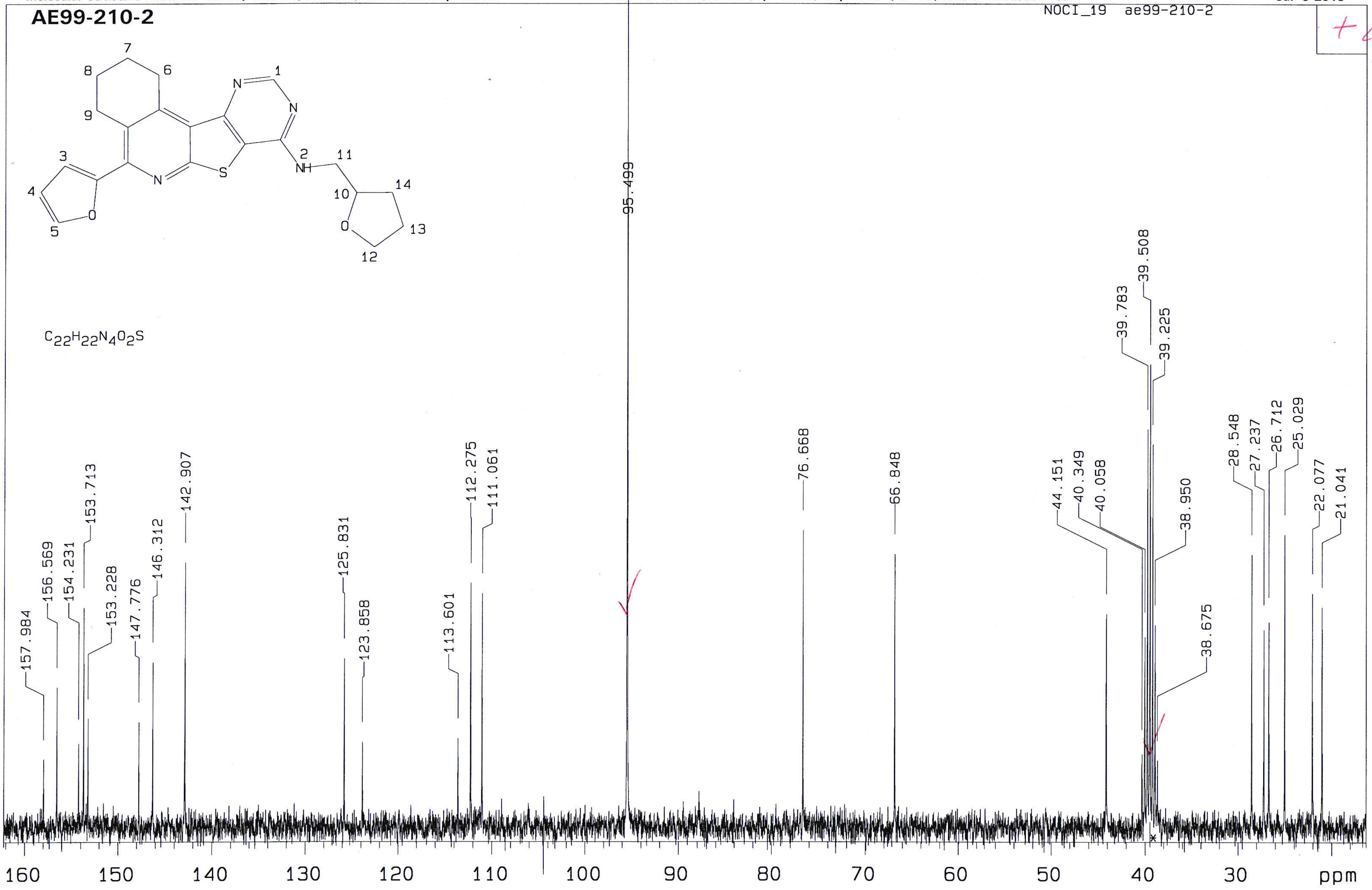

7c

Molecular Structure Research Centre, Yerevan, Armenia, Varian Mercury-300VX H1 300.088 MHz, nt = 16, np = 32000, temp = 30.0 C, lb = -0.2, solvent = DMSO/CCl4 1/3 Jul 2 2019

HA-119

SAMV_19 ha-119

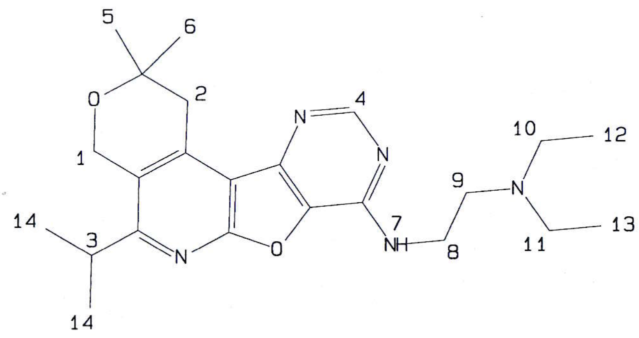

$C_{23}H_{33}N_5O_2$

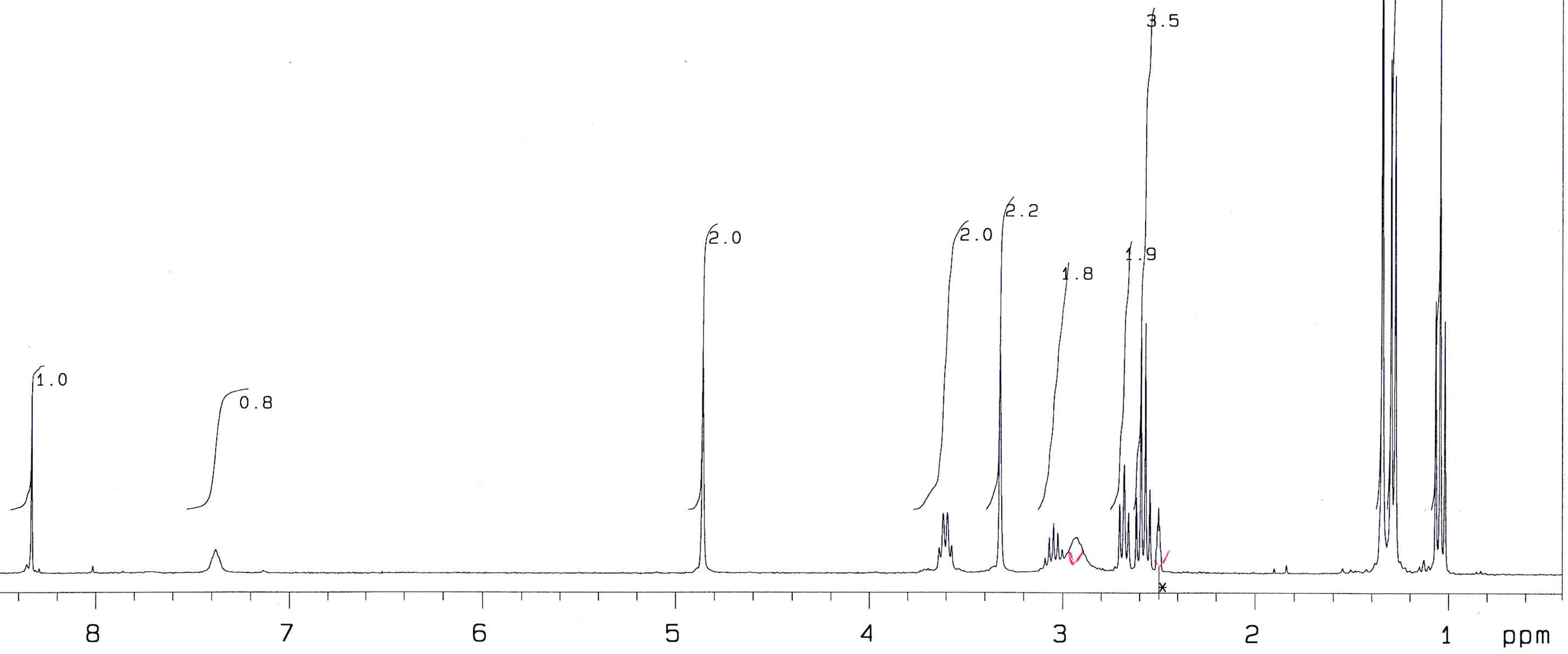

7c

Molecular Structure Research Centre, Yerevan, Armenia, Varian Mercury-300VX

C13 75.465 MHz, nt = 1040, np = 19998, temp = 30.0 C, lb = 1.0, solvent = DMSO/CCl4 1/3

Jul 2 2019

SAMV_19 ha-119

HA-119

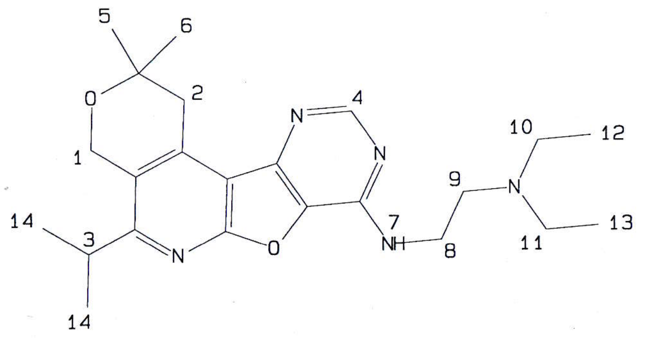

$C_{23}H_{33}N_5O_2$

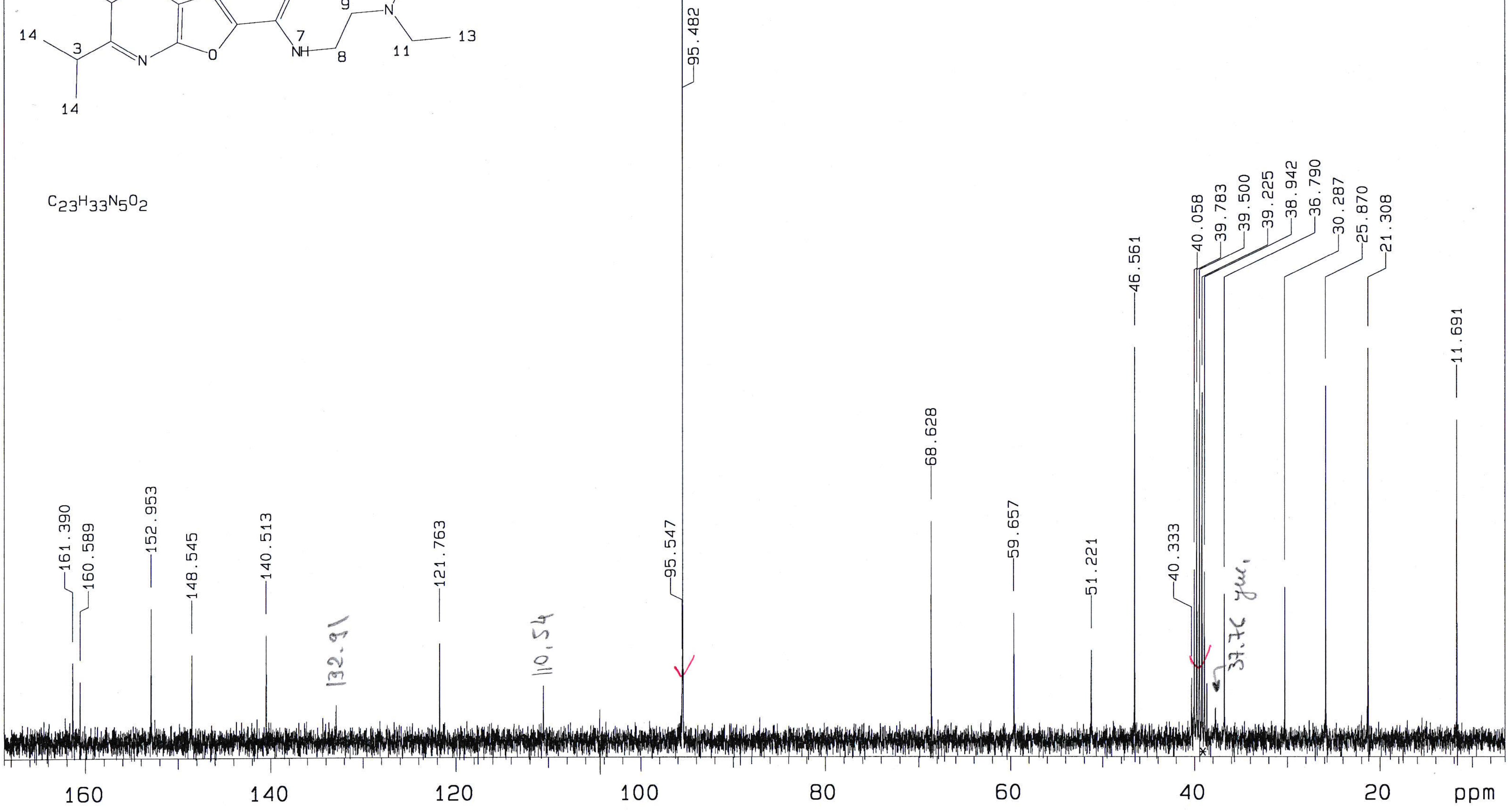

7e

Molecular Structure Research Centre, Yerevan, Armenia, Varian Mercury-300VX

H1 300.088 MHz, nt=16, np=32000, temp=30.0 C, lb=-0.2, solvent=DMSO/CCl4 1/3

Jul 4 2019

AE0012-0361

SAMV_19 ae0012-0361

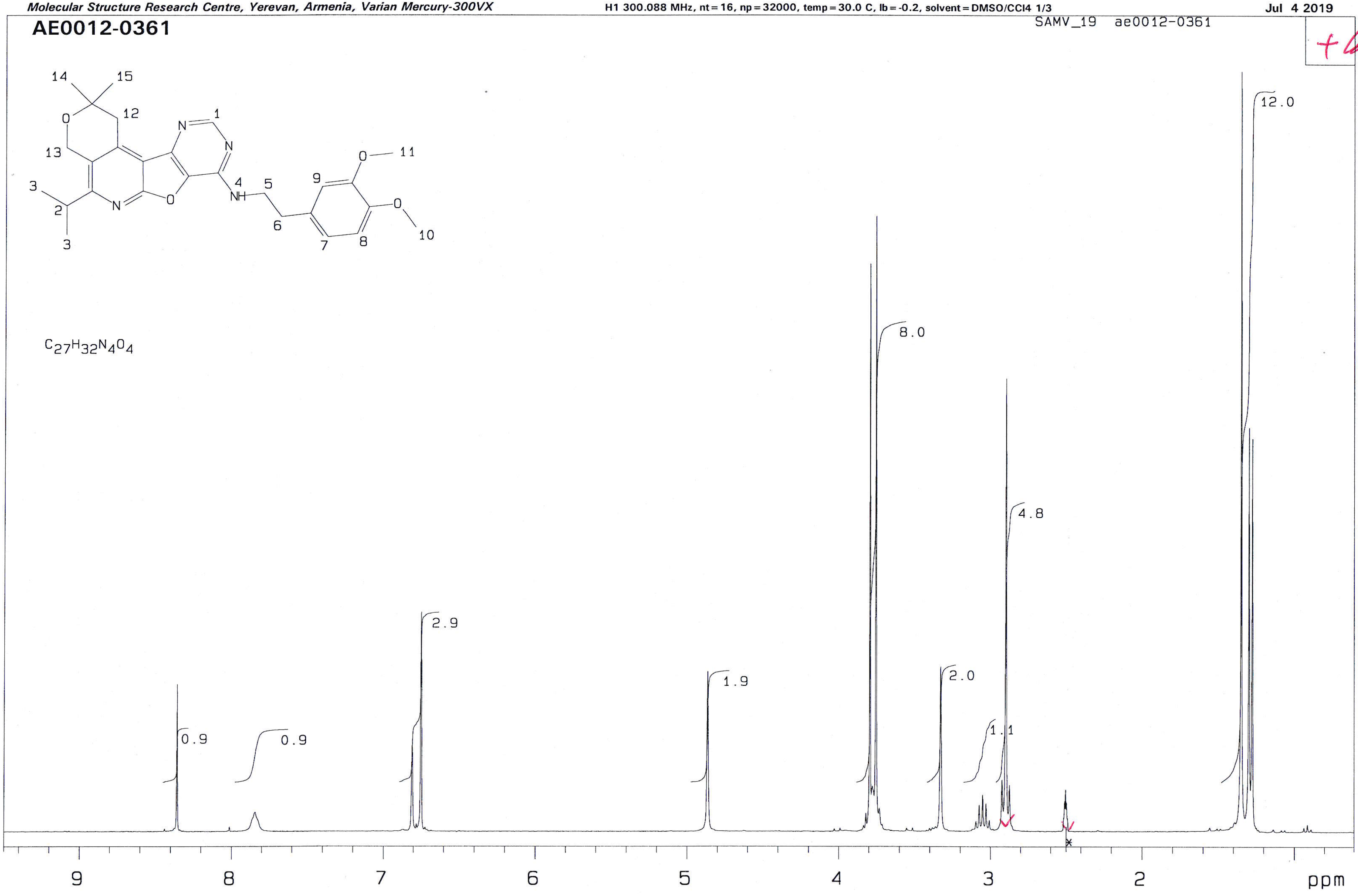

7e

Molecular Structure Research Centre, Yerevan, Armenia, Varian Mercury-300VX

C13 75.465 MHz, nt=5392, np=19998, temp=30.0 C, lb=1.0, solvent=DMSO/CCl4 1/3

Jul 4 2019

SAMV_19 ae0012-0361

AE0012-0361

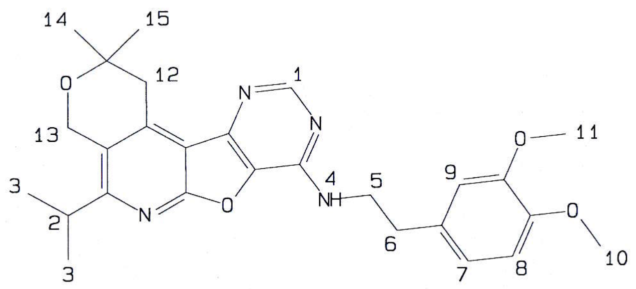

$C_{27}H_{32}N_4O_4$

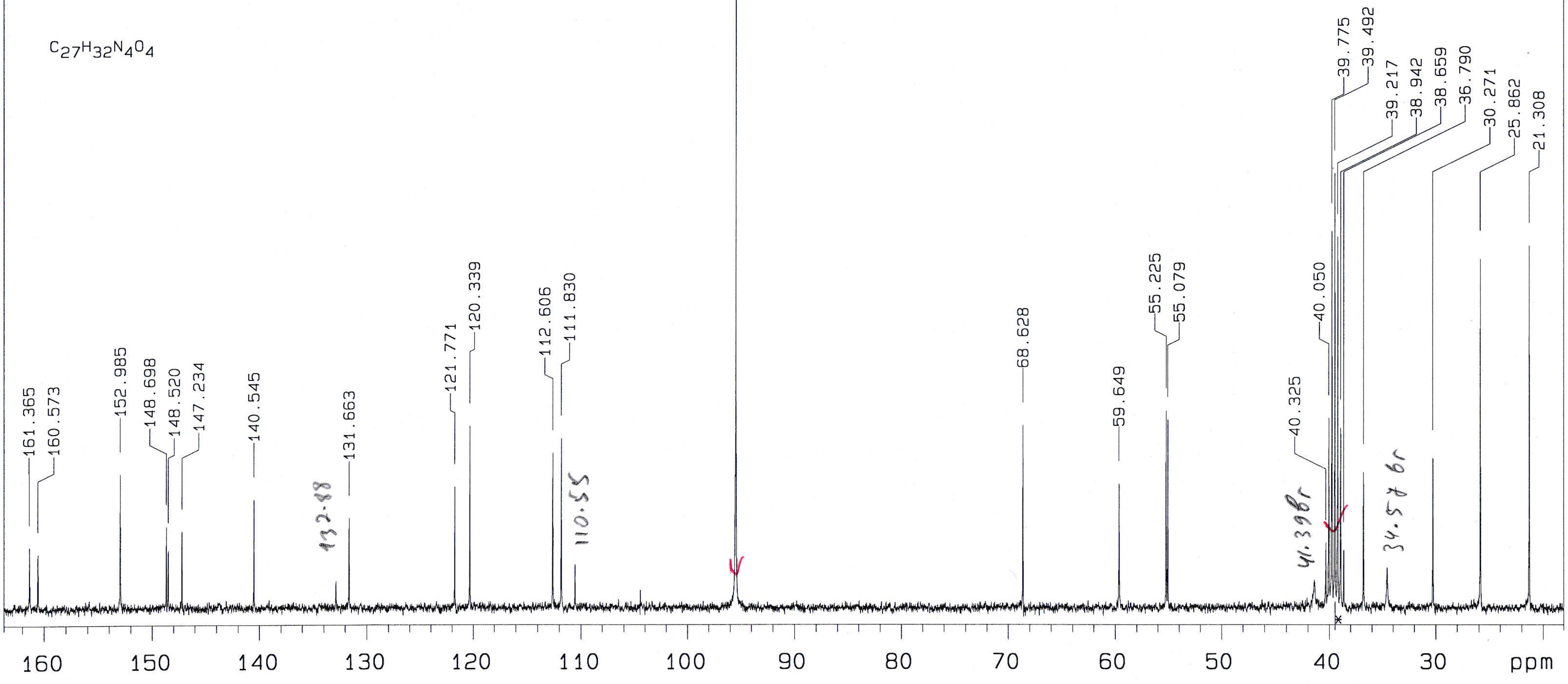

7f

Molecular Structure Research Centre, Yerevan, Armenia, Varian Mercury-300VX

H1 300.088 MHz, nt = 16, np = 32000, temp = 30.0 C, lb = -0.2, solvent = DMSO/CCl4 1/3

Jul 3 2019

HA-392

SAMV_19 ha-392

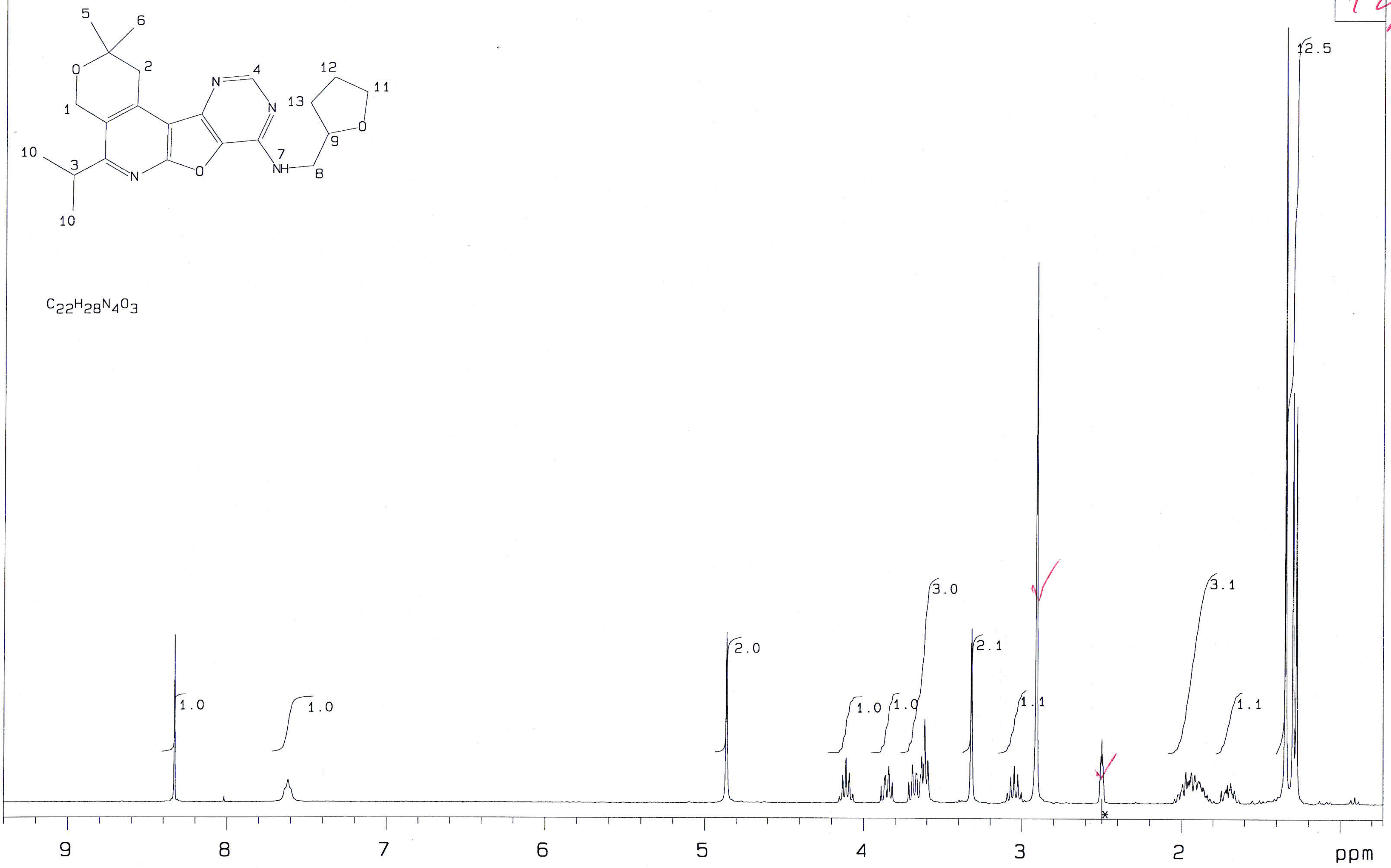

7f

Molecular Structure Research Centre, Yerevan, Armenia, Varian Mercury-300VX C13 75.465 MHz, nt = 1888, np = 19998, temp = 30.0 C, lb = 1.0, solvent = DMSO/CCl4 1/3 **Jul 3 2019**

HA-392

SAMV_19 ha-392

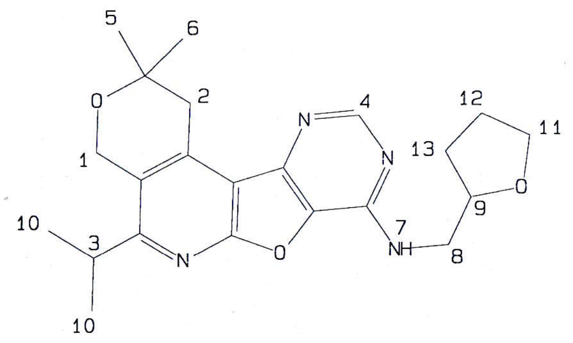

$C_{22}H_{28}N_4O_3$

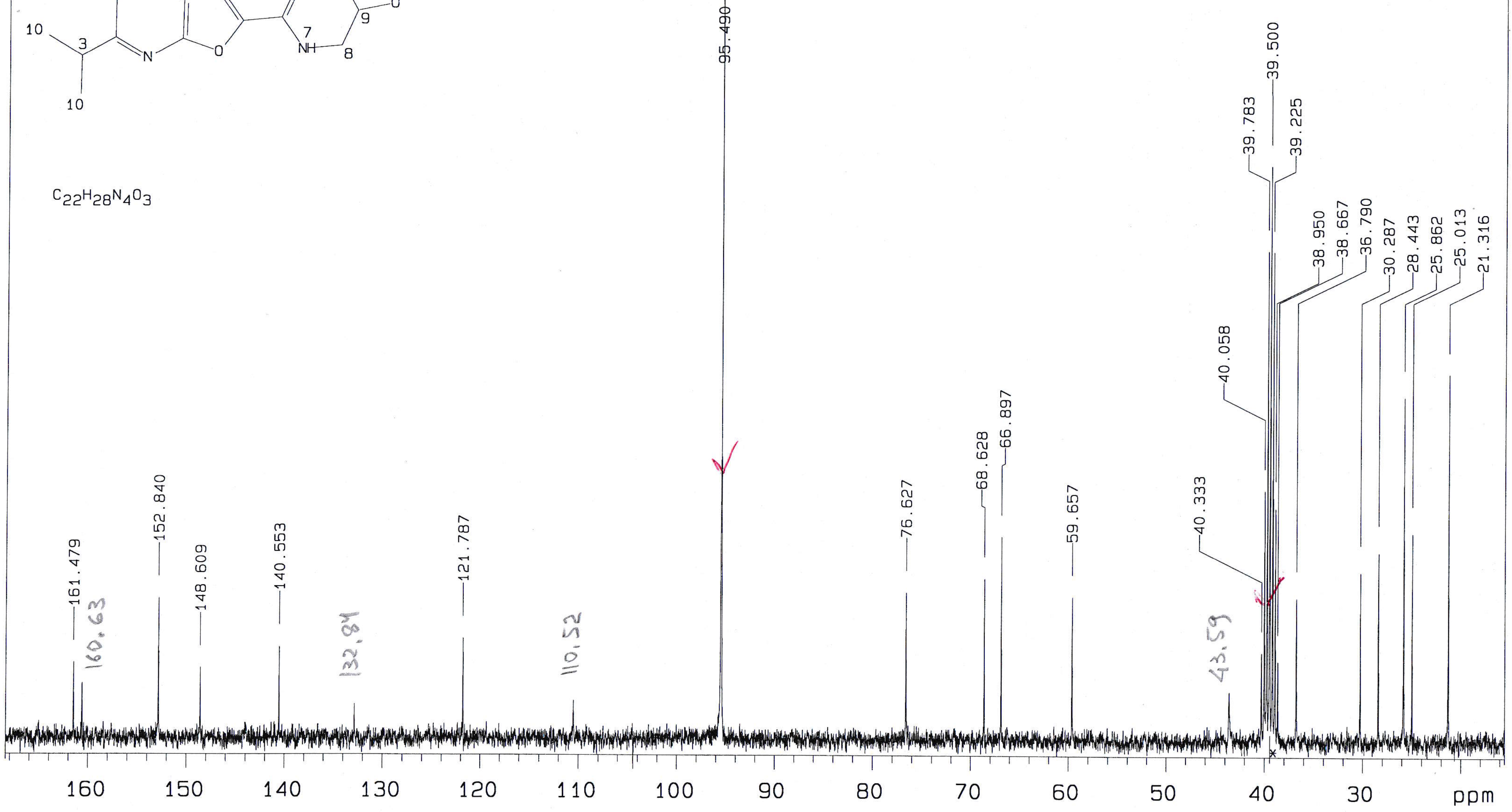

7q

Molecular Structure Research Centre, Yerevan, Armenia, Varian Mercury-300VX

H1 300.088 MHz, nt=16, np=32000, temp=30.0 C, lb=-0.2, solvent=DMSO/CCl4 1/3

Jul 2 2019

SAMV_19 ~~ha-439~~

HA-439

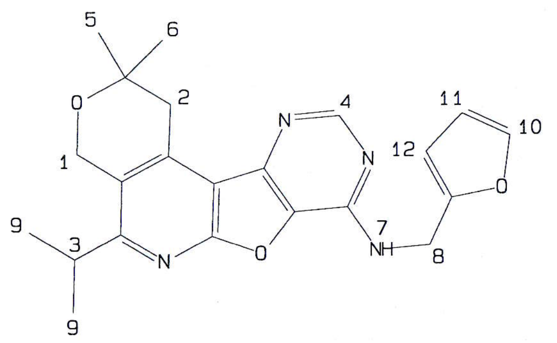

$C_{22}H_{24}N_4O_3$

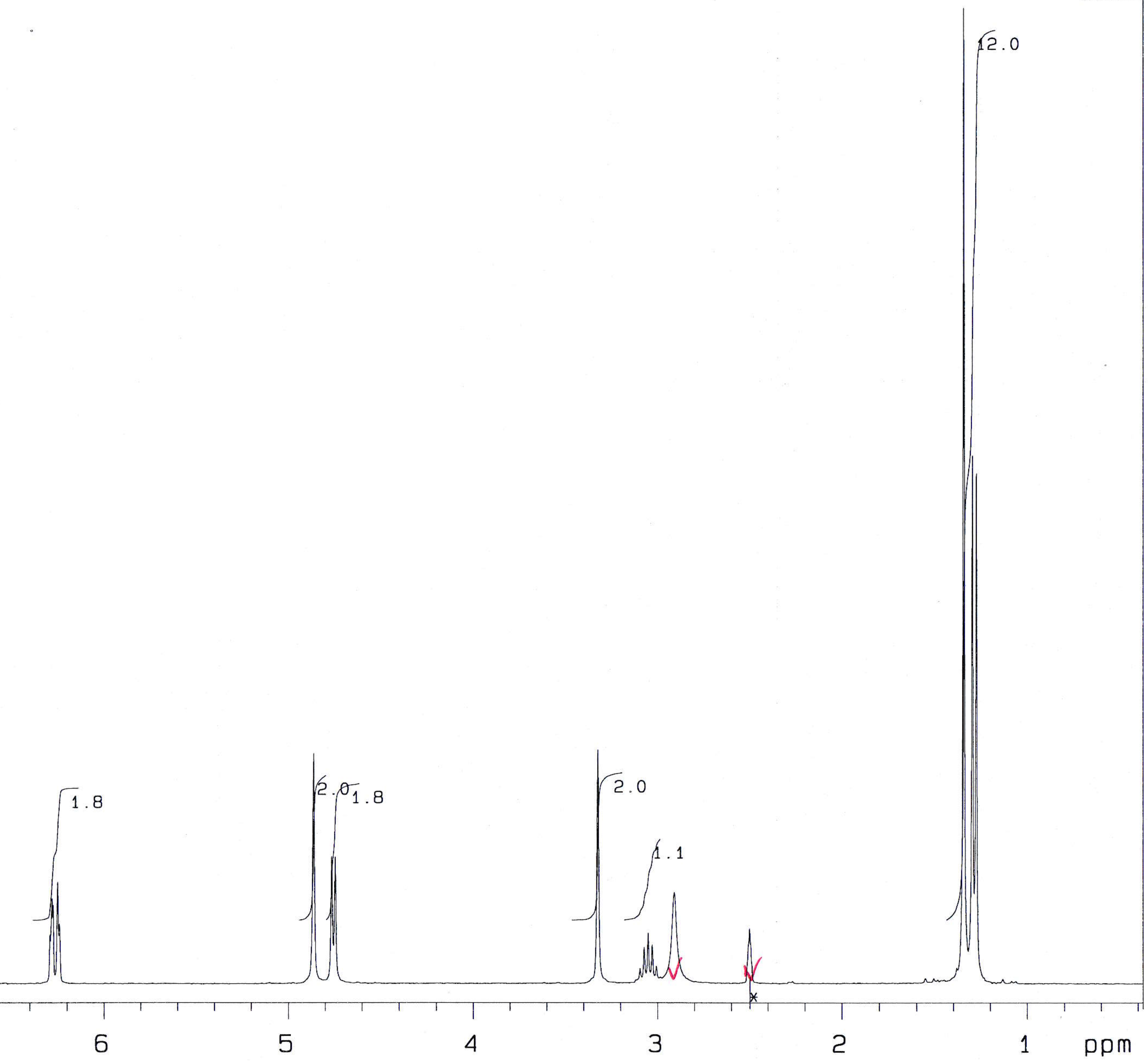

7g

Molecular Structure Research Centre, Yerevan, Armenia, Varian Mercury-300VX

C13 75.465 MHz, nt=976, np=19998, temp=30.0 C, lb=1.0, solvent=DMSO/CCl4 1/3

Jul 3 2019

SAMV_19 ae0012-0320

AE0012-0320

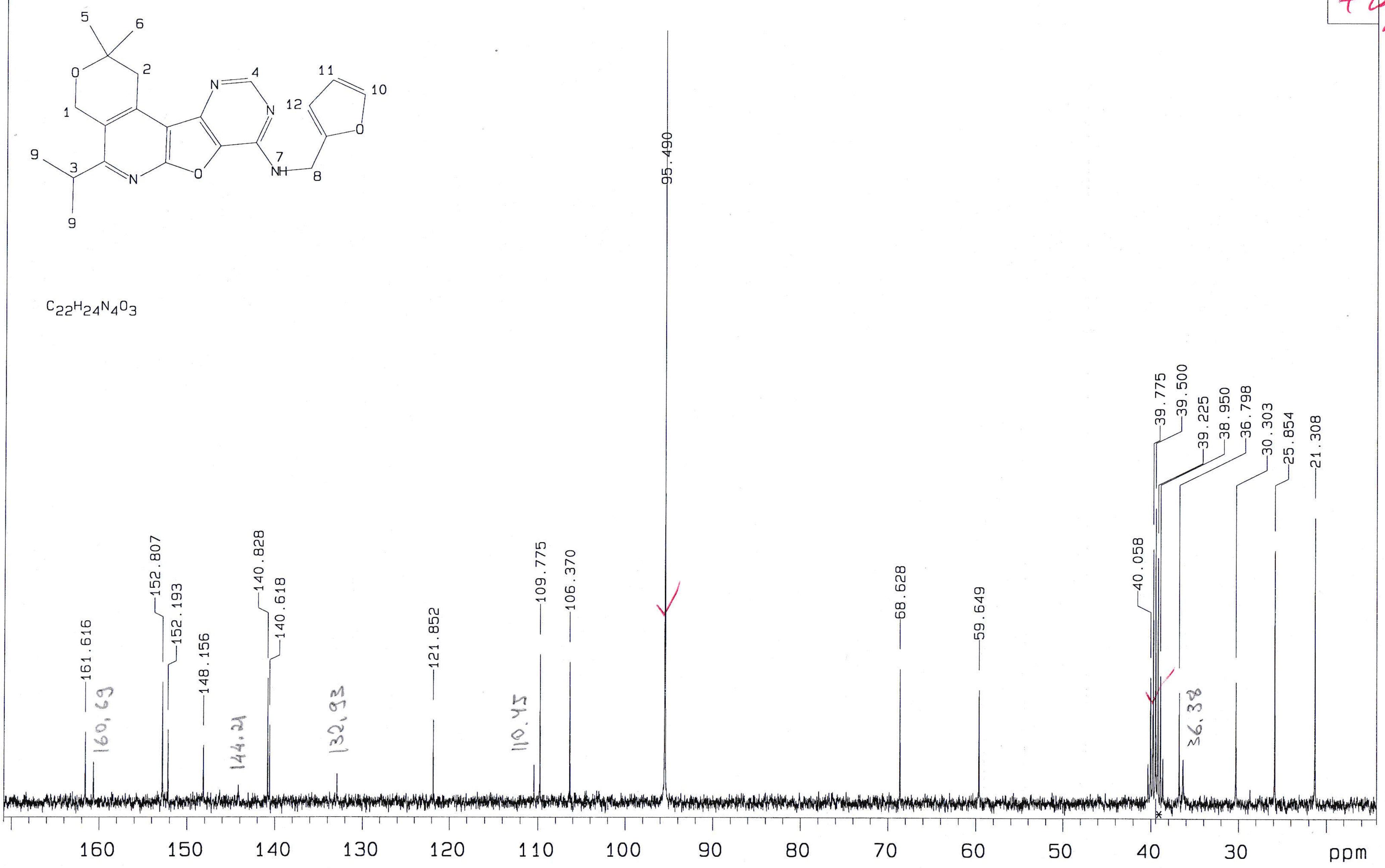

7j

Molecular Structure Research Centre, Yerevan, Armenia, Varian Mercury-300VX **H1 300.088 MHz, nt = 16, np = 32000, temp = 30.0 C, lb = -0.2, solvent = DMSO/CCl4 1/3** **Jul 22 2019**

AE0012-0438

SAMV_19 ae0012-0438

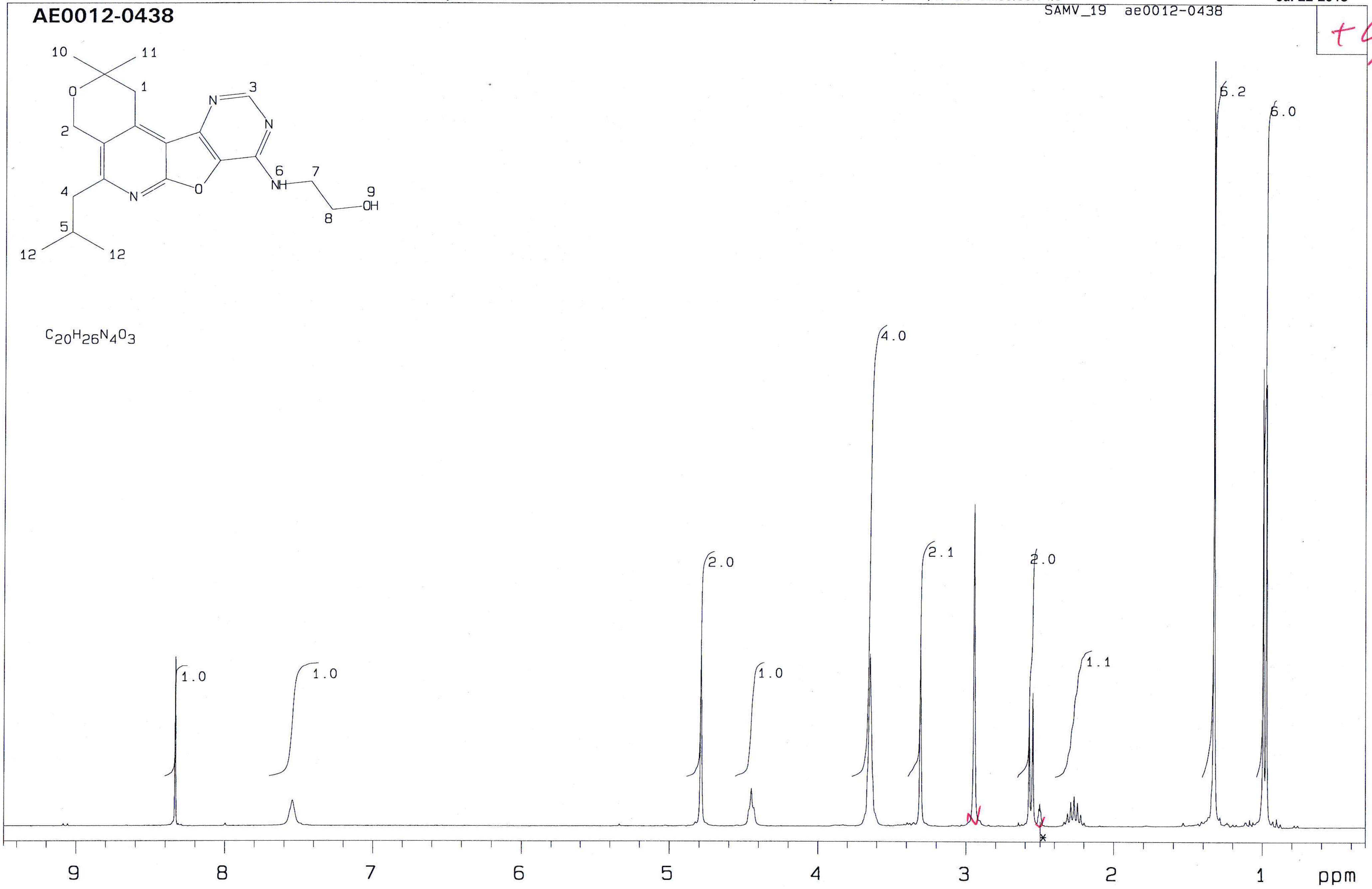

7j

Molecular Structure Research Centre, Yerevan, Armenia, Varian Mercury-300VX **C13 75.465 MHz, nt = 1504, np = 19998, temp = 30.0 C, lb = 1.0, solvent = DMSO/CCl4 1/3** **Jul 22 2019**

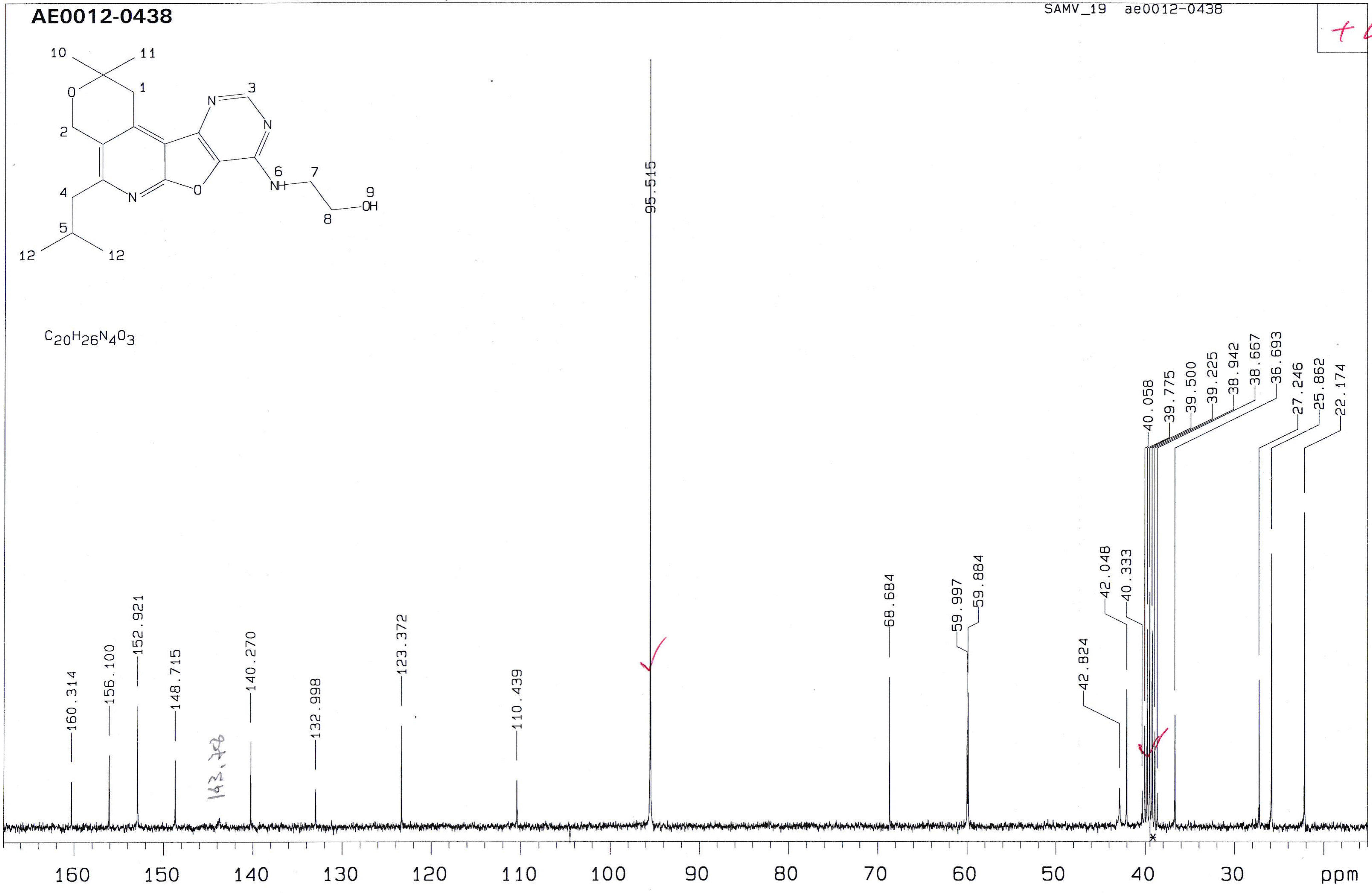

7e

Molecular Structure Research Centre, Yerevan, Armenia, Varian Mercury-300VX H1 300.088 MHz, nt=16, np=32000, temp=30.0 C, lb=-0.2, solvent=DMSO/CCl4 1/3 **Jul 8 2019**

SC-0318-1217

SAMV_19 sc-0318-1217

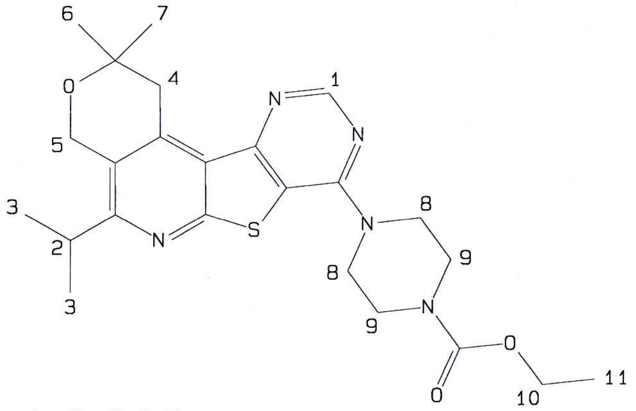

$C_{24}H_{31}N_5O_3S$

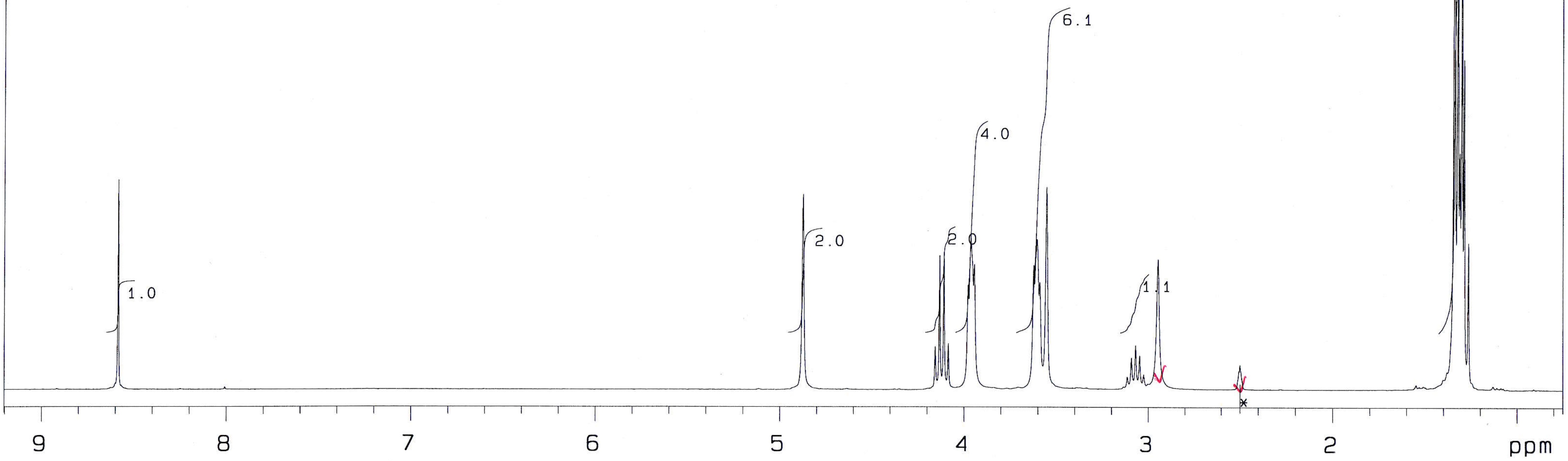

7e

Molecular Structure Research Centre, Yerevan, Armenia, Varian Mercury-300VX

C13 75.465 MHz, nt=528, np=19998, temp=30.0 C, lb=1.0, solvent=DMSO/CCl4 1/3

Jul 8 2019

SAMV_19 sc-0318-1217

SC-0318-1217

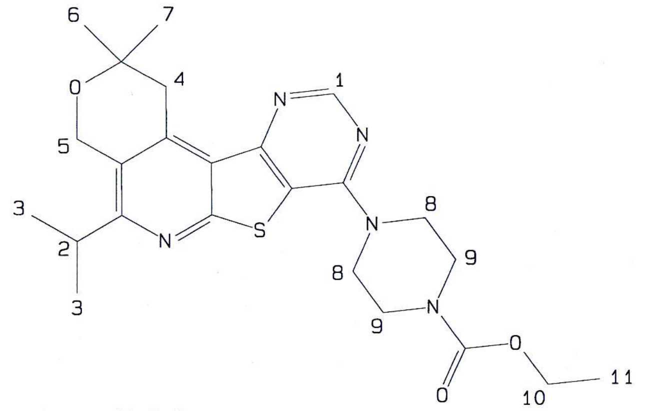

$C_{24}H_{31}N_5O_3S$

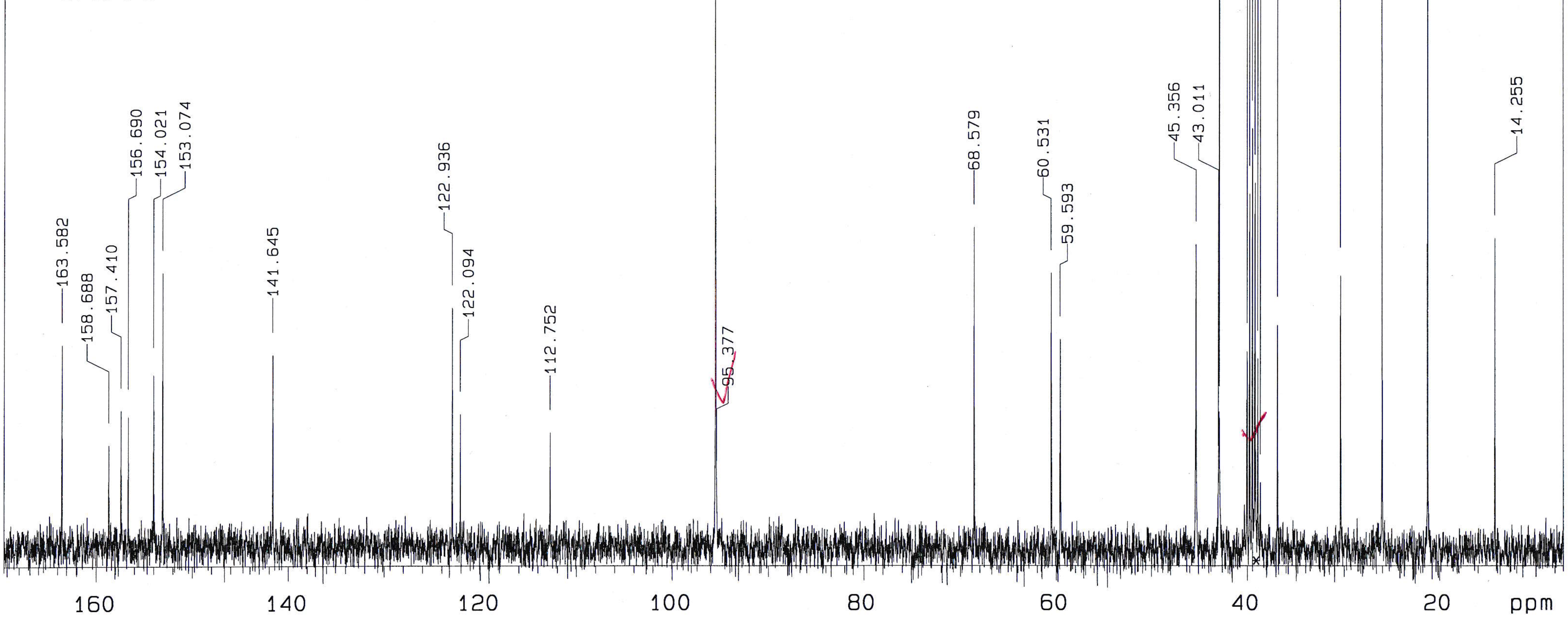

7m

Molecular Structure Research Centre, Yerevan, Armenia, Varian Mercury-300VX **H1 300.088 MHz, nt = 16, np = 16000, temp = 30.0 C, lb = 1.0, solvent = DMSO/CCl4 1/3** **Jul 15 2019**

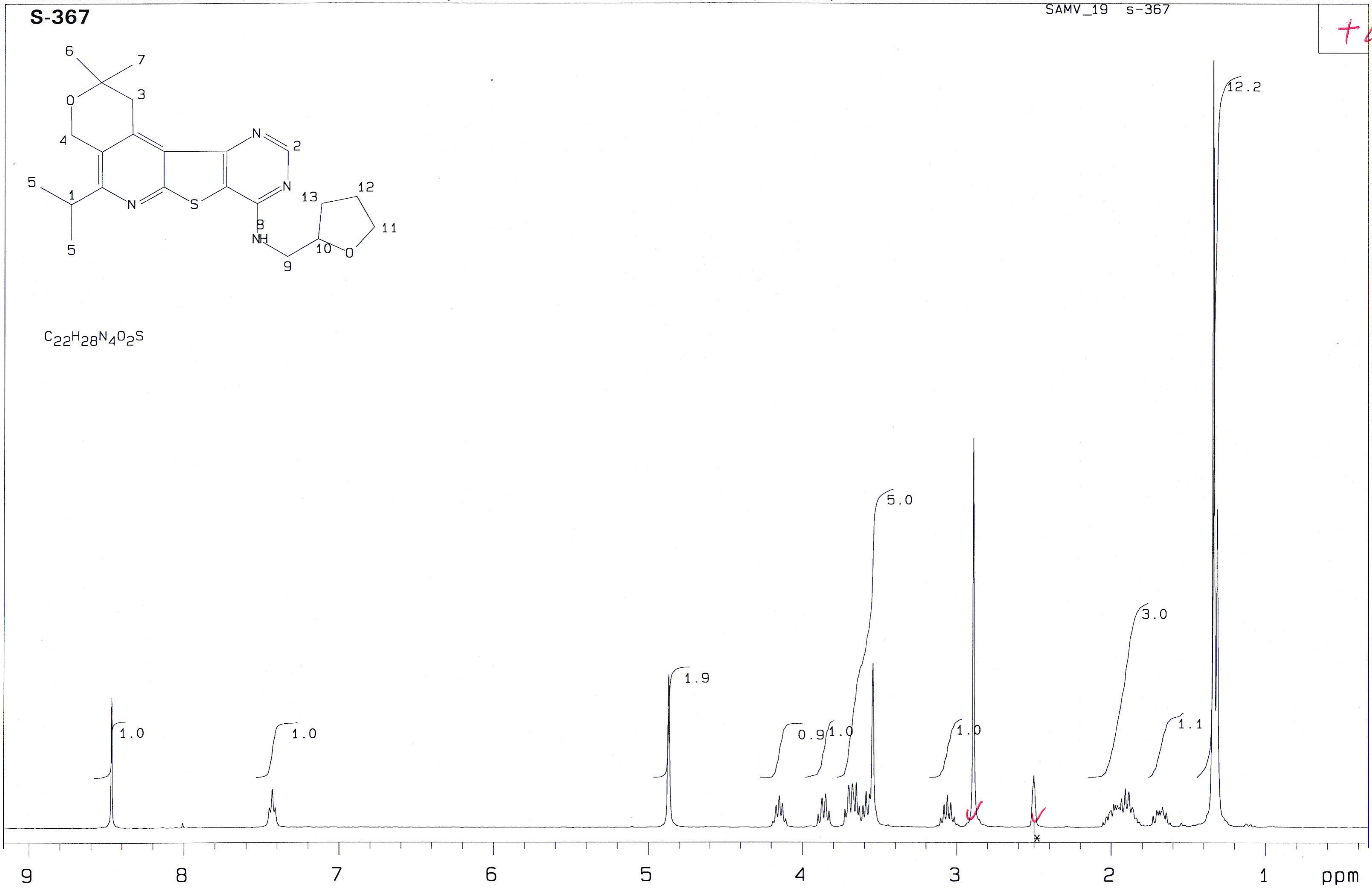

7m

Molecular Structure Research Centre, Yerevan, Armenia, Varian Mercury-300VX

C13 75.465 MHz, nt=576, np=19998, temp=30.0 C, lb=1.0, solvent=DMSO/CCl4 1/3

Jul 15 2019

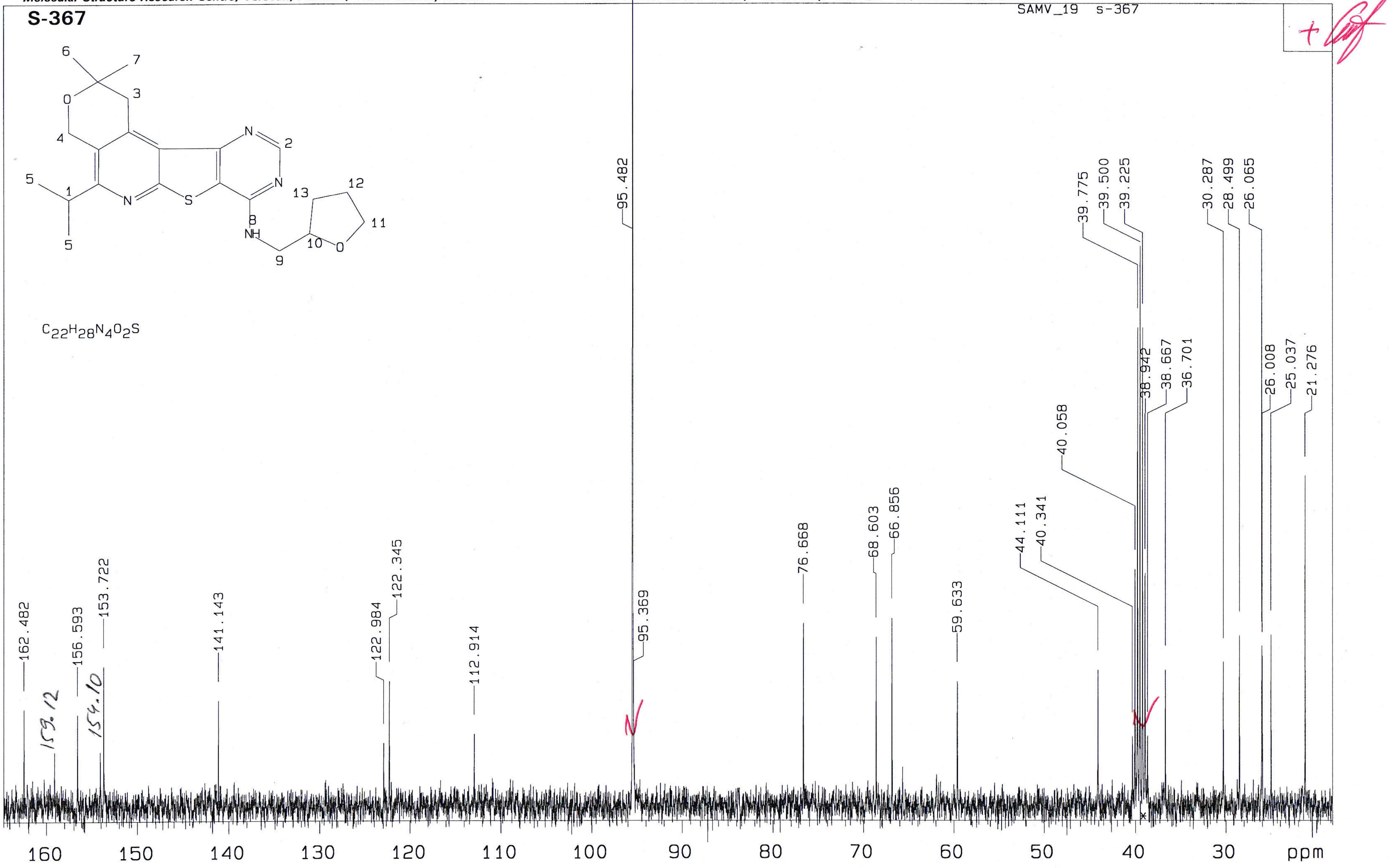

8

Molecular Structure Research Centre, Yerevan, Armenia, Varian Mercury-300VX

H1 300.077 MHz, nt = 16, np = 16000, temp = 30.0 C, lb = 1.0, solvent = DMSO/CCl4 1/3

Nov 3 2010

SAMV_10 ha-190

HA-190

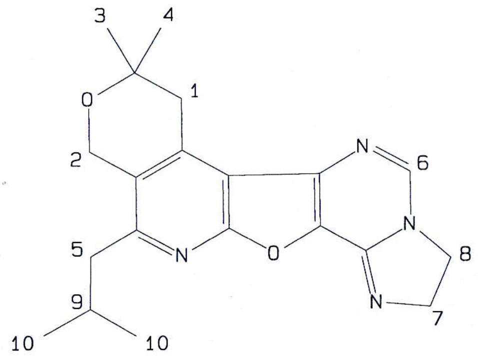

$C_{20}H_{24}N_4O_2$

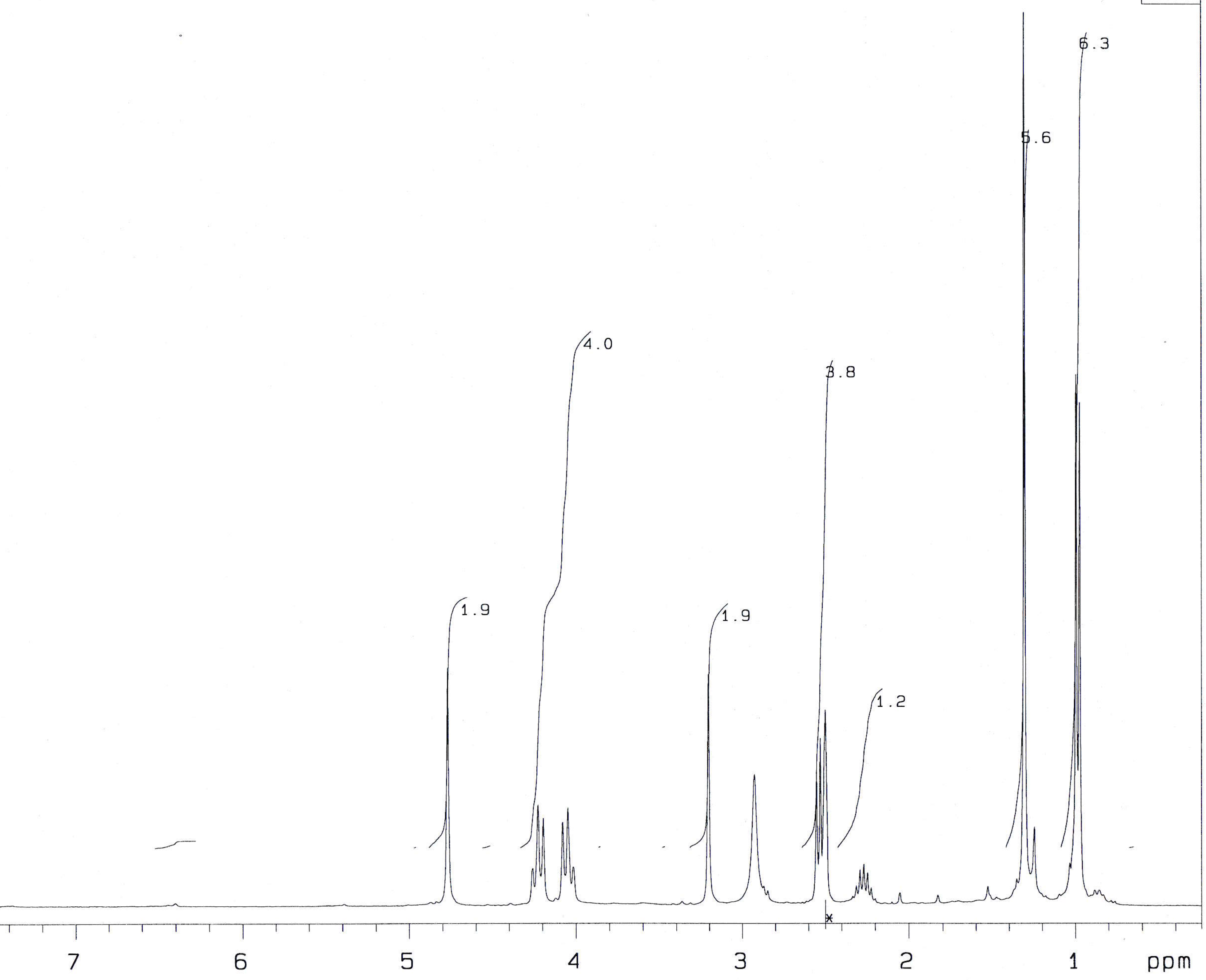

8

Molecular Structure Research Centre, Yerevan, Armenia, Varian Mercury-300VX

C13 75.465 MHz, nt = 1888, np = 19998, temp = 30.0 C, lb = 1.0, solvent = DMSO/CCl4 1/3

Jul 15 2019

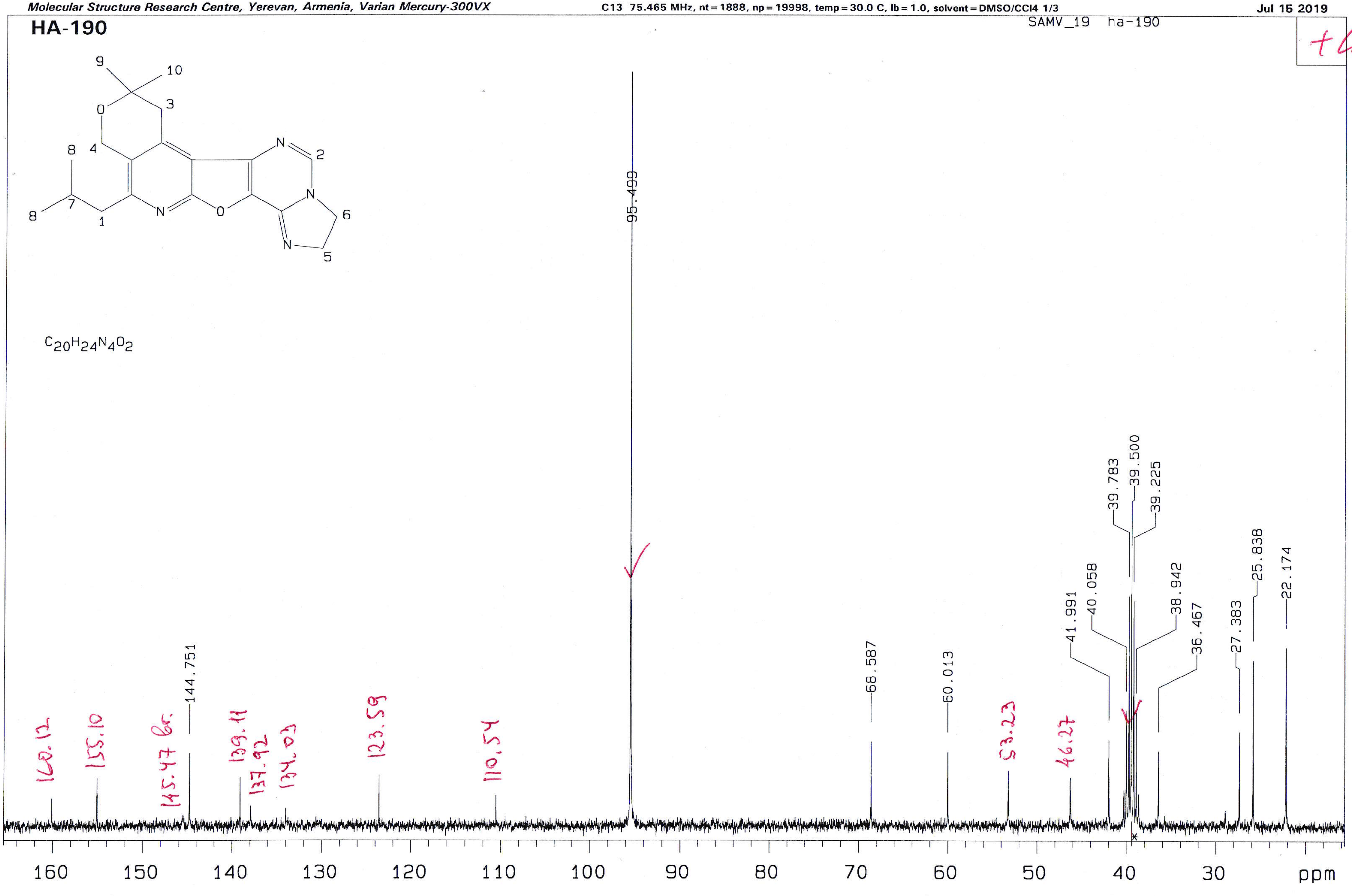

9

Molecular Structure Research Centre, Yerevan, Armenia, Varian Mercury-300VX

H1 300.088 MHz, nt = 16, np = 16000, temp = 30.0 C, lb = -0.2, solvent = DMSO/CCl4 1/3

Jul 18 2019

SAMV_19 ha-663

HA-663

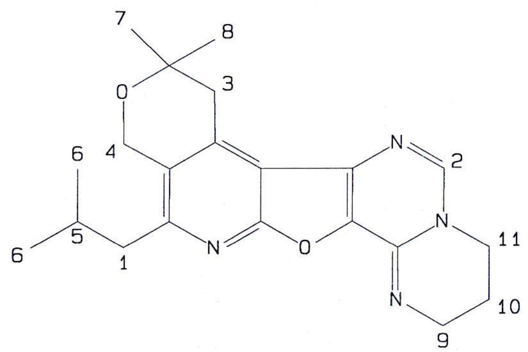

$C_{21}H_{26}N_4O_2$

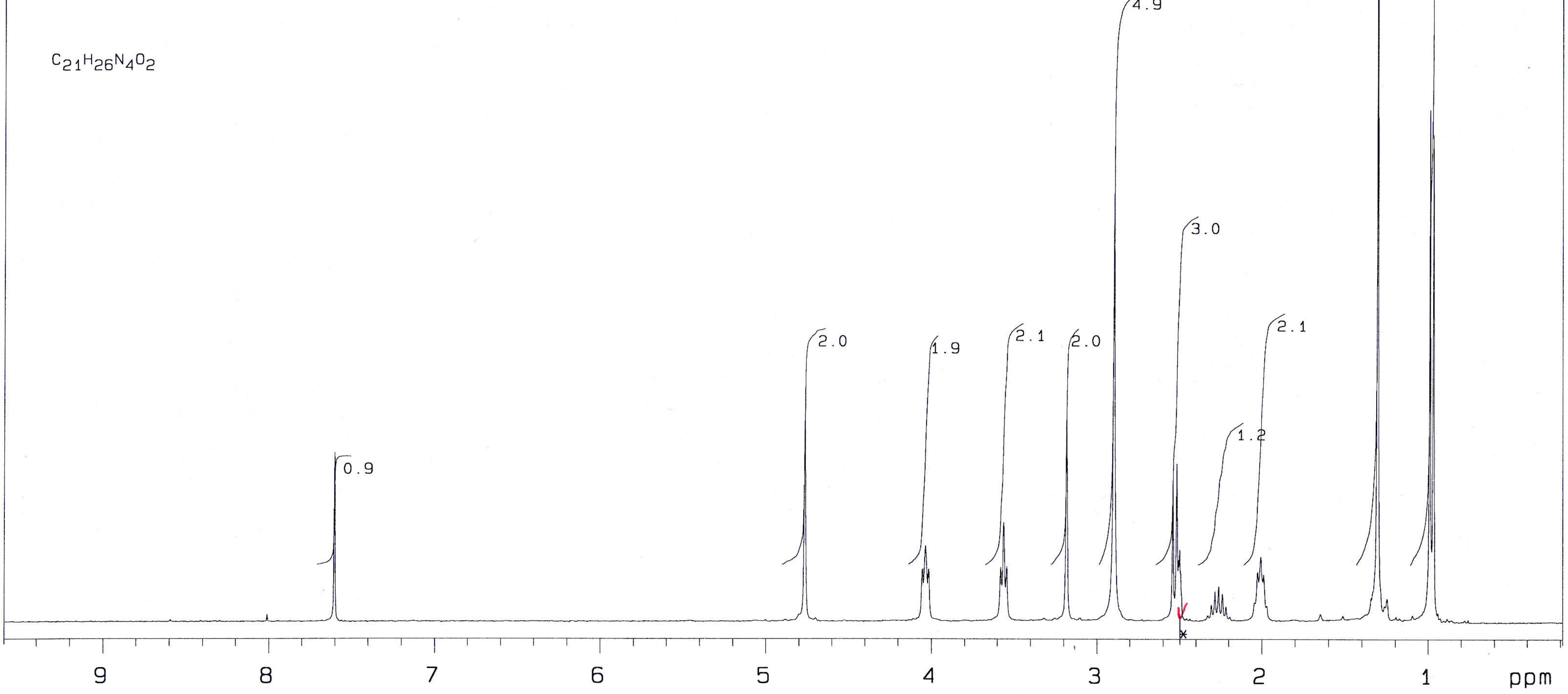

9

Molecular Structure Research Centre, Yerevan, Armenia, Varian Mercury-300VX

C13 75.465 MHz, nt = 2400, np = 19998, temp = 30.0 C, lb = 1.0, solvent = DMSO/CCl4 1/3

Jul 18 2019

SAMV_19 ha-663

HA-663

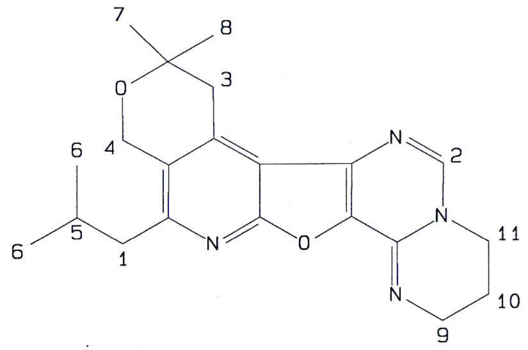

$C_{21}H_{26}N_4O_2$

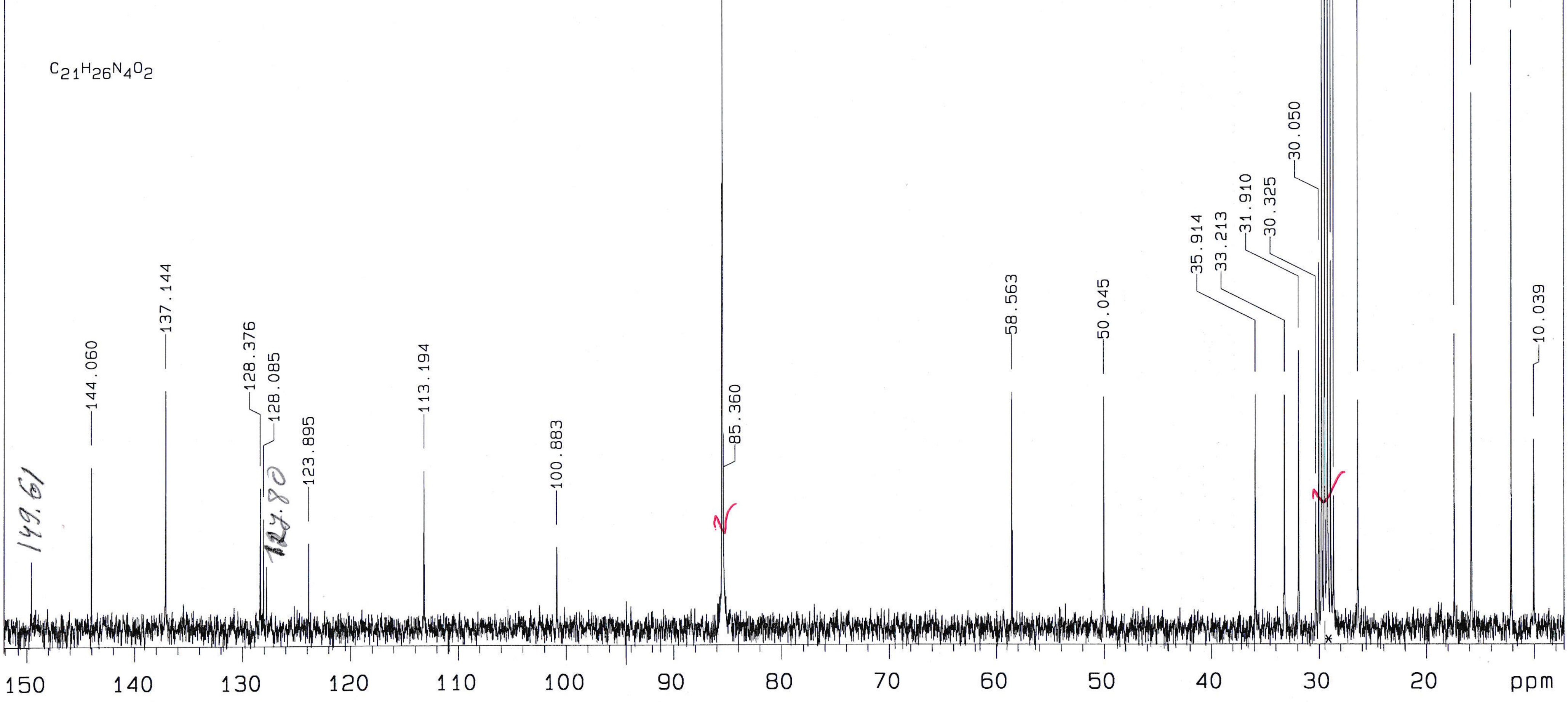

Supplement: Supplementary file 1 [file molecules-24-03952-s001.pdf]
